# Differences in gynecologic tumor development in Amhr2-Cre mice with *KRAS*^*G12D*^ or *KRAS*^*G12V*^ mutations

**DOI:** 10.1038/s41598-020-77666-y

**Published:** 2020-11-26

**Authors:** Eucharist H. S. Kun, Yvonne T. M. Tsang, Sophia Lin, Sophia Pan, Tejas Medapalli, Anais Malpica, JoAnne S. Richards, David M. Gershenson, Kwong-Kwok Wong

**Affiliations:** 1 Department of Gynecologic Oncology and Reproductive Medicine, Unit 1362, The University of Texas MD Anderson Cancer Center, 1515 Holcombe Boulevard, Houston, TX 77030 USA; 2 Departments of Pathology, The University of Texas MD Anderson Cancer Center, Houston, TX USA; 3 Department of Molecular and Cellular Biology, Baylor College of Medicine, Houston, TX USA

**Keywords:** Cancer models, Oncogenesis

## Abstract

How different KRAS variants impact tumor initiation and progression in vivo has not been thoroughly examined. We hypothesize that the ability of either *KRAS*^*G12D*^ or *KRAS*^*G12V*^ mutations to initiate tumor formation is context dependent. Amhr2-Cre mice express Cre recombinase in tissues that develop into the fallopian tubes, uterus, and ovaries. We used these mice to conditionally express either the *KRAS*^*G12V/*+^ *or KRAS*^*G12D/*+^ mutation. Mice with the genotype *Amhr2-Cre Pten*^*(fl/fl)*^* Kras*^*G12D/*+^(G12D mice) had abnormal follicle structures and developed low-grade serous ovarian carcinomas with 100% penetrance within 18 weeks. In contrast, mice with the genotype *Amhr2-Cre Pten*^*(fl/fl)*^* Kras*^*G12V/*+^ (G12V mice) had normal follicle structures, and about 90% of them developed uterine tumors with diverse histological features resembling those of leiomyoma and leiomyosarcoma. Granulosa cell tumors also developed in G12V mice. Differences in cell-signaling pathways in the uterine tissues of G12D and G12V mice were identified using RNA sequencing and reverse-phase protein array analyses. We found that CTNNB1, IL1A, IL1B, TNF, TGFB1, APP, and IL6 had the higher activity in G12V mice than in G12D mice. These mouse models will be useful for studying the differences in signaling pathways driven by *Kras*^*G12V/*+^ or *Kras*^*G12D/*+^ mutations to aid development of targeted therapies for specific *KRAS* mutant variants. Our leiomyoma model driven by the *Kras*^*G12V/*+^ mutation will also be useful in deciphering the malignant progression from leiomyoma to leiomyosarcoma.

## Introduction

Activating *KRAS* mutations are found in more than 20% of all human cancers, including low-grade serous ovarian carcinoma, lung carcinoma, pancreatic cancer, endometrial cancer, and colorectal carcinoma^1–6^. Over the past 3 decades, significant discoveries regarding how wild type and mutant KRAS proteins function as key regulators of signal transduction and drivers of oncogenesis have emerged. Effective anti-KRAS therapies for cancer are now being developed for clinical use. Recent findings suggest that blocking the activity of MEK, a downstream target of KRAS, is an effective way to attack tumors with mutated KRAS proteins that influence MAPK activity^7,8^. Results from clinical trials demonstrate that treatment with MEK inhibitors may improve outcomes in some patients with advanced thyroid or low-grade serous ovarian cancer^9,10^. However, the tumor response rates achieved so far only range from 10 to 20%, and the response durations are usually short, even for tumors with *KRAS* mutations.

G12D, G12V, G12C and G13D are the four predominant *KRAS* mutant protein variants. However, different *KRAS* mutations appear to be associated with distinct clinicopathologic outcomes. Clinical studies have shown that the *KRAS* codon 12 mutation, especially the G12V (c.35G > T) mutation, is potentially associated with poor outcome of colorectal cancer^2,3,11–13^, non-small cell lung cancer^14,15^, and recurrent low-grade serous ovarian carcinoma^16^. However, low-grade serous ovarian carcinomas with the KRAS G12V mutation appeared to have a better clinical outcome than other KRAS variants when treated with MEK inhibitors^16–18^. Because of the biochemical and structural differences among different KRAS mutant variants^19–22^, tumors with these variants may have different downstream signaling pathways. An in vitro study showed that c.35G > T (p.G12V) and c.34G > C (p.G12R) mutations confer a greater ability to transform normal fibroblasts than do other *KRAS* mutations^23^. Furthermore, the GTPase activity of G12V and G12R mutants is lower than that of other *KRAS* mutants^21,22^. Similarly, Lievre et al.^24^ suggested that G12V-mutated cells were insensitive to treatment with cetuximab but that G13-mutated cells were nearly as responsive to cetuximab as colorectal cancer cells with wild-type *KRAS*.

Several in vitro studies have shown that the downstream signaling of G12D and G12V is different in activating the RAF/ERK pathway and PI3K/AKT/mTOR pathway. The G12D variant signals primarily through the PI3K, JNK, p38, and FAK signaling pathways^25,26^ rather than through the RAF/ERK pathway. Furthermore, G12D causes constitutive PI3K/mTOR activity^26^. The G12D variant possesses more oncogenic potential than the wild-type variant but has less oncogenic potential than the G12V variant^25^. However, colon cancer cell lines expressing G12V have elevated RAS-GTP activity (activated RAS), increased RAS signaling and high oncogenic potential^23,27,28^. Thus, the G12V variant is associated with more aggressive cancer behavior than the G12D variant^12,14,15,25^. Furthermore, another study found that, in isogenic MCF10A cell lines with different KRAS mutations, KRAS G12V has a higher level of GTP-bound KRAS and slightly greater anchorage-independent growth associated with more aggressive phenotype than the KRAS G12D and the wild-type variants^29^.

Previously, we found that patients with low-grade serous ovarian carcinoma who carry the KRAS G12V mutation have shorter survival times than those with wild-type KRAS or other KRAS variants^16^. However, how different KRAS variants impact tumor initiation and progression in vivo has not been thoroughly examined. Because we had generated a low-grade serous ovarian carcinoma mouse model with the *Amhr2-Cre Pten*^*(fl/fl)*^* Kras*^*G12D/*+^ genotype (G12D mice) for a previous study^30,31^, we decided to generate mice with the *Amhr2-Cre Pten*^*(fl/fl)*^* Kras*^*G12V/*+^ genotype (G12V mice) for the present prospective investigation. We hypothesized that the ability of the *KRAS*^*G12D*^ or *KRAS*^*G12V*^ mutations to initiate tumor formation would be context dependent as suggested by a previous study^32^. In this previous study, when *KRAS*^*G12V*^ was activated throughout the whole body at a postnatal stage, only a percentage of *KRAS*^*G12V*^-expressing lung bronchiolo-alveolar cells became multiple adenomas and adenocarcinomas and no other tumor types were observed^32^. This result indicates that *KRAS*^*G12V*^ induced tumor development is highly dependent on the cell type as well as on the developmental state when activation occurs. To discover the cellular pathways or the cellular context of a specific cell type to *KRAS* induced tumor development will provide insights into the mechanisms on how malignant transformation occurs in vivo.

## Results

### G12D and G12V mice experienced different effects on follicle development

**Figure 1:**
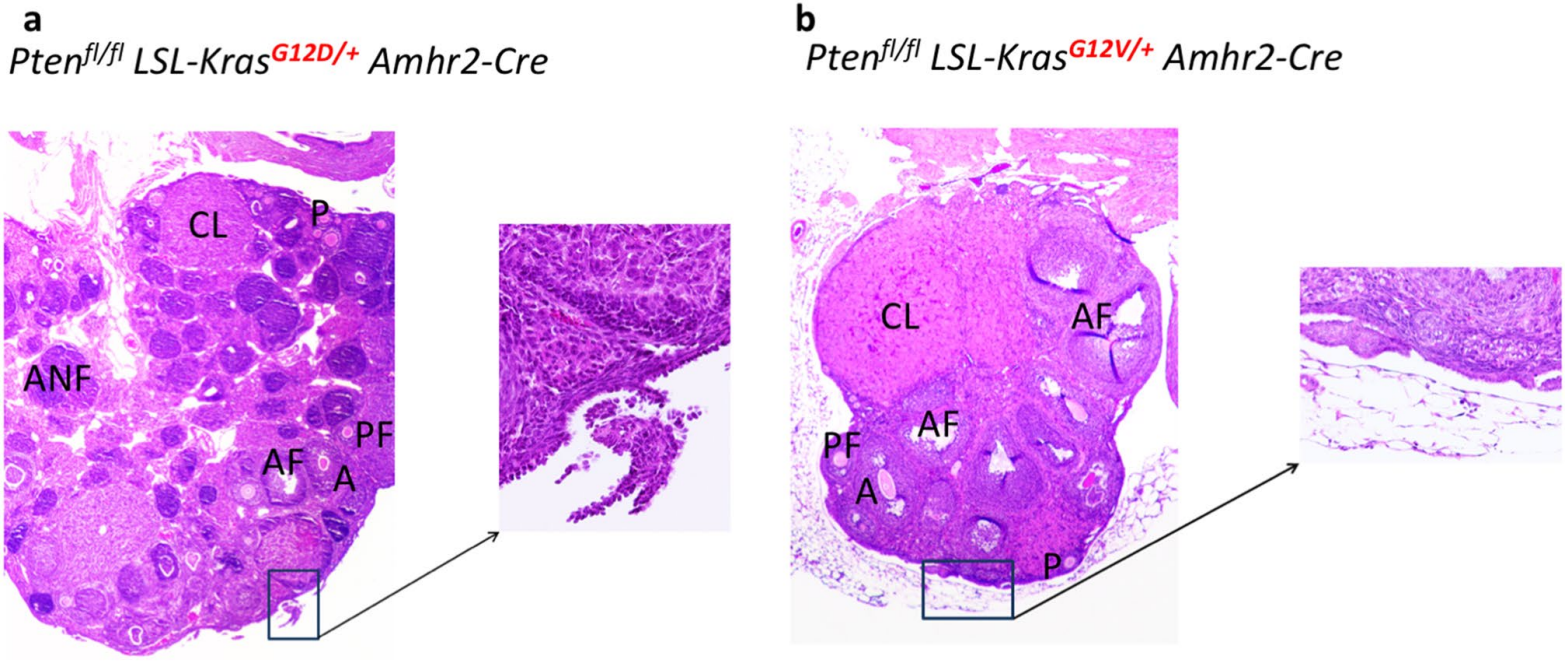
Morphologic comparison of ovaries from 9-week-old (**a**) Pten^fl/fl^ Kras^G12D/+^ Amhr2-Cre (G12D) and (**b**) Pten^fl/fl^ Kras^G12V/+^ Amhr2-Cre (G12V) mice. The ovaries from the G12D mice had substantial defects in follicle development, but those from the G12V mice appeared to have normal follicle development. A, antral follicle; AF, atretic antral follicle; CL, corpus; P, primordial and primary follicle; PF, preantral follicle; ANF, abnormal follicle. The box highlighted epithelial hyperplasia.

**Figure 2:**
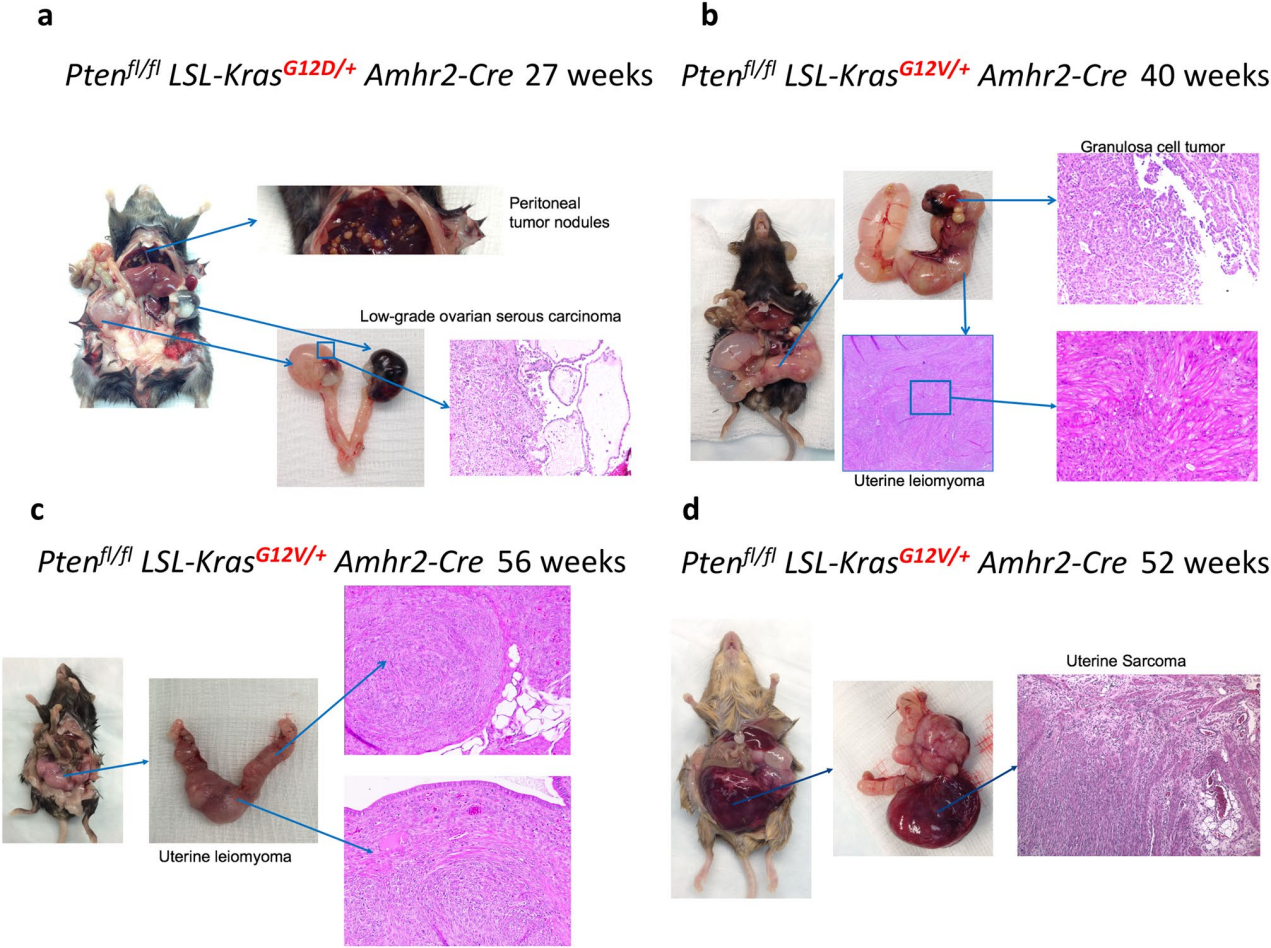
G12D and G12V mice had different types of gynecologic tumors. (**a**) Low-grade serous ovarian carcinoma with metastatic peritoneal tumor nodules developed in G12D mice at week 27. (**b–d**) Ovarian stromal tumors, uterine leiomyoma, and uterine sarcoma developed in G12V mice from week 40 to week 56.

To detect if there were any difference in the impact of Kras^G12D^ and Kras^G12V^ on follicle development, histological evaluation of mouse ovaries were performed. We removed ovaries from both Pten^fl/fl^ Kras^G12D**/+**^ Amhr2-Cre (G12D mice) and Pten^fl/fl^ Kras^G12V**/+**^ Amhr2-Cre (G12V mice) age-matched mice at approximately 9-week-old. The ovaries from the G12D mice exhibited substantial morphologic defects in follicle development with numerous abnormal, follicle-like structures (Fig. 1a) as observed previously^31,33^. In contrast, the ovaries from the G12V mice exhibited normal follicle development; we observed primordial, preantral, antral, and atretic antral follicles as well as corpus lutea in these mice (Fig. 1b). Also, epithelial hyperplasia was also evident on the ovarian surface in both G12D and G12V mice. The ovaries from the G12D mice were approximately 1.5 times larger than those from the G12V mice at 9 weeks; epithelial hyperplasia in G12D mice eventually progressed to low-grade serous carcinomas as shown in Fig. 2a whereas the epithelial hyperplasia in G12V mice did not (see below).

### G12D and G12V mice developed different gynecologic tumors

The anti-Müllerian hormone type II receptor (*Amhr2*) gene is expressed in tissues that develop into the fallopian tubes, uterus, and ovary^34^. Amhr2-Cre is expressed in epithelial cells on the ovarian surface epithelial cells, ovarian stromal cells, and granulosa cells^31^ and in the Müllerian duct mesenchyme-derived endometrial stroma and myometrium, but not in the endometrial epithelium^35^. Using Cre recombinase driven by the promoter of Amhr2, we generated mice with the genotype Pten^fl/fl^ Kras^G12D**/+**^ Amhr2-Cre (G12D mice) or with Pten^fl/fl^ Kras^G12V/**+**^ Amhr2-Cre (G12V mice), in which different types of gynecologic tumors developed (Fig. 2 and Supplementary Fig. S1). We had bred fourteen G12D mice which were sacrificed between five to 38-week-old. Two of the G12D mice older than 27 weeks had low-grade serous ovarian tumors with metastatic peritoneal tumor nodules (Fig. 2a). Previously, we had only reported the phenotype of G12D mice up to the age of 15-week-old and all of them had ovarian low-grade ovarian serous carcinoma by 15-week-old. Here we discovered that metastatic peritoneal nodules could be observed in G12D mice at the age of 27 weeks. On the other hand, we had bred thirty-seven G12V mice which were sacrificed between five to 56-week-old. Three mice of the fifteen G12V mice older than 36-week-old had ovarian “stromal” granulosa cell tumors and uterine leiomyoma (fibroids) (Fig. 2b), all the twenty-two G12V mice older than 26-week-old had uterine leiomyoma (Fig. 2c), and two of the mice older than 52-week-old had uterine leiomyosarcoma (Fig. 2d). Figure 2d shows that, at 52 weeks of age, one of the G12V mice exhibited uterine tumors with leiomyoma region, vascular leiomyosarcoma-like region, and ovarian “stromal” granulosa cell tumor region. Of the 11 G12V mice that we bred and monitored for more than 1 year, only 2 had a phenotype like that of the mouse in Fig. 2d. Additional images of the gross morphology of the gynecologic tumors that developed in G12V mice at different ages are shown in Supplementary Fig. S1. We further confirmed the presence of epithelial ovarian tumors and ovarian “stromal” granulosa cell tumors in these mice by staining ovarian tumor sections for the epithelial marker cytokeratin 8 (Supplementary Fig. S2). The ovarian tumors in G12V mice were cytokeratin 8-negative and inhibin-positive. Because *Amhr2-Cre* and *KRAS G12V* were expressed in the granulosa cells and because they did not express the epithelial maker, the ovarian tumors in the G12V mice are likely to be granulosa cell tumors^36^. The uterine leiomyomas that developed in the G12V mice were hypercellular and highly mitotic according to Ki-67 staining (Supplementary Fig. S3) and they also expressed ESR1 (Supplementary Fig. S4).

### Upregulated pERK in tumor cells during the progression of uterine leiomyoma in G12V mice

**Figure 3:**
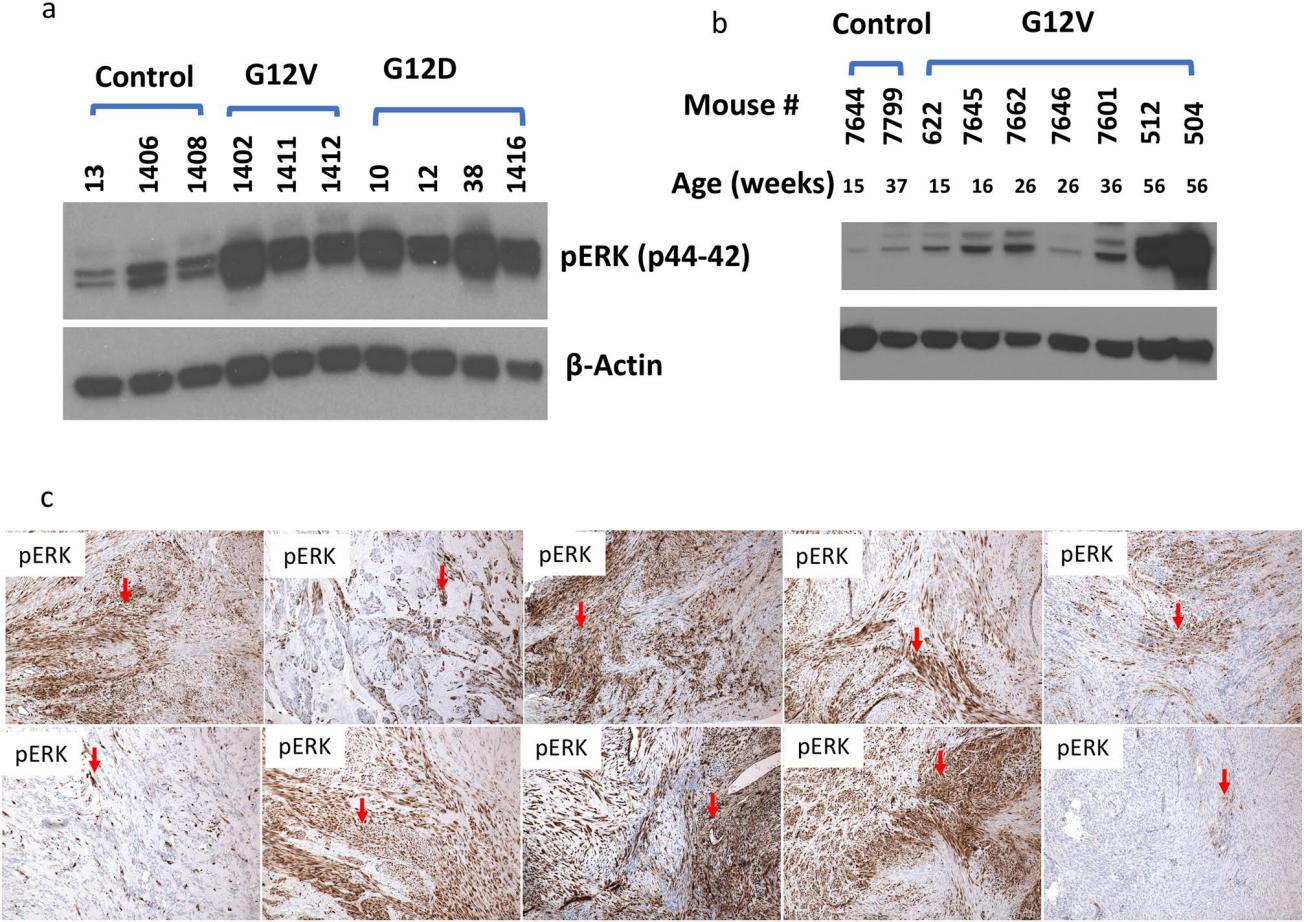
Western blot analysis of pERK expression in uterine tissues from KRAS-mutant mice and immunohistochemical analysis of pERK expression in human leiomyoma. (**a**) Activation of pERK in uterine tissues from G12D and G12V mice. The mice consisted of the following: #13 (14.1 weeks old, Pten^fl/fl^ Amhr2-Cre, control), #10 (14.1 weeks old, G12D), #12 (14.1 weeks old, G12D), #1416 (14.4 weeks old, G12D), #38 (19.6 weeks old, G12D), #1406 (14.7 weeks old, Pten^fl/fl^ Amhr2-Cre, control), #1402 (14.7 weeks old, G12V), #1411 (14.7 weeks old, G12V), and #1412 (14.7 weeks old, G12V). #13, #10, and #12 were littermates, as were #1402, #1411, and #1412**. **(**b**) Upregulation of pERK during uterine tissue progression to leiomyoma in G12V mice**. **(**c**) Immunostaining for pERK in 10 human leiomyoma samples.

To analyze ERK phosphorylation in the tumor, protein extracts were prepared from uterine tissues from G12D and G12V mice and used in Western blot analysis. At about 14 weeks of age, both G12D and G12V mice had higher pERK expression than did their littermate controls (*Pten*^*fl/fl*^* Amhr2-Cre*) that lacked KRAS mutations (Fig. 3a). Moreover, during week 56, when the uterine leiomyomas were at peak development, we observed robust upregulation of pERK protein in G12V mice (Fig. 3b). To determine whether pERK is also upregulated in human leiomyoma, we performed immunostaining for pERK with leiomyoma samples from 10 patients and found that 6 of the samples had robust expression of pERK in all tumor cells (Fig. 3c).

### Reverse-phase protein array data showed that uterine and ovarian tissues from G12D and G12V mice had different signaling pathways

**Figure 4:**
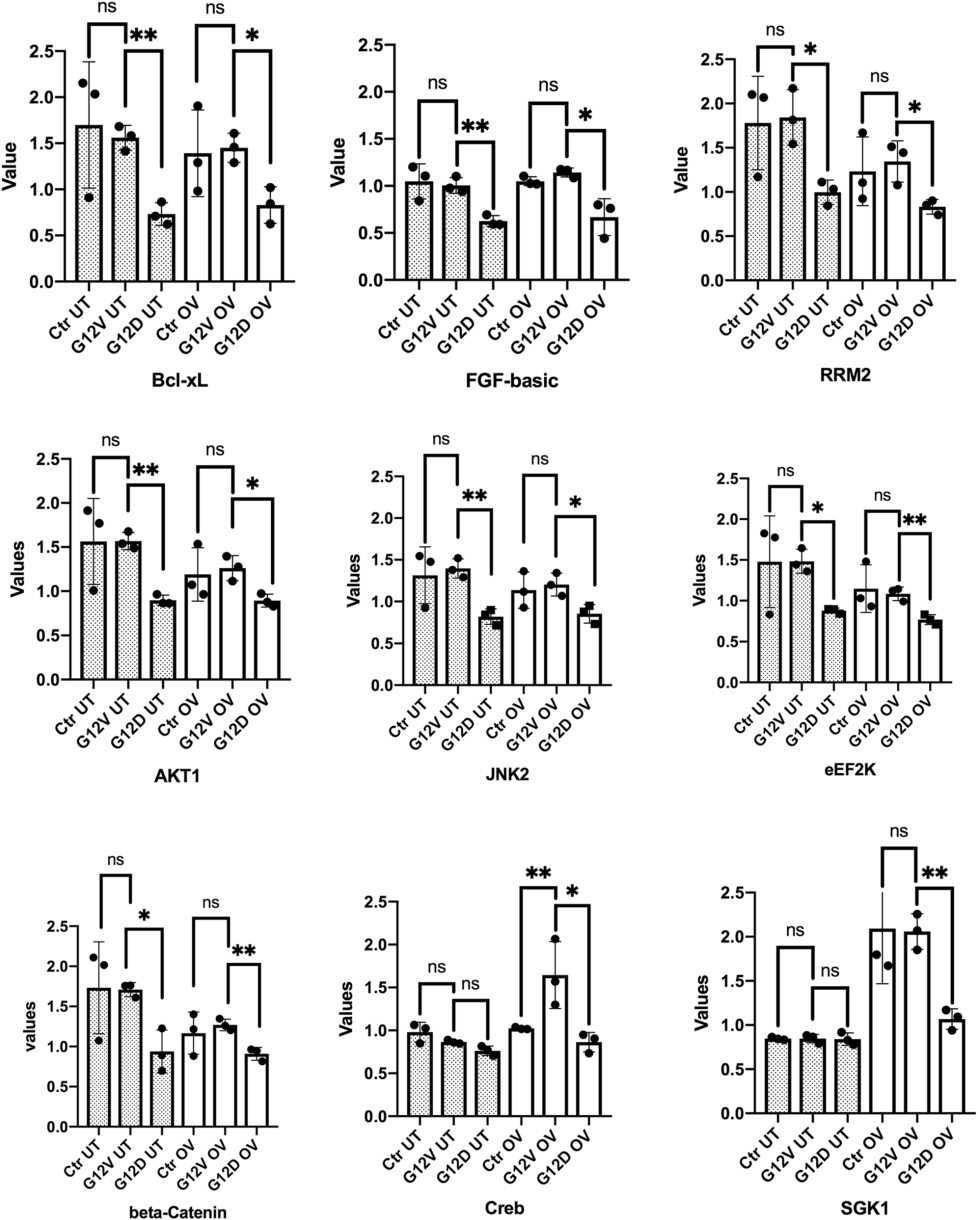
Significantly differentially expressed proteins in the uterine and ovarian tissue of G12D and G12V mice as determined using RPPA analysis. These analyses were performed using protein lysates extracted from the ovarian and uterine tissues of three G12D, three G12V, and three control mice. The values shown are the protein expression levels in the control, G12V and G12D mice. Statistical significance was determined using an unpaired *t*-test with the Welch correction. *p < 0.05; **p < 0.01. ns, not significant; OV, ovarian; UT, uterine. The welch’s t test of significance and figures were generated with GraphPad Prism version 8.0.0 for macOS, GraphPad Software, San Diego, California USA, www.graphpad.com.

**Figure 5:**
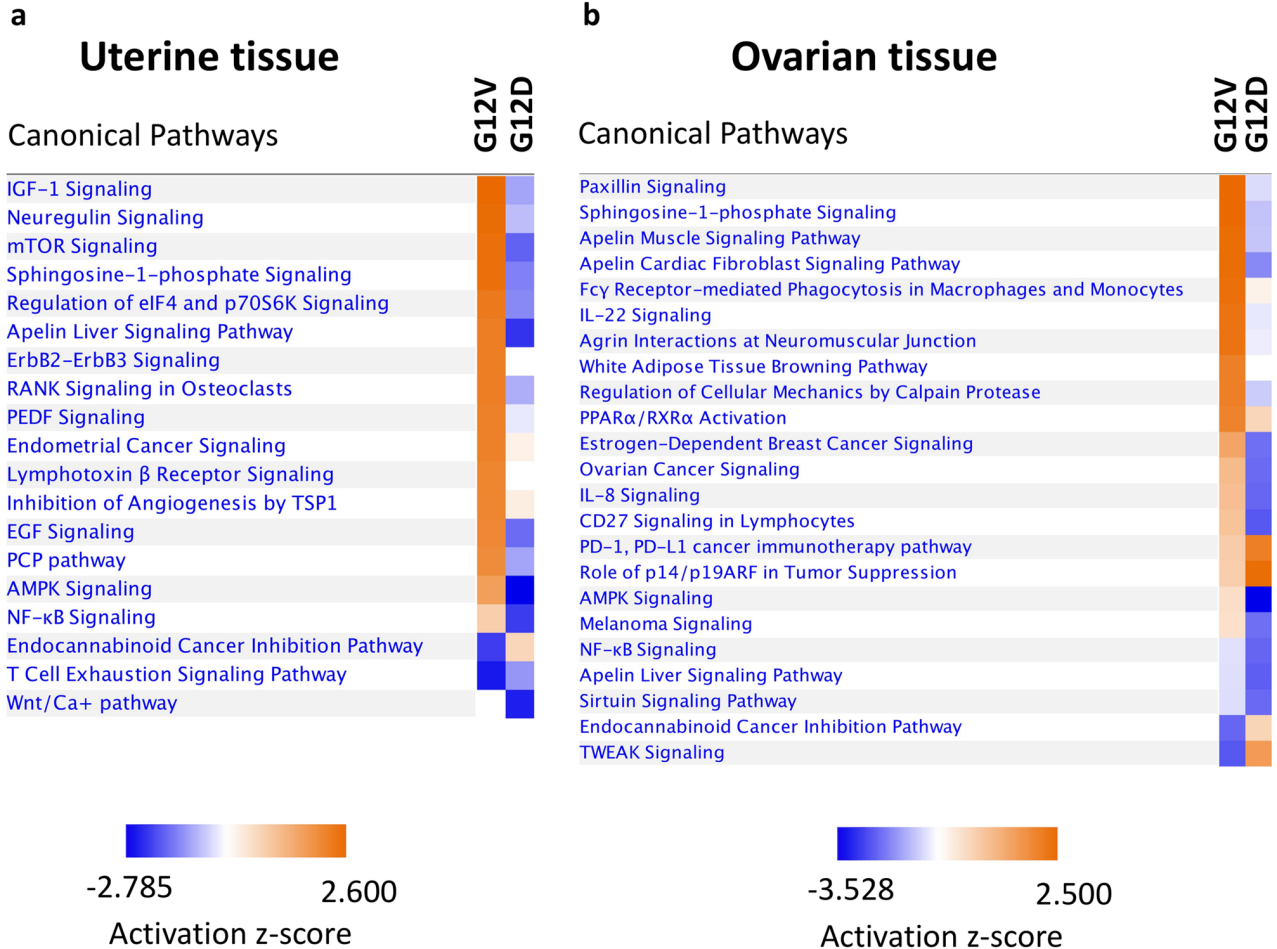
Differences in the activation of canonical pathways in uterine and ovarian tissues from approximately 14-week-old G12D and G12V mice as determined using RPPA analysis. Activation and inhibition of canonical pathways were identified via Ingenuity Pathway Analysis using RPPA data on differentially expressed proteins (Supplementary Table 1) in the ovarian and uterine tissues of three G12D mice, three G12V mice, and three control mice without KRAS mutations. An absolute z-score of 2 implied activation or inhibition of the corresponding pathways. The figures were generated through the use of IPA (QIAGEN Inc., https://www.qiagenbio-informatics.com/products/ingenuity-pathway-analysis).

In our previous study, we have not investigated the signaling pathways in the uterine tissues of G12D mice. Thus, we performed a functional proteomic comparison of ovarian and uterine tissues from approximately 14-week-old G12D mice (n = 3), G12V mice (n = 3), and control mice (Pten^fl/fl^ Amhr2-Cre; n = 3) using reverse-phase protein array (RPPA) analysis with 368 antibodies. We confirmed the expression of mutant *KRAS* mRNA in these mice using reverse transcription polymerase chain reaction (PCR) and the complementary DNA (cDNA) were sequenced by Sanger Sequencing method. Plots of protein expression in uterine and ovarian tissues from G12D and G12V mice using normalized expression values for highly differentially expressed signaling proteins are shown in Fig. 4. We found that G12V mice had markedly higher expression of Bcl-xL, FGF-basic, RRM2, AKT1, JNK2, eEF2K, and beta-Catenin in both ovarian and uterine tissues than did G12D mice except Creb. Creb appeared to have higher expression in G12V mice than G12D mice only in the uterine tissues. When compared to the control tissues, G12D mice had markedly lower expression of Bcl-xL, FGF-basic, RRM2, AKT1, JNK2 , eEF2K, beta-Catenin and SGK1 in both ovarian and uterine tissues except Creb. Ingenuity Pathway Analysis (IPA)-based examination of the canonical pathways in these tissues from G12D and G12V mice revealed major differences in both the activated and inhibited canonical pathways (Fig. 5). In the uterine tissue from G12V mice, the most-activated signaling pathways were those for IGF-1, neuregulin, and mTOR (Fig. 5a). In the ovarian tissue from G12V mice, the most activated signaling pathways were those for paxillin, sphingosine-1-phosphate, and apelin (Fig. 5b). In contrast, in the ovarian tissue from G12D mice, the most activated signaling pathways were those for p14/p19ARF, PD-1/PD-L1, and TWEAK. Raw RPPA data are provided in Supplementary Table S1.

### Differential gene expression in uterine tissues from G12V and G12D mice in comparison with those from control mice according to RNA sequencing analysis

**Figure 6:**
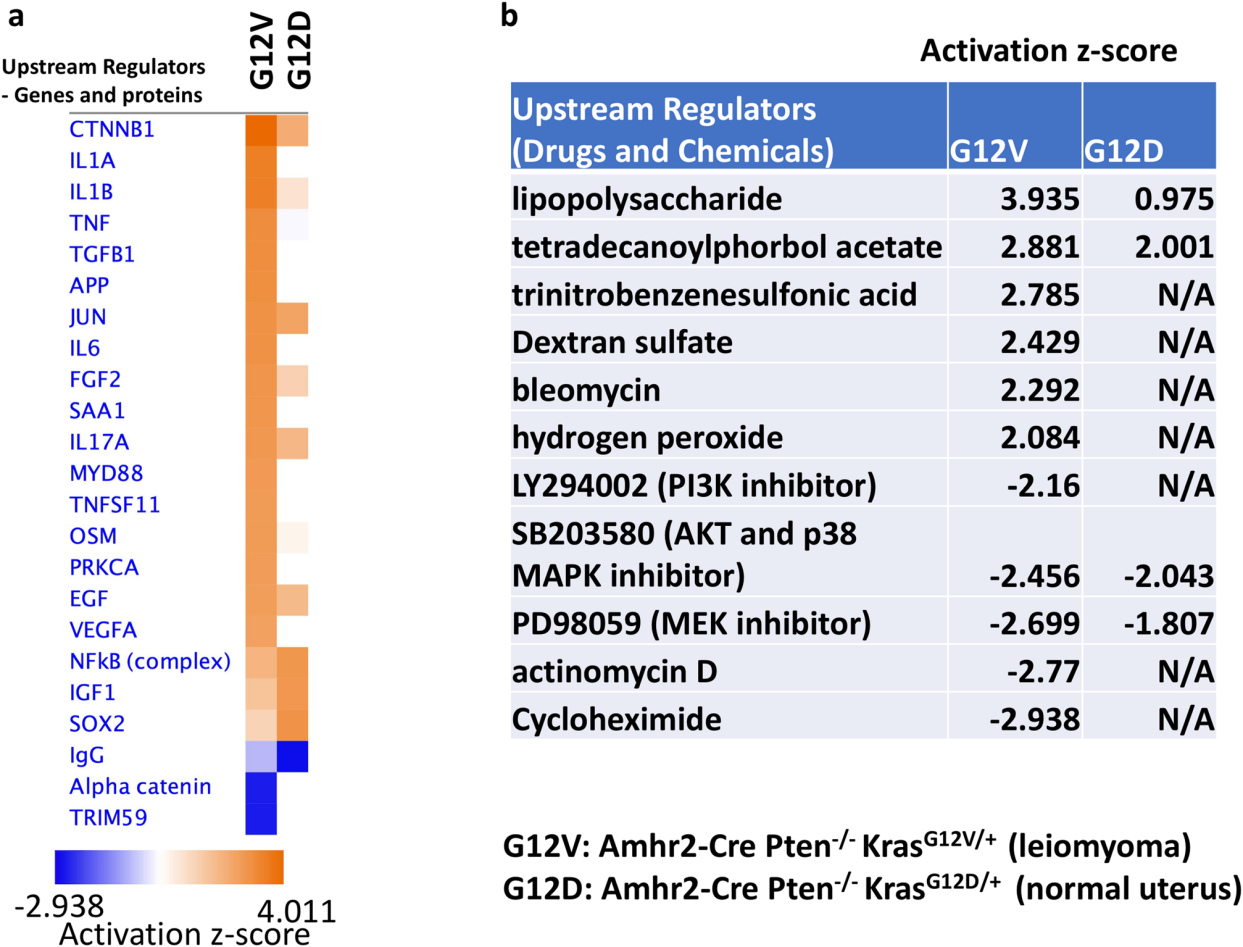
Differences in the activity of upstream regulators of gene networks in uterine tissues from G12D and G12V mice as discovered using RNA-seq analysis. The upstream regulators of gene networks were identified via Ingenuity Pathway Analysis using the uterine tissue gene expression profiles (Supplementary Table S2 and S3) from five G12D mouse (14 to 38-week-old), five G12V mice (15 to 37-week-old), and five control mice with the Amhr2-Cre Pten^fl/fl^ genotype (14 to 66-week-old). The uteri from the G12D and control mice appeared to have normal phenotypes, but the G12V mice had normal phenotype and leiomyoma phenotypes. The most activated and inhibited gene network upstream regulators (absolute z-score > 5) are shown. (**a**) Gene/protein upstream regulators that activate or inhibit the gene networks in G12V mice in comparison with those in G12D mice. (**b**) Drug/chemical upstream regulators that activate or inhibit the gene networks. The figure was generated through the use of IPA (QIAGEN Inc., https://www.qiagenbio-informatics.com/products/ingenuity-pathway-analysis).

We generated RNA sequencing (RNA-seq) data for the total RNA extracted from the uterine tissue of five G12D mice (gross morphology appeared to be normal as the control mice), five G12V mice (4 mice younger than 20 weeks had gross morphology appeared to be the same as control mice, and two mice older than 35 weeks had leiomyoma), and five control mice with the Amhr2-Cre Pten^fl/fl^ genotype (Gene Expression Omnibus database under accession number GSE129520). We performed differential expression analysis among control, G12D and G12V mice using the EdgeR software package^37^; the differentially expressed genes are listed in Supplementary Table S2 and S3. We uploaded these data to the Ingenuity Pathway Analysis application and filtered them for p values less than 0.05 and absolute fold change (G12D vs Control mice or G12V vs Control mice) greater than1.5 for upstream regulator analysis. Figure 6a shows the 23 upstream regulators (genes and proteins) with the highest activation z-scores. Gene networks regulated by CTNNB1, IL1A, IL1B, TNF, TGFB1, APP, and IL6 had the higher activity in G12V mice than in G12D mice. Figure 6b shows the upstream regulators (drugs and chemicals) with absolute z-scores greater than 5. Of note, most of the gene networks in G12V mice were upregulated by lipopolysaccharides, which induced inflammation through the production of cytokines and chemokines or oxidative stress-inducing agents (e.g., tetradecanopylphorbol acetate, hydrogen peroxide). On the other hand, some of the gene networks were downregulated by several kinase inhibitors (MEK, PI3K, and p38 MAPK inhibitors).

### Estrogen receptor expression

Leiomyoma is ESR1 positive and very responsive to estrogen stimulation^38^. Thus, we would like to compare the expression level of ESR1 between G12D and G12V mice. The ESR1 expression level (mean Cq = 22.37 ± 0.55) was higher than that for the housekeeping gene HRPT (mean Cq = 25.55 ± 0.58). Although the average ESR1 expression level appeared to be lower in the uterine tissues from G12D mice than in G12V mice, the differences in its expression among the control, G12D, and G12V mice were not statistically significant (Supplementary Fig. S4).

## Discussion

In this study, we generated G12D and G12V mice in which different gynecologic tumors developed. Whereas G12D mice had low-grade serous ovarian tumors, G12V mice had uterine leiomyoma, uterine leiomyosarcoma, and what appear to be granulosa cell tumors. We are unclear why G12D mice did not develop mesenchymal uterine tumors although previous study had shown that mice with Kras G12D driven by PR-Cre (progesterone receptor promoter) developed endometrial carcinoma from the epithelial cells^39^. Using Amhr2-Cre Pten^fl/fl^ mice, we demonstrated that both conditionally expressed KRAS G12V and G12D mutants could activate the MAPK pathway but have different functional impacts on the pathogenesis of epithelial cells on the ovarian surface, granulosa cells, and uterine mesenchymal cells. The G12D and G12V mice may have had different gynecologic cancers because of observed differences in their activated canonical pathways and gene networks.

From the RNAseq differential gene expression analysis, we have identified 98 genes that are up-regulated in G12V mice and 152 genes that are up-regulated in G12D mice in comparison to the control mice (Supplementary Table S2 and S3). *CXCL3* is one of the five up-regulated genes shared between G12D and G12V mice. Recent study has shown that CXCL3 overexpression can promotes the tumorigenic potential of uterine cervical cancer cells via the MAPK/ERK pathway^40^, and can be a potential therapeutic target for breast cancer^41^. Thus, CXCL3 could be a downstream target of mutant Kras. Other genes such as *BGN*, *FN1*, *Col4A1*, *S100A8* and *COLA2* are only up-regulated in the uterine tissues of G12V mice. These genes have been found to be down-regulated in beta-catenin knockout mice^42^. Blglycan encoded by *BGN* is involved in proimflammatory signal and is up-regulated by IL6^43^. IL6 is up-regulated 19-fold in the uterine tissue of G12V mice in comparison to the control mice. Another interesting gene, *Saa3*, which is highly expressed and up-regulated in G12V mice is upregulated in inflammatory response^44^ and a key protumorigenic mediator of cancer associated fibroblast in pancreatic tumors^45^. These up-regulated genes suggest *Kras*^*G12V*^ mutant somehow cause an inflammatory response in the uterine tissues but not by *Kras*^*G12D*^ mutant.

We observed multiple deregulated signaling pathways, including those for IGF-1, neuregulin, mTOR, TGFB1, CTNNB1, and TNF, in the mouse uterine leiomyomas. Importantly, in human leiomyomas, most of these signaling pathways, such as upregulation of mTOR, TGFB1 and CTNNB1 pathway^46,47^, deregulation of IGF-1 signaling in human uterine leiomyoma^48,49^ and inflammatory process involving TNF signaling^50,51^ have been reported. Thus, the leiomyomas that developed in our G12V mice recapitulated many characteristics of human leiomyoma. Some of the activated pathways we identified may be useful as therapeutic targets for further investigation. For example, in the G12V mice, we observed robust activation of the pERK pathway during leiomyoma progression (Fig. 3b); most human leiomyomas also have high expression of pERK protein (Fig. 3c). Leiomyomas (fibroids) are common benign uterine tumors that afflict more than 35% of women in their reproductive years^52^. Accordingly, MEK and ERK inhibitors should be further investigated as potential treatments of leiomyoma in this G12V mouse as preclinical investigation. Similarly, the highly activated inflammatory gene networks we observed in mouse leiomyomas suggest that anti-inflammatory drugs could be further investigated as a means of preventing the progression of atypical leiomyomas in the mouse model as preclinical investigation.

Another notable observation in our comparison of G12D and G12V mice was that granulosa cell tumors developed in G12V mice but not in G12D mice. Previously, investigators showed that selective expression of Kras^G12D^ in the granulosa cells of the mouse ovary blocks the granulosa cell differentiation pathway during follicle development but does not stimulate the oncogenic transformation of granulosa cells^31,33^. Our results showed that blockage of granulosa cell differentiation may be due to activation of the p14/p19ARF pathway in G12D mice (Fig. 5b). p19ARF is a tumor suppressor that inhibits cell proliferation^53^. However, the expression of KRAS^G12V^ in our G12V mice transformed normal granulosa cells into tumor cells. Comparing the canonical pathways in the ovarian tissues from G12D and G12V mice, we found that the most activated pathway in G12V mice was the paxillin signaling pathway (Fig. 5b). Paxillin signaling is involved in cancer initiation and tumor cell dissemination and survival^54^. Its involvement in the development of granulosa cell tumors should be investigated further, even though, Kras mutations are not common in these tumors.

Leiomyomas are classified as usual leiomyomas or atypical leiomyomas (cellular leiomyoma, mitotically active leiomyoma, and smooth muscle tumors of uncertain malignant potential). The most common gene mutation in usual leiomyomas is that of *MED12,* which researchers have detected in 43% of leiomyoma samples^55^. The second most common mutations detected in leiomyomas are *KRAS* (4% [3/72])^56–59^ and *TP53* (4% [5/113])^56,60^ mutations. Interestingly, all the three *KRAS*-mutated leiomyomas had the same KRAS^Q61H^^56^. Structural analysis of the different *KRAS* mutations indicated that the G12V and Q61H mutations cause similar conformational change of K-Ras4B-GDP protein but G12D mutation causes larger conformational changes and results in a higher exposure of the nucleotide-binding site than G12V and Q61H mutations^61^. Thus, although G12V has not been found in a small number of leiomyoma, it is possible that G12V has similar oncogenic potential as Q61H found in leiomyoma. While KRAS mutations are not very common in leiomyomas, KRAS gene expression has been upregulated in more than 50% of leiomyomas^62^. Our immunohistochemical testing of human leiomyoma samples showed that these samples had high levels of pERK expression. In addition, we found that the leiomyomas that developed in the G12V mice were hypercellular and highly mitotic according to Ki-67 staining (Supplementary Fig. S3) and ESR1-positive (Supplementary Fig. S4). Although leiomyoma rarely develops into leiomyosarcoma, molecular and immunohistochemical data demonstrate that leiomyosarcomas can arise from some subtypes of leiomyoma, especially the cellular subtype^63^. Recent clinical and molecular genetic evidences indicate that some uterine leiomyosarcomas might evolve from preexisting atypical leiomyomas such as cellular leiomyoma, mitotically active leiomyoma or smooth muscle tumor of uncertain malignant potential (STUMP)^60,64–66^. This has caused a major concern in the risk of occult leiomyosarcoma found at surgery using a minimally invasive approach performed laparoscopically for presumed benign fibroids^66–68^. The progression of G12V mice with atypical leiomyoma to leiomyosarcoma is also rare. Only two of the eleven G12V mice that we bred and monitored for more than 1 year had leiomyosarcoma phenotype like that of the mouse shown in Fig. 2d. Therefore, our G12V mice had the phenotype of human atypical leiomyoma with a potential to progress as leiomyosarcoma. Hill et al. has also identified *KRAS* mutations in 14% (7/51) of leiomyosarcomas; these mutations are associated with worse survival than those without *KRAS* mutations^69^. These seven KRAS mutations were of different types—one G12V, four G12C, one G12F and one G13V^69^. Interesting, no G12D was found this set of leiomyosarcoma. Loss of the tumor suppressor gene *PTEN* combined with *KRAS* mutation are more common in leiomyosarcomas than in leiomyoma. Deletion of 10q, which contains *PTEN*^70^ and *PTEN* mutations in uterine leiomyosarcomas^71^ have also been observed. Likewise, activation of the PI3K/AKT signaling pathway, which is inhibited by PTEN activity, occurs in leiomyosarcomas^72^. Thus, our G12V mice with *PTEN* loss and *KRAS* G12V mutations recapitulated the heterogeneity of human atypical leiomyoma that can progress to leiomyosarcoma. This uterine leiomyoma model will be useful for studying relevant oncogenes, signaling pathways, and other cellular changes involved in the malignant progression of leiomyoma. It can also be used for the preclinical testing of different therapeutic agents, such as MEK, PI3K, and mTOR inhibitors. Because uterine leiomyomas typically develop during the reproductive years and tend to regress after menopause^73–76^, we plan to use these mouse models to understand how estrogen, progesterone, steroid hormone receptors, growth factors, and growth factor receptors impact tumor initiation and progression.

## Methods

### Animals

LSL-Kras^G12D/+^ and Amhr2-Cre, Pten^fl/fl^ mice were obtained from Dr. JoAnne Richards at Baylor College of Medicine. LSL-Kras^G12V/+^ mice were obtained from The University of Texas MD Anderson Cancer Center Genetically Engineered Mouse Facility, where frozen sperm from a mouse heterozygous for LSL-Kras^G12V/+^ (a gift from Dr. Mariano Barbacid at the Spanish National Cancer Research Center) was used for in vitro fertilization of female C57BL6/J mice. These parental strains were used to derive Pten^fl/fl^, LSL-Kras^G12D/+^; Pten^fl/fl^, LSL-Kras^G12V/+^; Amhr2-Cre, Pten^fl/fl^; Amhr2-Cre, Pten^fl/fl^, LSL-Kras^G12D/+^; and Amhr2-Cre, Pten^fl/fl^, LSL-Kras^G12V/+^ mice. Genotyping was done using PCR as described previously^32,34,77,78^. Animals were housed in the Michale E. Keeling Center for Comparative Medicine and Research at MD Anderson, provided food and water ad libitum, and kept on a 16-h light/8-h dark schedule. Animals were treated in accordance with the NIH Guide for the Care and Use of Laboratory Animals and as approved by the Institutional Animal Care and Use Committee at MD Anderson. Since Kras^G12V^ allele and β-gal gene were co-activated by Amhr2-Cre, we performed immunostaining of the β-gal in the fallopian tube, ovarian and uterine tissues in the G12V mice to confirm the expression of Cre in those tissues (Supplementary Fig. S5). Furthermore to confirm the deletion of exon 5 in the Pten transcript, we aligned the sequencing reads with the full length Pten mRNA and also performed western blot analysis of Pten in the uterine tissues (Supplementary Fig. S6).

### Histology and immunohistochemistry

Whole mouse ovaries and uteri were collected, fixed in 4% paraformaldehyde, embedded in paraffin, and processed using routine procedures^33^. Human leiomyoma samples were obtained after informed consent and stored in accordance with the human subject research protocols approved by the MD Anderson Institutional Review Board for this study. Formalin-fixed, paraffin-embedded (FFPE) leiomyoma blocks were obtained from the tumor repository in the Department of Gynecologic Oncology and Reproductive Medicine at MD Anderson. For immunohistochemistry, five micron FFPE sections were cut parrafin from blocks. Deparaffinization and heat-induced antigen retrieval of FFPE sections were performed using Lab Vision PT Module (Thermo Fisher Scientific) using citrate buffer (pH = 6) at 97 °C for 20 min. Then the slides were treated with hydrogen peroxide for 10 min followed by Lab Vision blocking reagent for 5 min. After blocking, the slides were incubated with the targeted antibody with the dilution described in the antibody section below for one hour. Detection signal was then generated by incubating with EnVision HRP reagent (Agilent) for 30 min followed by Lab Vision DAB regent for five minuties. Counterstaining of the nuclei was performed with Hematoxylin for 1 min. The immunostaining process was processed with Lab Vision Autostainer 360-2D (ThermoFisher Scientific).

### Western blot analysis

Tissue samples were lysed with RIPA buffer (150 mM NaCl, 1.0% IGEPAL CA-630, 0.5% sodium deoxycholate, 0.1% SDS, 50 mM Tris, pH 8.0; Sigma, St. Louis, MO) with a protease inhibitor cocktail (Sigma) and Halt phosphatase inhibitor cocktail (Thermo Fisher Scientific, Waltham, MA). The samples were then homogenized, and their protein concentrations were measured using a Bradford assay with a FLUOstar Omega Microplate Reader (BMG Labtech Inc., Cary, NC). Extracted proteins (25 µg) were resolved via 1-D polyacrylamide gel electrophoresis using Mini-PROTEAN TGX Precast Gels (Bio-Rad Laboratories, Hercules, CA) and then transferred to PDVF membranes (Trans-Blot Turbo Mini PVDF Transfer Packs; Bio-Rad Laboratories) using a Trans-Blot Turbo Transfer System (Bio-Rad Laboratories). Membranes were incubated first with primary antibodies for an hour, washed 3 times for 8 min each, and then with secondary antibodies for an hour, again followed with 3 washes for 8 min each. They were then visualized with a chemiluminescent HRP antibody detection reagent (HyGLO Quick Spray; Denville Scientific, Holliston, MA) using film in a darkroom.

### Antibodies

The following primary antibodies were used in this study: anti-p44/42 MAPK (Erk1/2), rabbit polyclonal (cat. #4370S; Cell Signaling Technology, Beverly, MA) at a 1:1000 dilution; goat anti-rabbit IgG HRP (cat. #7074S; Cell Signaling Technology) at a 1:3000 dilution; anti-mouse IgG Kappa HRP, mouse monoclonal (cat. #SC-516102; Santa Cruz Biotechnology, Inc., Dallas, TX) at a 1:5000 dilution; anti-β-actin (13E5), rabbit monoclonal (cat. # 4970S; Cell Signaling Technology) at a 1:1000 dilution; anti-inhibin alpha antibody, rabbit polyclonal (cat. # ab216969; Abcam, Cambridge, UK) at a 1:100 dilution; and anti-mouse cytokeratin 8 (Krt8) rat IgG monoclonal (TROMA-I; Developmental Studies Hybridoma Bank, University of Iowa, Iowa City, IA ) at a 1:150 dilution.

### Reverse transcription PCR and cDNA sequencing

Total RNA was extracted from mouse uterine tissues using a PureLink RNA Minikit (Ambion cat#12183018A) to confirm the expression of KRAS^G12V^ mutant transcripts using reverse transcription PCR and cDNA sequencing. Quality of RNA was confirmed to have a 260/280 wavelength absorption ratio of approximately 2.0 with the use of a NanoDrop Spectrophotometer (ND-1000). cDNA was generated from 1 ug of total RNA using a High-Capacity cDNA Reverse Transcription Kit (Thermo Fisher Scientific). The cDNA region with the KRAS mutation was amplified via PCR using mouse-specific KRAS primers (forward primer, 5′-AGGCCTGCTGAAAATGACTG; reverse primer, 5′-CCCTCCCCAGTTCTCATGTA). PCR was performed using an ABI Geneamp 9700 PCR machine at 95 °C for 1 min, followed by 35 cycles at 95 °C for 15 s, 62 °C for 15 s, and 72 °C for 10 s, and at 72 °C for 5 min with a final hold at 4 °C. PCR products were purified with QiAquick PCR purification kit (Qiagen) and sequenced using Sanger Sequencing at the MD Anderson Advanced Technology Genomics Core.

### RPPA

Protein lysates were extracted from both the whole ovaries and uterine tissues of nine 14-week-old mice with the following genotypes: Amhr2-Cre Pten^fl/fl^ Kras^G12V^, Amhr2-Cre Pten^fl/fl^ Kras^G12D^, and Amhr2-Cre Pten^fl/fl^ (three mice per genotype). For 40 mg of tissue in a 5 ml tube on ice, 1 ml of ice-cold lysis buffer was added. Lysis buffer contain 1% Triton X-100, 50 mM HEPES, pH 7.4, 150 mM NaCl, 1.5 mM MgCl2, 1 mM EGTA, 100 mM NaF, 10 mM Na pyrophosphate, 1 mM Na3VO4, 10% glycerol, containing freshly added protease and phosphatase inhibitors from Roche Applied Science Cat. # 05056489001 and 04906837001, respectively. The tumor tissue was then homogenized by an electric homogenizer for 8 s.

Subsequently, samples were transferred to microcentrifuge tubes and centrifuged at 4 °C, 14,000 rpm for 10 min. Supernatant (protein lysates) were collected and transferred to another set of microcentrifuged tubes. Protein concentration was determined by Bradford reaction and adjusted to a concentration of 1.5 μg/μl. These 18 ovary and uterine tissue protein lysates were probed with 367 antibodies at the MD Anderson Functional Proteomics RPPA Core laboratory. Each tissue lysate sample was serially diluted (undiluted, followed by dilutions in buffer at 1:2, 1:4, 1:8, and 1:16 ratios). Samples were then arrayed on nitrocellulose-coated slides in an 11 × 11 format to produce sample spots. Next, the sample spots were probed with antibodies using a tyramide-based signal amplification approach and were visualized using a DAB colorimetric reaction. Stained slides were scanned using a TissueScope scanner (Huron Digital Pathology, St. Jacobs, Ontario, Canada) to produce 16-bit TIFF images. Relative protein levels for each sample were then determined based on the five-dilution sample spots using an R script (https://bioinformatics.mdanderson.org/public-software/supercurve/) written in the MD Anderson Department of Bioinformatics and Computational Biology. Functional analysis of differentially expressed genes in the G12D and G12V mouse tissues was performed with the Ingenuity Pathway Analysis software application (QIAGEN) for Fig. 5.

### RNA-seq analysis

Total RNA was extracted with a PureLink RNA Mini Kit (Thermo Fisher Scientific) from the uterine tissues of five G12D mouse (14 to 38-week-old), five G12V mice (15 to 37-week-old), and five control mice with the Amhr2-Cre Pten^fl/fl^ genotype (14 to 66-week-old). The RNA quality was checked with BioAnalyzer (Agilent) and RNA with an RIN greater than 7 will only be used for cDNA sequencing and RNAseq. Sequencing libraries were prepared using a KAPA Stranded mRNA Seq Kit (Roche Sequencing and Life Science Kapa Biosystems, Wilmington, MA) and were run in a single lane using a HiSeq 4000 or NextSeq500 instrument (Illumina, San Diego, CA) in pair-end mode with a mean depth of 94 million reads of 76 bp for each sample. All Illumina Fastq files were processed with an RNA-seq workflow and a CLC Genomics Workbench (version 11; QIAGEN, Germantown, MD). Reads were mapped to the mouse reference genome GRCm38 using the default setting. Gene expression was estimated using the expectation–maximization estimation algorithm and reported as transcripts per million^79^. Functional analysis of differentially expressed genes in the G12D and G12V mouse uterine tissues was performed with the Ingenuity Pathway Analysis software application (QIAGEN).

### Reverse transcription quantitative PCR analysis

Mouse uterine tissue was preserved in RNAlater solution (Thermo Fisher Scientific) and stored at − 20 °C. RNA was extracted from the tissue with a PureLink RNA Mini Kit (Thermo Fisher Scientific), and DNA was removed from it with a PureLink DNase Set (Thermo Fisher Scientific). The concentration of the extracted RNA was measured using a NanoDrop spectrophotometer (Thermo Fisher Scientific), and the RNA was stored at − 80 °C. cDNA synthesis of the RNA samples was then performed using a High Capacity cDNA Reverse Transcription Kit (Applied Biosystems, Foster City, CA). Next, the samples were incubated for 2 h at 37 °C using a PCR machine (Thermo Fisher Scientific; cat. #ABI9700). Quantitative reverse transcription PCR was performed with 2X iTaq Universal Probes Supermix (Bio-Rad; cat. #172-5131) and Taqman Gene Expression Assays for ESR1 (Thermo Fisher Scientific; cat. #Mm00433149_m1) and HPRT (Thermo Fisher Scientific; cat. #Mm00446968_m1) as a control. The quantitative reverse transcription PCR run itself was performed using a CFX96 Thermal Cycler (Bio-Rad) under optimized conditions as determined by Thermo Fisher Scientific for its Taqman Gene Expression Assays. We measured the RNA expression for the estrogen receptor ESR1 in uterine tissues from approximately 14-week-old mice (five control mice, four G12D mice, and three G12V mice) using quantitative reverse transcription PCR TaqMan Gene Expression assays and the HPRT gene as a normalization control. The qPCR data was normalized by subtracting the Cq value of HPRT from the Cq value of the corresponding Cq value of ESR1 in the same sample as ΔCq and the log transformed as expression value (Expression value = Power (2, − ΔCq). Based on the RNAseq data, the expression values (TPM) of HPRT gene across all 15 samples were 88 ± 22 which is quite invariable to be used for normalization.

## Supplementary information

Supplementary information.

Supplementary Legends.

## Data Availability

All data generated or analyzed during this study are included in the published article (and its Supplementary Information file). RNAseq data has been deposited in Gene Expression Omnibus database under accession number GSE129520.

## References

[1] Wong KK (2009). Recent developments in anti-cancer agents targeting the Ras/Raf/ MEK/ERK pathway. Recent Pat. Anti-Cancer Drug Discov..

[2] Brink M (2003). K-ras oncogene mutations in sporadic colorectal cancer in The Netherlands Cohort Study. Carcinogenesis.

[3] Neumann J, Zeindl-Eberhart E, Kirchner T, Jung A (2009). Frequency and type of KRAS mutations in routine diagnostic analysis of metastatic colorectal cancer. Pathol. Res. Pract..

[4] Kim ST (2011). Impact of KRAS mutations on clinical outcomes in pancreatic cancer patients treated with first-line gemcitabine-based chemotherapy. Mol. Cancer Ther..

[5] Cancer Genome Atlas Research Network (2013). Integrated genomic characterization of endometrial carcinoma. Nature.

[6] Arrieta O (2015). Updated frequency of EGFR and KRAS mutations in non-small cell lung cancer (NSCLC) in Latin America: The Latin-American Consortium for the Investigation of Lung Cancer (CLICaP). J. Thorac. Oncol..

[7] Schmitz KJ (2007). Activation of extracellular regulated kinases (ERK1/2) but not AKT predicts poor prognosis in colorectal carcinoma and is associated with k-ras mutations. Virchows Arch..

[8] Mahapatra DK, Asati V, Bharti SK (2017). MEK inhibitors in oncology: A patent review (2015-present). Expert Opin. Ther. Pat..

[9] Hayes DN (2012). Phase II efficacy and pharmacogenomic study of selumetinib (AZD6244; ARRY-142886) in iodine-131 refractory papillary thyroid carcinoma with or without follicular elements. Clin. Cancer Res..

[10] Farley J (2013). Selumetinib in women with recurrent low-grade serous carcinoma of the ovary or peritoneum: An open-label, single-arm, phase 2 study. Lancet Oncol..

[11] Guerrero S (2000). K-ras codon 12 mutation induces higher level of resistance to apoptosis and predisposition to anchorage-independent growth than codon 13 mutation or proto-oncogene overexpression. Cancer Res..

[12] Andreyev HJ, Norman AR, Cunningham D, Oates JR, Clarke PA (1998). Kirsten ras mutations in patients with colorectal cancer: The multicenter “RASCAL” study. J. Natl. Cancer Inst..

[13] Winder T (2009). Different types of K-Ras mutations are conversely associated with overall survival in patients with colorectal cancer. Oncol. Rep..

[14] Vega F (1996). Association of K-ras codon 12 transversions with short survival in non-small cell lung cancer. Int. J. Oncol..

[15] Keohavong P (1996). Detection of K-ras mutations in lung carcinomas: Relationship to prognosis. Clin. Cancer Res..

[16] Tsang YT (2013). KRAS (but not BRAF) mutations in ovarian serous borderline tumor are associated with recurrent low-grade serous carcinoma. J. Pathol..

[17] Han C, Bellone S, Zammataro L, Schwartz PE, Santin AD (2018). Binimetinib (MEK162) in recurrent low-grade serous ovarian cancer resistant to chemotherapy and hormonal treatment. Gynecol. Oncol. Rep..

[18] Takekuma M, Wong KK, Coleman RL (2016). A long-term surviving patient with recurrent low-grade serous ovarian carcinoma treated with the MEK1/2 inhibitor, selumetinib. Gynecol. Oncol. Res. Pract..

[19] Chen CC (2013). Computational analysis of KRAS mutations: Implications for different effects on the KRAS p.G12D and p..G13D mutations. PLoS ONE.

[20] Muraoka S (2012). Crystal structures of the state 1 conformations of the GTP-bound H-Ras protein and its oncogenic G12V and Q61L mutants. FEBS Lett..

[21] Al-Mulla F, Milner-White EJ, Going JJ, Birnie GD (1999). Structural differences between valine-12 and aspartate-12 Ras proteins may modify carcinoma aggression. J. Pathol..

[22] Bollag G, McCormick F (1995). Intrinsic and GTPase-activating protein-stimulated Ras GTPase assays. Methods Enzymol..

[23] Seeburg PH, Colby WW, Capon DJ, Goeddel DV, Levinson AD (1984). Biological properties of human c-Ha-ras1 genes mutated at codon 12. Nature.

[24] Lievre A (2006). KRAS mutation status is predictive of response to cetuximab therapy in colorectal cancer. Cancer Res..

[25] Cespedes MV (2006). K-ras Asp12 mutant neither interacts with Raf, nor signals through Erk and is less tumorigenic than K-ras Val12. Carcinogenesis.

[26] Ihle NT (2012). Effect of KRAS oncogene substitutions on protein behavior: Implications for signaling and clinical outcome. J. Natl. Cancer Inst..

[27] Janakiraman M (2010). Genomic and biological characterization of exon 4 KRAS mutations in human cancer. Cancer Res..

[28] Smith G (2010). Activating K-Ras mutations outwith ‘hotspot’ codons in sporadic colorectal tumours—Implications for personalised cancer medicine. Br. J. Cancer.

[29] Stolze B, Reinhart S, Bulllinger L, Frohling S, Scholl C (2015). Comparative analysis of KRAS codon 12, 13, 18, 61, and 117 mutations using human MCF10A isogenic cell lines. Sci. Rep..

[30] Mullany LK (2011). Molecular and functional characteristics of ovarian surface epithelial cells transformed by KrasG12D and loss of Pten in a mouse model in vivo. Oncogene.

[31] Fan HY (2009). Cell type-specific targeted mutations of Kras and Pten document proliferation arrest in granulosa cells versus oncogenic insult to ovarian surface epithelial cells. Cancer Res..

[32] Guerra C (2003). Tumor induction by an endogenous K-ras oncogene is highly dependent on cellular context. Cancer Cell.

[33] Fan HY (2008). Selective expression of KrasG12D in granulosa cells of the mouse ovary causes defects in follicle development and ovulation. Development.

[34] Arango NA (2008). A mesenchymal perspective of Mullerian duct differentiation and regression in Amhr2-lacZ mice. Mol. Reprod. Dev..

[35] Jamin SP, Arango NA, Mishina Y, Hanks MC, Behringer RR (2002). Requirement of Bmpr1a for Mullerian duct regression during male sexual development. Nat. Genet..

[36] Liu Z, Castrillon DH, Zhou W, Richards JS (2013). FOXO1/3 depletion in granulosa cells alters follicle growth, death and regulation of pituitary FSH. Mol. Endocrinol..

[37] Robinson MD, McCarthy DJ, Smyth GK (2010). edgeR: A bioconductor package for differential expression analysis of digital gene expression data. Bioinformatics.

[38] Borahay MA (2017). Estrogen receptors and signaling in fibroids: Role in pathobiology and therapeutic implications. Reprod. Sci..

[39] Kim TH (2010). The Synergistic effect of conditional pten loss and oncogenic K-ras mutation on endometrial cancer development occurs via decreased progesterone receptor action. J. Oncol..

[40] Qi YL (2020). CXCL3 overexpression promotes the tumorigenic potential of uterine cervical cancer cells via the MAPK/ERK pathway. J. Cell. Physiol..

[41] See AL, Chong PK, Lu SY, Lim YP (2014). CXCL3 is a potential target for breast cancer metastasis. Curr. Cancer Drug Targets.

[42] Youssef KK (2012). Adult interfollicular tumour-initiating cells are reprogrammed into an embryonic hair follicle progenitor-like fate during basal cell carcinoma initiation. Nat. Cell Biol..

[43] Schaefer L (2005). The matrix component biglycan is proinflammatory and signals through Toll-like receptors 4 and 2 in macrophages. J. Clin. Investig..

[44] Tomita T, Ieguchi K, Sawamura T, Maru Y (2015). Human serum amyloid A3 (SAA3) protein, expressed as a fusion protein with SAA2, binds the oxidized low density lipoprotein receptor. PLoS ONE.

[45] Djurec M (2018). Saa3 is a key mediator of the protumorigenic properties of cancer-associated fibroblasts in pancreatic tumors. Proc. Natl. Acad. Sci. USA.

[46] Borahay MA, Al-Hendy A, Kilic GS, Boehning D (2015). Signaling pathways in leiomyoma: Understanding pathobiology and implications for therapy. Mol. Med..

[47] Ali M, Shahin SM, Sabri NA, Al-Hendy A, Yang Q (2020). Activation of beta-catenin signaling and its crosstalk with estrogen and histone deacetylases in human uterine fibroids. J. Clin. Endocrinol. Metab..

[48] Gao Z, Matsuo H, Wang Y, Nakago S, Maruo T (2001). Up-regulation by IGF-I of proliferating cell nuclear antigen and Bcl-2 protein expression in human uterine leiomyoma cells. J. Clin. Endocrinol. Metab..

[49] Van der Ven LT (1997). Expression of insulin-like growth factors (IGFs), their receptors and IGF binding protein-3 in normal, benign and malignant smooth muscle tissues. Br. J. Cancer.

[50] Ciebiera M (2018). TNF-alpha serum levels are elevated in women with clinically symptomatic uterine fibroids. Int. J. Immunopathol. Pharmacol..

[51] Protic O (2016). Possible involvement of inflammatory/reparative processes in the development of uterine fibroids. Cell Tissue Res..

[52] Cramer SF, Patel A (1990). The frequency of uterine leiomyomas. Am. J. Clin. Pathol..

[53] Weber JD (2000). p53-independent functions of the p19(ARF) tumor suppressor. Genes Dev..

[54] Deakin NO, Pignatelli J, Turner CE (2012). Diverse roles for the paxillin family of proteins in cancer. Genes Cancer.

[55] Tate JG (2019). COSMIC: The catalogue of somatic mutations in cancer. Nucleic Acids Res..

[56] Hall KL (1997). Analysis of Ki-ras, p53, and MDM2 genes in uterine leiomyomas and leiomyosarcomas. Gynecol. Oncol..

[57] Rao UN, Finkelstein SD, Jones MW (1999). Comparative immunohistochemical and molecular analysis of uterine and extrauterine leiomyosarcomas. Mod. Pathol..

[58] Patrikis MI (2003). Mutation analysis of CDP, TP53, and KRAS in uterine leiomyomas. Mol. Carcinog..

[59] Tworek H (1999). Mutation analysis of BRCA1, TP53, and KRAS2 in ovarian and related pelvic tumors. Cancer Genet. Cytogenet..

[60] Zhang Q (2014). Molecular analyses of 6 different types of uterine smooth muscle tumors: Emphasis in atypical leiomyoma. Cancer.

[61] Lu S, Jang H, Nussinov R, Zhang J (2016). The structural basis of oncogenic mutations G12, G13 and Q61 in small GTPase K-Ras4B. Sci. Rep..

[62] Zolfaghari N (2017). Identification of differentially expressed K-Ras transcript variants in patients with leiomyoma. Reprod. Sci..

[63] Mittal KR (2009). Molecular and immunohistochemical evidence for the origin of uterine leiomyosarcomas from associated leiomyoma and symplastic leiomyoma-like areas. Mod. Pathol..

[64] Di Luigi G (2015). Leiomyosarcoma: A rare malignant transformation of a uterine leiomyoma. Eur. J. Gynaecol. Oncol..

[65] Hodge JC, Pearce KE, Clayton AC, Taran FA, Stewart EA (2014). Uterine cellular leiomyomata with chromosome 1p deletions represent a distinct entity. Am. J. Obstet. Gynecol..

[66] Pritts EA, Parker WH, Brown J, Olive DL (2015). Outcome of occult uterine leiomyosarcoma after surgery for presumed uterine fibroids: A systematic review. J. Minim. Invasive Gynecol..

[67] Rosenbaum L (2016). N-of-1 policymaking-tragedy, trade-offs, and the demise of morcellation. N. Engl. J. Med..

[68] Yorganci A (2020). Incidence and outcome of occult uterine sarcoma: A multi-centre study of 18604 operations performed for presumed uterine leiomyoma. J. Gynecol. Obstet. Hum. Reprod..

[69] Hill MA (1997). Detection of K-ras mutations in resected primary leiomyosarcoma. Cancer Epidemiol. Biomark. Prev..

[70] Segal NH (2003). Classification and subtype prediction of adult soft tissue sarcoma by functional genomics. Am. J. Pathol..

[71] Amant F (2002). PTEN mutations in uterine sarcomas. Gynecol. Oncol..

[72] Hernando E (2007). The AKT-mTOR pathway plays a critical role in the development of leiomyosarcomas. Nat. Med..

[73] Andersen J (1996). Growth factors and cytokines in uterine leiomyomas. Semin. Reprod. Endocrinol..

[74] Mozzachio K, Moore AB, Kissling GE, Dixon D (2016). Immunoexpression of steroid hormone receptors and proliferation markers in uterine leiomyoma and normal myometrial tissues from the miniature pig, *Sus scrofa*. Toxicol. Pathol..

[75] Strissel PL (2007). Transcriptional analysis of steroid hormone receptors in smooth muscle uterine leiomyoma tumors of postmenopausal patients. J. Steroid Biochem. Mol. Biol..

[76] Reshkin S, Albarani V, Pezzetta A, Marinaccio M, Paradiso A (1997). Gonadotrophin releasing hormone (GnRH) receptor and steroid receptors in human uterine leiomyoma, myometrium and endometrium. Int. J. Oncol..

[77] Jackson EL (2001). Analysis of lung tumor initiation and progression using conditional expression of oncogenic K-ras. Genes Dev..

[78] Groszer M (2001). Negative regulation of neural stem/progenitor cell proliferation by the Pten tumor suppressor gene in vivo. Science.

[79] Li B, Dewey CN (2011). RSEM: Accurate transcript quantification from RNA-Seq data with or without a reference genome. BMC Bioinform..

