# Supplementary material for: Differences in gynecologic tumor development in Amhr2-Cre mice with KRASG12D or KRASG12V mutations
Source: Sci Rep. 2020 Nov 26;10:20678. doi: 10.1038/s41598-020-77666-y (PMC7693266; doi:10.1038/s41598-020-77666-y)

Differences in gynecologic tumor development in Amhr2-Cre mice with *KRAS*^{G12D} or *KRAS*^{G12V} mutations

Eucharist HS Kun[1], Yvonne TM Tsang[1], Sophia Lin[1], Sophia Pan[1], Tejas Medapalli[1], Anais Malpica[2], JoAnne S Richards[3], David M Gershenson[1], Kwong-Kwok Wong[1]

Departments of [1]Gynecologic Oncology and Reproductive Medicine and [2]Pathology, The University of Texas MD Anderson Cancer Center, Houston, Texas, USA

[3]Department of Molecular and Cellular Biology, Baylor College of Medicine, Houston, Texas, USA

Corresponding author:

Kwong-Kwok Wong, Ph.D.

Department of Gynecologic Oncology and Reproductive Medicine, Unit 1362

The University of Texas MD Anderson Cancer Center

1515 Holcombe Boulevard, Houston, TX 77030, USA

Phone: 713-792-0229

Email: kkwong@mdanderson.org

Running title: Gynecologic tumors in Kras mutant mice

Conflict of interest:

The authors declare no potential conflicts of interest.

Supplementary Fig. S1

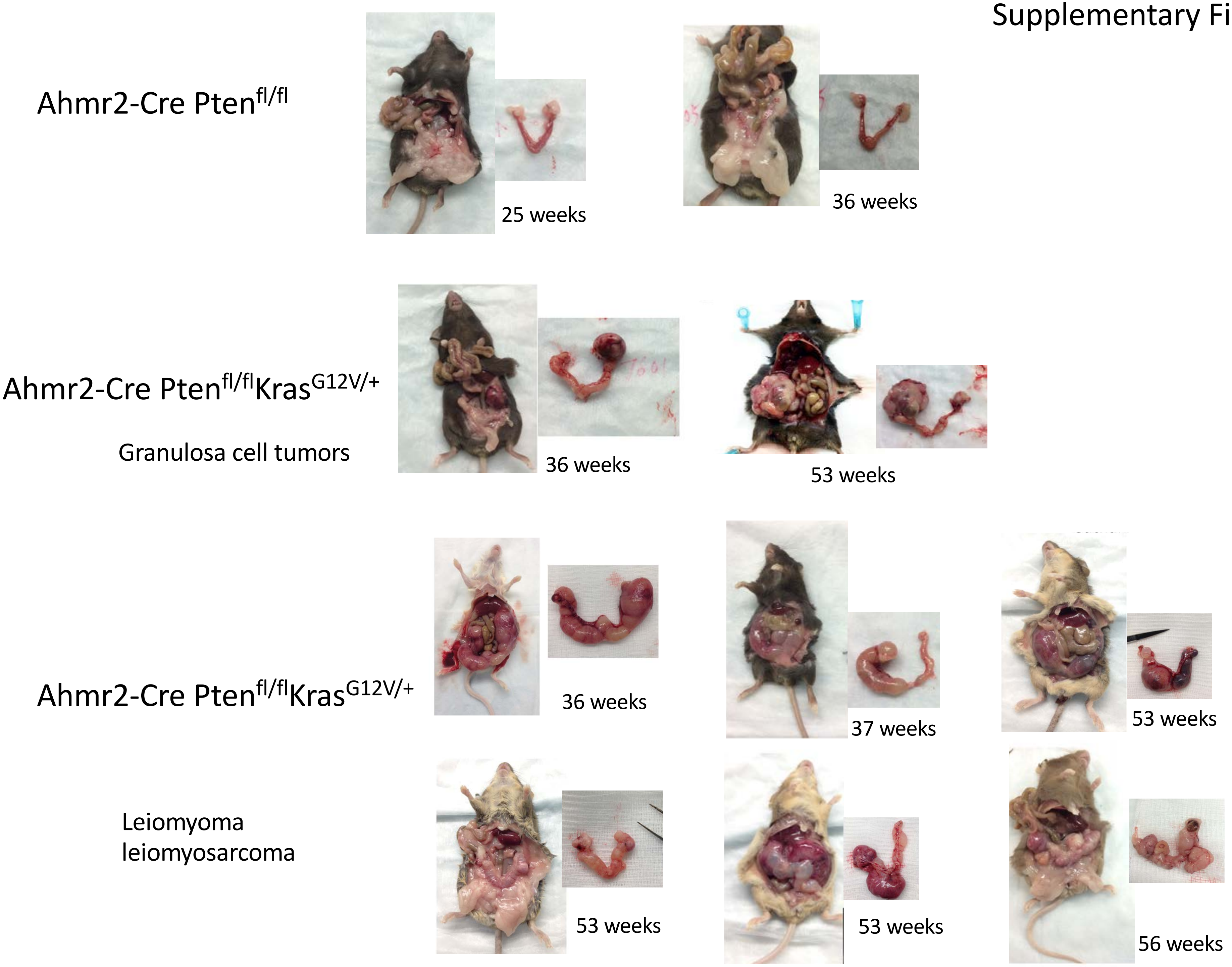

Supplementary Fig. S2

Pten^fl/fl LSL-Kras^G12D Amhr2-Cre (low-grade ovarian serous carcinoma) –cytokeratin positive

Pten^fl/fl LSL-Kras^G12V Amhr2-Cre (Granulosa cell tumor)-cytokeratin negative, inhibin positive

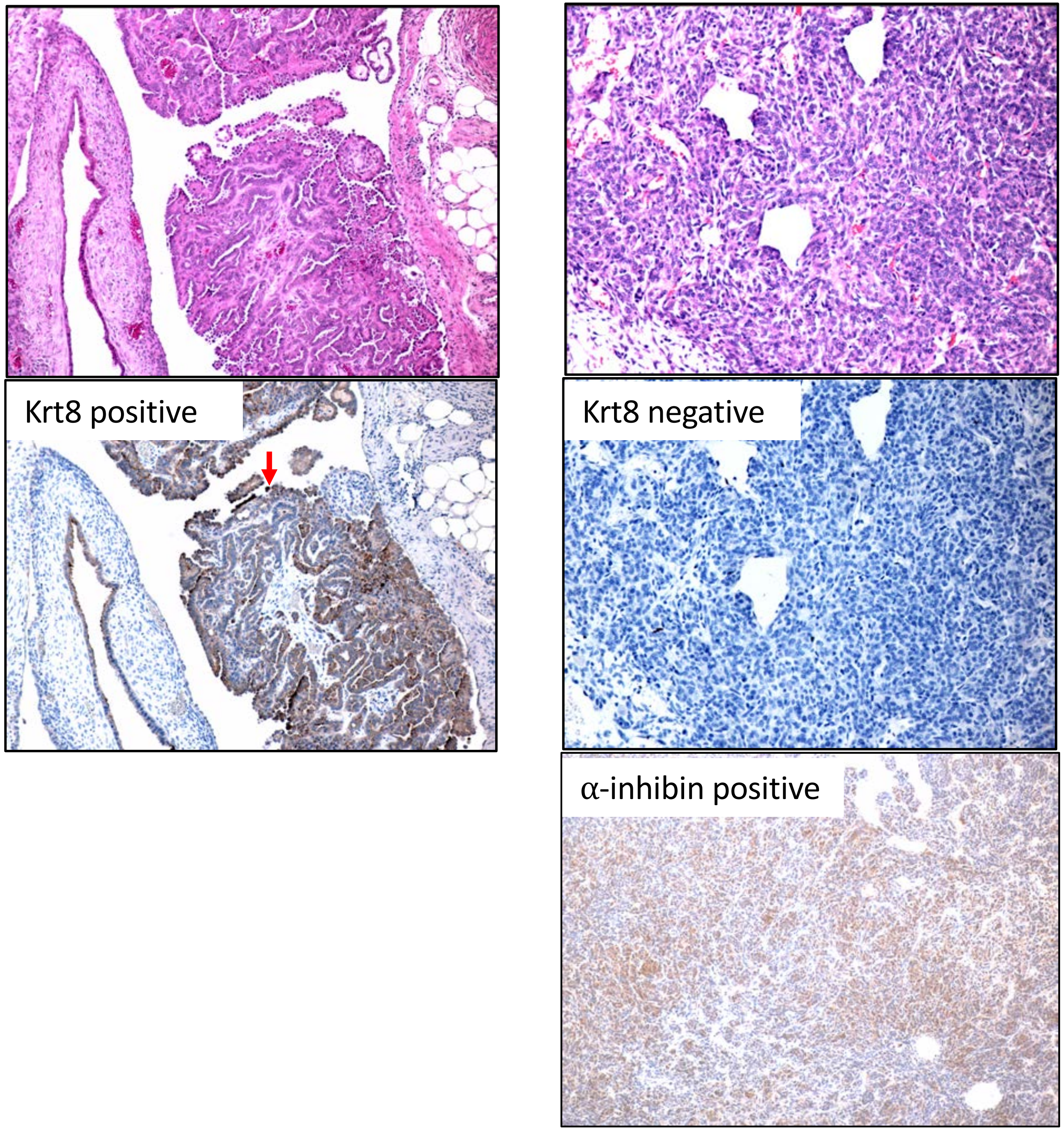

H&E

IHC with Krt8 antibody

IHC with α-inhibin antibody

Supplementary Fig. S3

Leiomyoma gross morphology

Ki-67 immunostaining

Mouse #504, 56 weeks

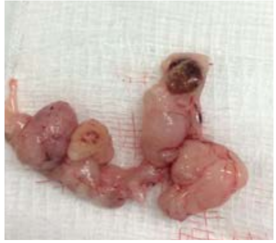

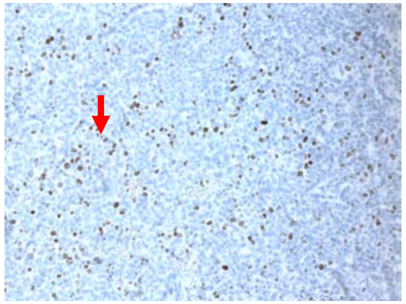

Mouse #569, 36 weeks

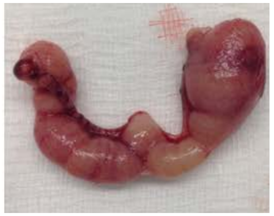

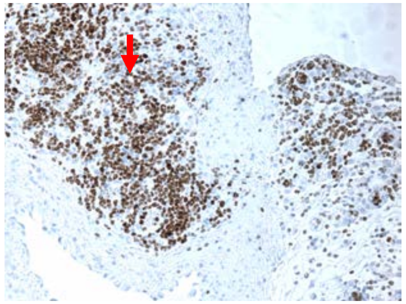

Mouse #2983, 53 weeks

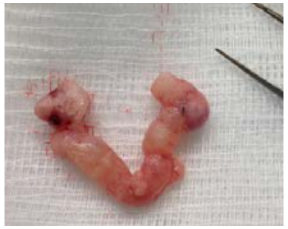

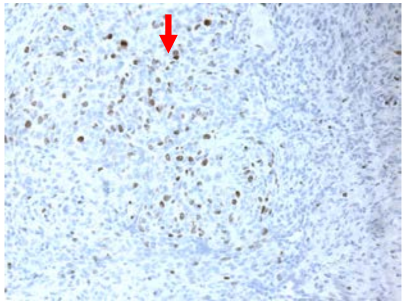

Mouse #2986, 53 weeks

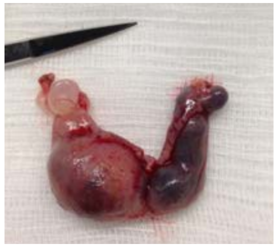

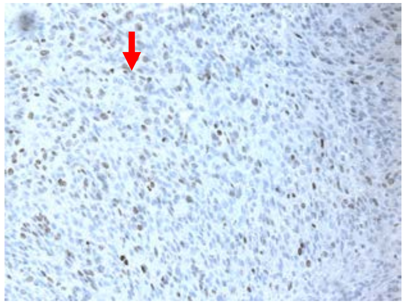

Supplementary Fig. S4

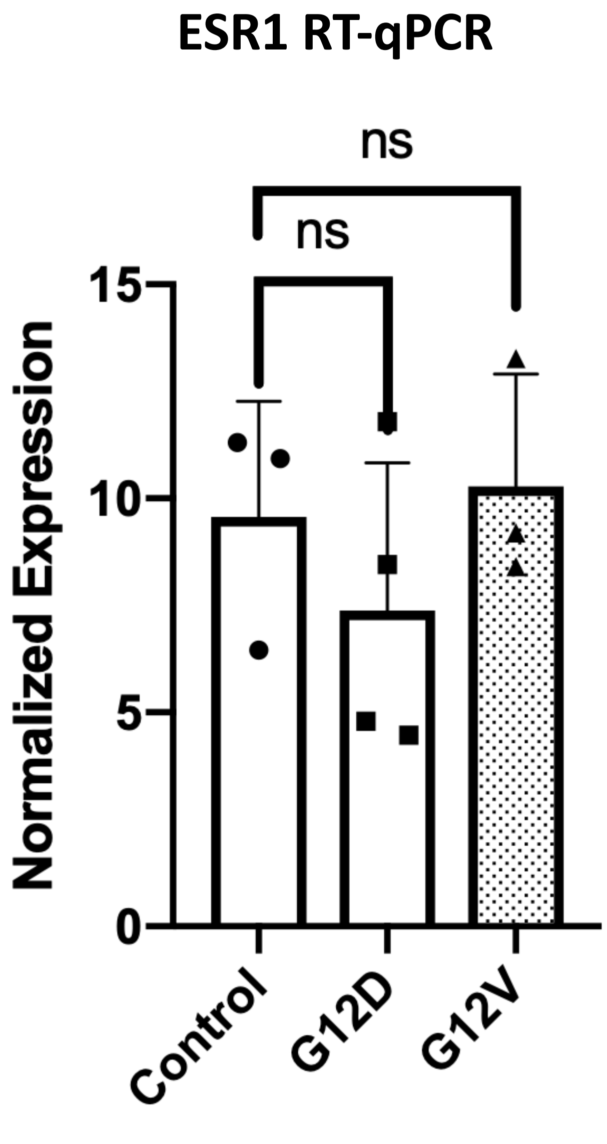

Beta-galactosidase Immunostaining

Fallopian tube

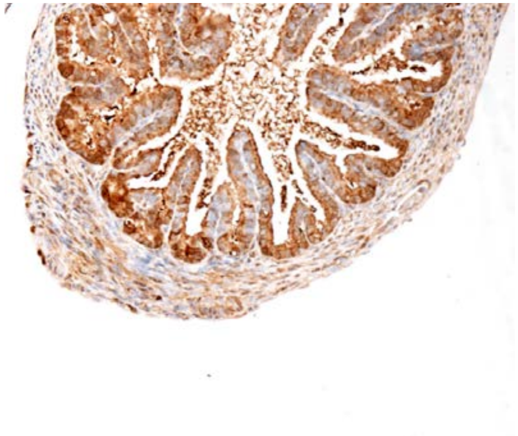

Ovarian tissue

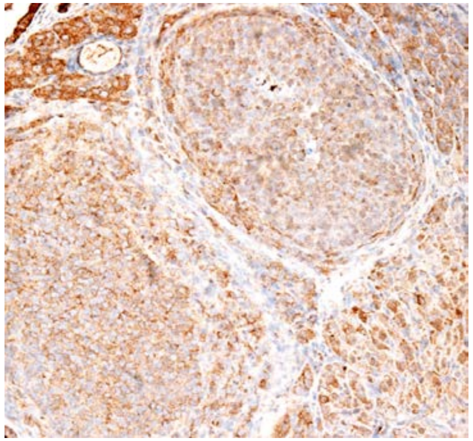

Uterine tissue

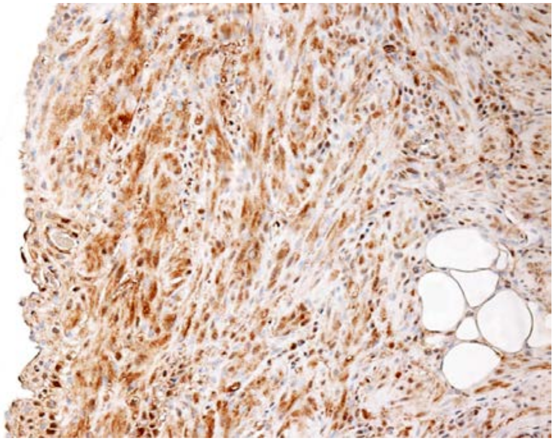

a

Amhr2-Cre Pten $^{fl/fl}$ Amhr2-Cre Pten $^{fl/+}$

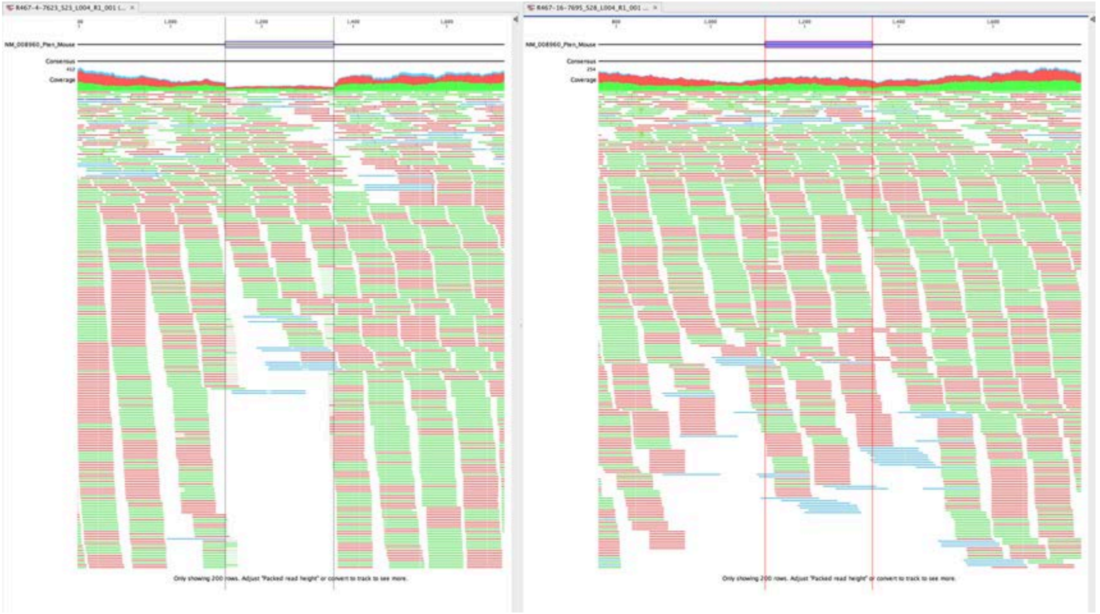

126/412 (30%) 178/254 (70%)

b

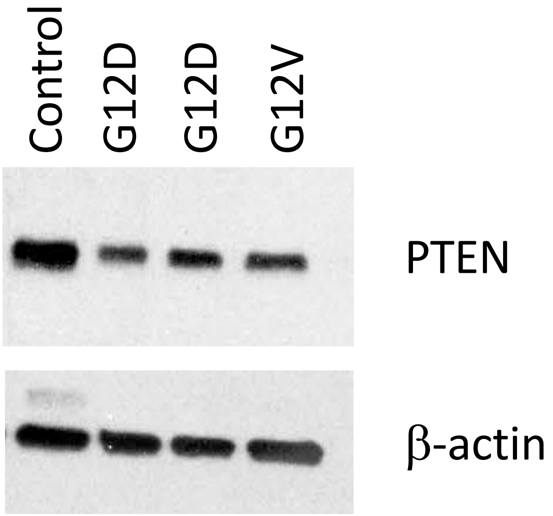

Control: Pten $^{fl/fl}$ KRAS$^{G12Dfl/+}$

G12D: Amhr2-Cre Pten $^{fl/fl}$ KRAS$^{G12D/+}$

G12V: Amhr2-Cre Pten $^{fl/fl}$ KRAS$^{G12V/+}$

Supplementary Table S1. RPPAprotein expression data for G12D, G12V,and control mice

	A-Cre Pten fl/fl KRASG12V fl/+ (G12V)						A-Cre Pten fl/fl KRASG12D fl/+ (G12D)						A-Cre Pten fl/fl (N)						G12V/N		G12D/N	
Antibodies	1402UT	1411UT	1412UT	1402OV	1411OV	1412OV	1416UT	10UT	12UT	1416OV	10OV	12OV	1406UT	13UT	1408UT	1406OV	13OV	1408OV	G12V/N (OV)	G12V/N (UT)	G12D/N (OV)	G12D/N (UT)
S6_pS240_S244-R-V	0.917	1.146	5.574	1.140	1.840	1.809	1.316	1.201	0.695	0.966	1.127	0.913	1.040	2.131	1.792	0.869	0.908	1.373	1.520	1.539	0.954	0.647
Akt_pS473-R-V	3.344	1.113	2.250	2.547	0.741	1.082	1.194	1.519	0.931	1.041	1.483	1.121	0.754	0.764	1.873	0.943	0.808	1.277	1.443	1.978	1.203	1.074
S6_pS235_S236-R-V	0.842	0.929	2.767	1.110	1.576	1.641	1.513	1.339	0.746	1.023	1.291	0.990	0.828	1.647	1.168	0.770	0.898	1.413	1.404	1.246	1.072	0.988
eEF2-R-C	1.862	1.487	3.717	0.922	1.831	1.717	0.670	0.970	0.916	0.566	0.774	0.794	2.854	1.183	2.925	2.042	0.932	1.030	1.116	1.015	0.533	0.367
VAV1-R-C	1.579	2.509	1.671	1.513	0.444	1.220	0.709	0.588	3.594	2.511	0.482	2.478	1.653	0.613	2.078	0.378	1.451	0.472	1.381	1.326	2.379	1.126
Bcl-xL-R-V	1.683	1.416	1.583	1.291	1.607	1.457	0.621	0.710	0.864	0.626	0.845	1.020	2.154	0.911	2.034	1.905	0.982	1.290	1.042	0.918	0.596	0.431
GSK-3a-b_pS21_S9-R-V	1.236	1.162	1.531	1.633	0.648	0.853	0.439	1.025	0.971	0.532	1.364	0.893	1.305	1.350	1.508	0.927	0.994	1.013	1.069	0.944	0.951	0.585
b-Catenin-R-V	1.758	1.609	1.761	1.259	1.203	1.346	0.696	0.893	1.226	0.816	0.964	0.946	2.109	1.074	2.015	1.402	0.882	1.215	1.088	0.987	0.779	0.541
WIPI1-R-C	1.295	1.146	1.836	1.023	1.340	1.280	1.657	0.888	0.952	0.530	0.787	0.873	1.515	0.965	1.678	1.447	0.836	1.137	1.065	1.029	0.641	0.841
MSH6-R-C	1.786	1.653	2.665	1.060	1.509	1.569	0.880	1.211	1.168	0.678	0.894	0.957	2.333	1.193	2.379	1.769	0.910	1.032	1.115	1.034	0.681	0.552
GCLC-R-C	1.113	0.966	1.387	0.979	1.563	1.379	0.670	0.759	0.699	0.658	0.945	1.002	1.622	0.859	1.721	1.932	1.087	1.109	0.950	0.825	0.631	0.506
Stat5a-R-V	0.962	0.912	1.179	1.107	1.558	1.591	0.555	1.050	0.983	0.400	0.908	0.688	0.895	1.173	0.869	1.221	1.316	1.143	1.157	1.040	0.542	0.881
Akt1-R-V	1.676	1.488	1.539	1.113	1.276	1.394	0.862	0.863	0.964	0.972	0.829	0.876	1.914	1.008	1.769	1.535	0.964	1.072	1.059	1.002	0.749	0.573
MDM2_pS166-R-V	1.274	0.960	1.590	0.923	1.249	1.138	0.630	0.887	0.785	0.515	0.832	0.672	1.644	0.913	1.401	1.730	0.975	1.054	0.880	0.966	0.537	0.582
RPA32_pS4_S8-R-C	1.427	1.199	1.792	0.991	1.440	1.321	0.715	1.050	1.092	0.565	0.963	0.978	1.741	1.143	1.749	1.619	0.891	1.111	1.037	0.953	0.692	0.616
Akt_pT308-R-V	2.047	1.201	2.100	1.990	0.871	1.133	1.130	1.359	1.101	1.041	1.228	1.163	1.039	1.107	1.800	0.942	0.921	1.128	1.336	1.356	1.148	0.910
RRM2-R-C	1.814	1.542	2.171	1.078	1.511	1.446	0.844	1.112	1.032	0.740	0.854	0.901	2.067	1.170	2.101	1.669	0.924	1.106	1.091	1.036	0.675	0.560
Tyro3-R-V	1.414	1.298	1.677	1.078	1.279	1.185	1.193	1.096	1.015	0.738	1.004	1.001	1.594	1.147	1.772	1.343	0.942	1.132	1.036	0.972	0.803	0.732
MMP2-R-V	1.475	1.278	1.898	0.962	1.383	1.257	0.884	1.045	1.092	0.775	0.844	0.914	1.842	1.137	1.931	1.590	0.966	1.027	1.006	0.947	0.707	0.615
PAK4-R-V	1.630	1.353	2.044	1.074	1.315	1.356	0.973	1.393	1.195	0.644	0.870	0.896	1.960	1.417	1.839	1.563	0.907	0.931	1.101	0.964	0.708	0.683
JNK2-R-C	1.520	1.379	1.291	1.198	1.067	1.343	0.716	0.843	0.897	0.730	0.878	0.949	1.544	0.923	1.475	1.363	0.929	1.117	1.058	1.063	0.750	0.623
NF-kB-p65_pS536-R-C	1.010	1.029	1.300	1.162	1.135	1.154	0.703	0.851	1.041	0.704	0.752	0.946	1.264	1.149	1.279	1.410	0.940	1.162	0.983	0.904	0.684	0.703
Myosin-IIa-R-C	1.039	1.010	0.868	0.843	1.491	1.095	0.920	0.842	0.929	0.813	0.933	1.123	1.146	0.882	1.232	1.156	1.020	1.013	1.076	0.895	0.900	0.825
Ets-1-R-V	1.341	1.079	1.429	0.975	1.336	1.188	0.939	1.017	0.972	0.789	0.941	1.034	1.676	1.017	1.580	1.562	0.938	1.056	0.984	0.901	0.777	0.685
Lck-R-V	1.422	1.332	1.471	0.903	1.024	1.140	1.227	1.173	1.547	0.864	0.768	1.014	1.678	1.112	1.673	1.253	0.970	0.853	0.997	0.947	0.860	0.884
Connexin-43-R-C	0.550	0.425	1.068	4.713	4.759	3.614	0.343	0.618	0.865	0.314	3.650	3.155	0.610	0.867	0.483	6.098	3.282	5.190	0.898	1.042	0.489	0.931
ASNS-R-V	1.199	1.030	1.388	1.127	1.104	1.342	1.056	0.872	0.852	1.090	1.022	0.955	1.184	0.943	1.432	1.255	0.872	1.341	1.031	1.016	0.885	0.781
mTOR_pS2448-R-C	1.326	1.097	1.420	1.149	0.847	1.128	0.921	1.292	1.006	0.720	1.234	1.067	1.387	1.099	1.396	1.202	0.907	1.078	0.980	0.990	0.948	0.829
Axl-R-V	0.898	0.840	1.032	1.330	1.233	1.395	1.167	0.570	0.509	1.953	1.041	1.265	1.103	0.685	0.858	1.329	0.883	0.972	1.243	1.047	1.338	0.848
PEA-15-R-V	1.389	1.332	1.488	0.939	1.100	1.017	0.919	1.221	1.167	0.608	0.992	1.012	1.608	1.162	1.515	1.051	1.045	0.992	0.989	0.982	0.846	0.772
Claudin-7-R-V	1.286	1.411	2.157	0.921	0.848	1.054	1.071	1.177	1.701	0.842	0.782	0.887	2.177	1.229	1.444	1.009	0.967	0.979	0.955	1.001	0.850	0.814
ER-a_pS118-R-V	1.309	1.172	2.041	1.328	0.811	1.252	0.713	1.410	1.222	0.536	0.771	0.722	1.200	2.336	0.870	0.876	1.523	1.018	0.992	1.026	0.593	0.759
Merlin-R-C	1.524	1.278	1.263	1.079	0.980	1.123	0.980	0.879	0.970	1.030	0.836	0.877	1.681	0.978	1.612	1.203	1.026	0.941	1.004	0.952	0.865	0.662
eEF2K-R-V	1.645	1.360	1.443	0.990	1.143	1.122	0.841	0.895	0.896	0.830	0.708	0.765	1.827	0.830	1.777	1.481	0.931	1.031	0.945	1.003	0.669	0.594
Histone-H3-R-V	1.065	0.885	1.316	0.768	0.879	0.966	0.869	1.092	1.185	0.655	0.887	0.857	1.075	1.658	1.148	0.981	0.935	0.867	0.938	0.841	0.862	0.810
E-Cadherin-R-V	0.899	1.376	1.412	1.019	0.560	0.896	0.890	1.142	2.222	0.944	0.630	0.721	1.299	1.758	1.432	0.910	1.129	0.911	0.839	0.822	0.778	0.948
Chk1_pS345-R-C	1.519	1.472	1.831	1.042	1.115	1.123	0.734	1.265	1.137	0.699	0.954	0.952	1.793	1.155	1.681	1.184	0.921	1.099	1.024	1.042	0.813	0.677
Rad23A-R-C	1.220	1.261	1.089	0.918	1.299	0.972	0.737	1.118	1.139	0.464	1.007	1.088	1.632	1.157	1.491	1.122	1.000	1.004	1.020	0.834	0.819	0.699
HER3_pY1289-R-C	1.321	1.244	1.565	0.967	0.953	1.154	0.772	1.073	1.105	0.742	0.881	0.888	1.364	1.217	1.475	1.117	0.957	0.923	1.025	1.018	0.838	0.727
XPF-R-C	1.298	1.321	1.659	1.047	1.210	1.031	0.915	1.139	1.093	0.730	0.922	0.895	1.439	1.198	1.391	1.226	0.902	0.984	1.057	1.063	0.818	0.782
eIF4G-R-C	0.995	1.116	1.661	0.939	0.987	0.925	0.601	1.277	1.187	0.506	0.938	0.876	0.928	1.646	1.112	0.767	0.930	0.926	1.087	1.023	0.884	0.831
Akt1_pS473-R-V	1.833	0.842	1.368	1.679	0.740	0.937	0.973	1.064	0.782	1.237	1.259	1.019	0.872	0.746	1.349	0.924	0.928	1.036	1.162	1.363	1.217	0.950
CREB_pS133-R-C	1.084	0.909	0.915	1.160	0.918	0.745	0.532	1.430	1.145	0.484	1.302	1.060	1.109	1.507	0.893	0.846	0.935	0.886	1.059	0.829	1.068	0.885
P-Cadherin-R-C	1.166	1.023	1.230	0.965	1.043	1.035	0.942	0.936	0.945	1.073	0.753	0.812	1.267	0.977	1.287	1.181	1.012	0.882	0.990	0.968	0.858	0.800
TIGAR-R-V	1.376	1.253	1.384	1.035	1.218	1.153	0.856	1.050	1.129	0.676	0.975	1.007	1.507	1.111	1.604	1.385	0.955	1.088	0.994	0.951	0.776	0.719
UBAC1-R-V	1.093	0.924	1.111	0.859	1.162	1.053	0.817	0.803	0.760	1.012	0.799	1.039	1.232	0.822	1.290	1.256	0.938	1.024	0.956	0.935	0.886	0.712
PHLPP-R-V	1.189	1.204	1.331	1.010	1.132	1.104	0.808	0.973	0.849	0.727	1.252	1.027	1.319	1.015	1.406	1.230	0.887	1.037	1.029	0.996	0.953	0.703
TTF1-R-V	1.215	1.059	1.439	0.922	1.368	1.163	1.009	0.972	1.019	1.056	0.847	0.904	1.463	1.024	1.484	1.492	0.958	0.941	1.018	0.935	0.828	0.755
PKC-b-II_pS660-R-V	1.234	1.026	1.019	0.987	0.672	0.856	0.815	1.151	1.330	0.660	1.316	0.935	1.088	1.135	1.082	0.990	0.879	0.898	0.910	0.992	1.052	0.997
Hexokinase-II-R-V	0.511	0.875	0.849	0.860	1.623	0.886	1.201	0.849	1.198	1.712	1.549	1.310	0.614	1.122	0.880	0.882	0.762	1.345	1.127	0.854	1.529	1.242
HLA-DR-DP-DQ-DX-R-C	1.170	0.967	1.093	0.958	1.086	1.059	1.026	0.962	0.955	1.007	0.997	1.030	1.348	0.982	1.251	1.221	0.952	1.008	0.975	0.902	0.954	0.822
Stathmin-1-R-V	1.433	1.270	1.654	0.873	0.968	0.951	0.866	1.121	1.142	0.721	0.905	0.906	1.589	1.188	1.589	0.903	0.970	0.950	0.989	0.998	0.897	0.717
ARID1A-R-C	1.073	1.161	0.934	1.054	1.161	0.792	0.635	1.185	1.178	0.328	0.895	0.770	0.983	1.204	1.110	0.895	0.920	1.051	1.049	0.961	0.695	0.910
ERCC5-R-C	1.137	1.049	1.055	0.967	1.109	1.098	0.824	0.872	0.874	0.933	0.994	1.133	1.117	0.866	1.090	1.125	0.922	0.940	1.062	1.054	1.024	0.836
ULK1_pS757-R-C	1.054	0.841	0.852	1.327	1.177	1.120	1.203	1.124	0.706	1.368	1.649	1.069	0.794	0.657	0.789	1.118	1.007	1.185	1.095	1.226	1.234	1.354
YB1_pS102-R-V	1.146	1.103	1.309	0.987	1.068	0.999	1.015	1.112	1.103	0.883	0.924	1.025	1.132	1.201	1.215	1.045	0.921	1.043	1.015	1.003	0.941	0.910
MCT4-R-V	0.823	0.856	0.908	1.023	0.992	1.035	0.723	0.738	0.756	0.791	1.267	1.477	0.887	0.851	0.874	1.060	1.001	1.037	0.984	0.991	1.141	0.849
Smad3-R-V	1.278	1.204	1.253	0.994	1.203	1.100	0.857	1.149	1.072	0.608	0.874	0.924	1.309	1.144	1.289	1.104	1.007	1.050	1.043	0.998	0.761	0.822
CSK-R-C	1.312	1.165	1.119	0.950	1.034	1.053	1.151	1.123	1.087	1.024	1.083	1.067	1.413	1.042	1.369	1.160	0.942	1.005	0.977	0.940	1.022	0.879
Puma-R-C	1.220	1.175	1.266	0.991	1.163	0.922	0.964	1.040	0.992	0.947	1.049	1.025	1.256	1.011	1.231	1.084	0.959	1.000	1.011	1.047	0.993	0.857
FGF-basic-R-C	0.975	1.100	0.941	1.087	1.174	1.166	0.594	0.693	0.590	0.442	0.799	0.762	1.200	0.840	1.105	1.026	1.104	1.019	1.088	0.959	0.636	0.597
Hif-1-alpha-R-C	0.822	0.836	1.622	0.950	0.893	0.973	0.923	0.978	0.974	1.111	1.036	1.088	0.854	1.107	0.888	0.860	0.981	0.946	1.010	1.152	1.161	1.009
CDK1_pT14-R-C	0.584	0.856	1.011	0.916	1.401	1.073	0.691	0.758	0.852	0.682	1.169	1.063	0.598	1.353	0.949	1.231	1.057	1.141	0.989	0.846	0.850	0.794
Akt-R-V	1.398	1.515	1.296	1.053	1.113	1.236	0.782	1.048	1.043	0.492	1.013	0.895	1.529	1.023	1.384	1.084	1.022	1.067	1.072	1.069	0.756	0.730
PRAS40_pT246-R-V	1.514	1.182	1.704	1.225	1.198	1.239	0.887	1.175	1.012	0.865	1.033	0.953	1.452	1.114	1.619	1.480	0.972	1.008	1.058	1.052	0.824	0.735
LAD1-R-V	1.886	1.241	1.352	1.015	1.108	1.056	1.010	1.284	1.392	0.816	0.894	0.932	1.752	1.276	1.304	0.972	0.730	0.860	1.241	1.034	1.031	0.851
Tuberin_pT1462-R-V	1.416	1.289	1.504	1.100	1.092	1.101	0.929	1.090	1.110	0.799	0.938	0.949	1.497	1.142	1.511	1.152	0.936	1.036	1.054	1.014	0.860	0.754
Heregulin-R-V	0.914	1.176	1.231	0.931	0.988	0.858	0.835	1.034	1.243	0.734	0.889	0.947	0.855	1.346	0.939	0.920	0.911	0.944	1.001	1.058	0.926	0.991
SDHA-R-V	0.933	0.883	0.790	1.217	0.994	1.221	0.771	0.710	0.756	0.957	1.098	1.037	1.000	0.695	0.983	1.372	0.999	1.151	0.974	0.973	0.878	0.835
PYGB-R-V	1.408	1.211	1.360	0.973	1.055	1.106	1.087	1.132	1.102	0.991	1.018	0.996	1.655	1.029	1.494	1.244	0.986	1.004	0.969	0.953	0.929	0.795
DNA_POLG-R-V	0.955	0.627	0.783	4.248	1.327	1.887	0.830	0.650	0.617	1.094	1.097	1.763	0.860	0.596	0.852	4.005	0.956	1.444	1.165	1.024	0.617	0.908
B-Raf-R-C	1.299	1.315	1.305	1.086	0.852	1.082	0.576	1.105	1.036	0.488	1.127	0.955	1.394	1.095	1.384	1.117	0.972	1.071	0.956	1.012	0.814	0.701
MSH2-R-C	0.892	0.961	0.860	0.934	1.088	1.054	0.659	0.795	0.842	0.532	1.086	0.967	1.046	0.881	1.067	1.304	0.934	1.090	0.924	0.906	0.777	0.767
Stat3-R-C	1.621	1.552	2.210	0.844	0.878	1.011	0.873	1.494	1.325	0.564	0.911	1.079	2.176	1.300	1.680	0.737	0.914	0.765	1.132	1.044	1.057	0.716
HES1-R-V	0.844	0.876	0.816	1.061	1.256	1.000	0.844	0.845	1.041	0.858	1.293	1.177	0.889	1.054	0.779	0.945	1.067	1.092	1.069	0.932	1.072	1.003
Akt2-R-V	1.738	1.566	1.431	1.224	1.035	1.301	1.231	1.120	1.168	1.060	0.940	1.003	1.701	1.065	1.599	1.200	1.000	1.033	1.101	1.085	0.929	0.806
TRIP13-R-V	0.856	0.792	0.883	1.211	1.274	1.326	0.961	0.594	0.630	1.237	1.040	1.051	0.884	0.634	0.889	1.405	0.983	1.075	1.100	1.052	0.961	0.908
ER-a-R-V	1.247	1.219	1.204	1.018	0.693	0.871	1.157	1.033	1.081	1.339	0.802	0.803	1.462	1.115	1.355	0.973	1.116	0.843	0.880	0.933	1.004	0.832
Lasu1-R-V	1.000	0.932	0.969	0.907	1.039	0.986	0.806	0.852	0.835	0.887	1.010	1.031	1.031	0.878	1.051	1.035	0.942	1.019	0.979	0.980	0.977	0.842
DVL3-R-V	1.391	1.341	1.297	0.862	1.078	0.861	0.655	1.359	1.132	0.408	0.863	0.771	1.332	1.242	1.387	0.917	0.967	0.998	0.972	1.017	0.708	0.794
Sox2-R-V	1.037	0.901	1.278	0.882	1.036	0.958	0.956	0.824	0.920	1.051	0.931	0.840	1.088	0.874	1.277	1.079	0.983	0.847	0.989	0.993	0.970	0.833
AMPKa_pT172-R-C	0.946	1.262	1.076	1.031	1.119	1.187	0.578	1.148	1.062	0.416	1.037	0.915	1.281	1.099	1.118	0.909	1.114	1.094	1.071	0.939	0.760	0.797
Creb-R-C	0.889	0.848	0.860	1.299	2.066	1.569	0.703	0.769	0.815	0.733	0.906	0.949	0.868	0.818	0.940	1.616	1.079	1.316	1.230	0.989	0.645	0.871
PERK-R-V	1.003	1.063	1.228	1.006	1.137	1.144	0.836	1.078	1.063	0.582	0.997	0.967	0.849	1.064	1.025	1.017	1.016	1.039	1.070	1.121	0.829	1.013
Slfn11-G-C	1.551	1.288	0.725	1.035	1.225	1.065	0.979	1.222	1.252	0.783	0.872	0.993	1.834	1.130	1.729	1.499	0.924	1.094	0.945	0.760	0.753	0.736
GCLM-R-C	1.158	1.077	1.219	0.911	0.955	0.990	1.025	1.048	1.070	0.961	0.915	0.916	1.329	1.113	1.343	1.130	1.005	0.932	0.931	0.913	0.910	0.830
4E-BP1_pS65-R-V	1.149	1.049	1.269	0.987	1.005	0.989	0.673	0.935	0.946	0.644	0.977	0.863	1.078	1.059	1.065	1.126	0.973	0.998	0.962	1.083	0.802	0.798
PKA-a-R-V	1.025	1.000	1.221	0.867	1.183	0.958	0.778	1.181	1.011	0.539	1.153	1.354	0.924	1.048	0.923	0.824	0.997	0.945	1.088	1.121	1.101	1.026
eIF4E_pS209-R-V	1.092	0.987	1.098	0.915	0.994	0.967	0.997	0.997	1.029	1.061	0.901	0.920	1.106	1.045	1.151	1.025	1.003	0.904	0.981	0.962	0.983	0.915
WIPI2-R-C	0.835	1.224	0.761	1.240	1.144	0.560	1.234	1.020	0.823	1.571	1.112	1.166	0.861	0.843	0.845	1.163	1.216	1.087	0.850	1.106	1.111	1.207
Notch1-R-V	1.149	1.074	1.587	0.987	0.883	0.945	0.901	1.225	1.169	0.766	0.833	0.848	1.028	1.437	1.131	0.859	1.026	0.938	0.997	1.060	0.867	0.916
PDGFRB-R-V	1.843	1.263	1.031	1.180	1.112	0.847	1.140	1.336	1.152	0.636	0.793	0.658	1.613	1.066	1.092	1.048	1.096	1.116	0.963	1.097	0.640	0.962
mTOR-R-V	1.172	1.121	1.058	0.998	0.786	1.137	0.759	0.950	0.933	0.544	1.079	1.017	1.282	0.924	1.197	1.107	1.081	1.095	0.890	0.985	0.804	0.776
TUFM-R-V	1.705	1.382	1.665	1.004	0.867	1.000	1.083	1.260	0.983	1.152	0.790	0.848	1.906	0.986	1.651	1.122	0.908	0.867	0.991	1.046	0.963	0.732
Gli3-R-C	1.027	0.855	0.985	1.022	1.055	1.028	1.102	0.835	0.793	1.181	0.984	0.969	1.088	0.815	1.068	1.139	0.914	0.961	1.030	0.965	1.040	0.919
SLC1A5-R-C	1.173	0.879	1.142	1.111	0.844	1.195	1.227	0.852	0.916	1.500	0.710	0.933	1.277	0.858	1.052	0.954	1.032	0.685	1.180	1.002	1.177	0.940
4E-BP1_pT37_T46-R-V	1.797	1.200	1.447	1.287	1.045	0.850	1.337	1.539	0.881	1.289	0.982	0.737	1.387	0.907	1.246	1.025	0.826	0.980	1.124	1.255	1.062	1.061
Lyn-R-V	0.951	1.006	0.904	0.910	0.795	0.945	1.148	0.973	1.236	1.051	0.840	1.000	0.967	0.946	0.983	0.910	0.938	0.896	0.965	0.988	1.053	1.159
c-Jun_pS73-R-V	0.950	0.943	1.070	0.957	0.996	0.969	0.944	1.181	0.926	1.138	1.079	1.051	1.097	0.841	0.938	0.830	0.817	0.877	1.158	1.030	1.295	1.061
MERIT40_pS29-R-V	1.049	0.962	1.089	0.943	0.998	0.962	0.969	0.847	0.945	1.022	0.889	0.897	1.165	1.019	1.202	1.108	1.078	0.862	0.953	0.915	0.921	0.815
Fibronectin-R-V	2.287	1.855	2.648	0.848	0.435	0.889	1.502	2.048	1.777	0.625	0.457	0.534	1.147	1.734	1.688	0.381	0.563	0.553	1.451	1.486	1.080	1.166
Caveolin-1-R-V	2.251	2.572	1.509	1.472	0.783	1.188	1.189	1.414	1.439	1.015	0.432	0.484	2.365	1.147	2.152	0.941	0.942	0.935	1.222	1.118	0.685	0.714
NDRG1_pT346-R-V	1.856	1.206	1.575	1.322	1.050	0.891	1.555	1.202	0.907	1.469	0.758	0.764	1.170	0.769	1.414	0.931	0.800	0.902	1.240	1.383	1.136	1.093
RBM15-R-V	1.185	1.085	1.230	0.968	1.452	0.923	0.560	0.964	1.046	0.397	1.059	0.834	1.323	1.039	1.251	0.790	0.987	1.038	1.187	0.969	0.813	0.712
VASP-R-V	1.284	1.403	1.723	0.965	1.149	1.074	1.051	1.086	1.256	0.862	0.715	0.800	1.080	1.165	1.387	1.054	0.859	1.032	1.083	1.215	0.807	0.935
53BP1-R-V	1.212	1.077	0.935	0.991	1.265	0.957	0.654	0.929	0.978	0.416	1.386	1.087	1.269	0.903	1.194	1.075	0.925	0.996	1.073	0.958	0.965	0.761
Src_pY527-R-V	1.051	1.044	0.836	1.314	0.999	1.060	0.729	0.994	0.922	0.484	1.260	0.956	1.013	0.730	0.915	1.054	0.698	1.051	1.203	1.103	0.963	0.995
TAZ-R-V	1.130	1.062	1.304	0.997	1.204	1.060	0.930	0.775	0.851	1.015	0.864	1.081	1.216	0.804	1.236	1.165	0.958	0.988	1.048	1.074	0.951	0.785
Mcl-1-R-V	0.752	0.870	0.925	0.970	1.373	1.107	0.827	0.916	0.833	0.644	1.030	1.161	0.810	0.894	0.899	0.970	1.032	1.144	1.097	0.978	0.901	0.989
EGFR_pY1173-R-V	1.314	1.210	1.380	0.928	0.987	0.990	0.919	1.143	1.111	0.848	0.881	0.923	1.403	1.149	1.430	1.088	0.959	0.941	0.972	0.981	0.887	0.797
B-Raf_pS445-R-V	1.316	1.205	1.231	0.995	0.764	1.145	0.768	1.168	1.076	0.500	1.161	0.959	1.473	1.140	1.336	0.971	1.025	1.054	0.952	0.950	0.859	0.763
GCN5L2-R-V	1.116	1.188	0.827	0.997	1.049	1.014	0.843	0.974	0.954	0.729	1.105	0.953	1.183	1.008	1.178	1.078	1.014	1.063	0.970	0.929	0.883	0.823
Smad1-R-V	1.304	1.312	1.111	0.946	0.915	0.860	0.856	1.262	1.190	0.599	0.803	0.842	1.346	1.110	1.176	0.924	0.911	1.108	0.925	1.026	0.763	0.911
PLC-gamma2_pY759-R-V	1.051	0.829	1.014	0.981	0.897	0.904	1.055	0.916	0.848	1.215	0.999	0.965	1.156	0.847	1.014	0.892	0.961	0.923	1.003	0.959	1.146	0.935
DNMT1-R-V	0.641	0.813	0.885	1.161	1.221	1.226	0.606	0.851	0.814	0.476	1.141	0.854	0.654	1.220	0.907	1.478	1.212	1.458	0.870	0.841	0.596	0.817
Bax-R-V	1.093	1.146	1.064	0.957	1.276	0.976	0.786	0.957	0.990	0.729	0.882	0.857	1.153	1.012	1.189	1.045	1.063	1.006	1.030	0.985	0.793	0.815
c-Abl-R-V	1.270	1.243	1.359	0.952	1.083	1.056	0.978	1.225	1.082	0.825	1.020	0.978	1.418	1.088	1.311	1.082	0.970	1.012	1.009	1.014	0.921	0.861
Oct-4-R-C	1.182	1.155	1.557	0.924	1.033	0.985	0.944	1.122	1.084	0.791	0.888	0.904	1.296	1.309	1.345	1.070	0.925	0.922	1.009	0.986	0.886	0.797
CD26-R-V	0.957	0.915	0.958	0.928	1.035	1.055	0.923	0.830	0.788	1.048	0.901	0.981	1.062	0.802	0.995	1.065	0.941	0.952	1.020	0.990	0.991	0.889
c-IAP2-R-C	1.266	1.223	1.242	0.989	1.200	1.018	1.006	1.289	1.094	0.621	1.097	0.989	1.259	1.200	1.228	1.099	1.035	1.001	1.023	1.012	0.864	0.919
PDK1-R-V	1.079	0.997	1.171	1.020	1.083	1.044	0.882	0.896	0.920	0.881	0.909	0.844	1.149	0.951	1.146	1.079	1.041	0.933	1.030	1.000	0.862	0.831
Grp75-R-C	0.687	0.650	0.566	1.252	1.384	1.045	1.057	0.659	0.624	1.394	1.359	1.222	0.794	0.716	0.704	1.434	1.248	1.298	0.925	0.859	0.999	1.057
HSP70-R-C	1.311	1.285	1.565	1.014	1.076	1.096	1.063	1.192	1.173	0.921	0.777	0.897	1.432	1.091	1.406	1.181	1.013	0.964	1.009	1.059	0.822	0.872
PTPN12-R-V	1.100	1.068	1.031	1.012	0.944	0.885	0.904	1.001	1.054	1.010	0.900	0.989	1.042	1.069	1.103	0.913	0.996	0.955	0.992	0.996	1.013	0.921
Rad51-R-C	0.684	0.892	1.069	0.960	1.067	1.057	0.873	0.929	0.856	0.825	0.898	0.822	0.597	1.188	1.054	1.175	0.946	1.102	0.956	0.932	0.789	0.936
BCL2A1-R-V	1.112	1.106	1.164	1.051	1.054	1.037	1.092	1.081	1.048	0.965	1.064	1.009	1.149	1.093	1.216	1.018	0.953	1.006	1.055	0.978	1.020	0.931
CD44-R-C	0.874	0.835	0.863	0.966	0.930	0.898	1.341	0.826	0.816	1.454	1.110	1.105	0.793	0.943	0.884	0.904	0.962	1.029	0.965	0.981	1.268	1.138
FAK-R-C	1.329	1.257	1.393	0.904	0.992	1.028	0.999	1.022	1.169	1.004	0.794	0.890	1.365	1.122	1.394	1.033	0.997	0.951	0.981	1.025	0.902	0.822
Src_pY416-R-V	1.101	1.082	1.197	1.027	1.077	1.067	0.900	1.193	1.067	0.832	1.027	0.992	1.101	1.160	1.042	0.982	1.033	0.948	1.070	1.023	0.963	0.957
C-Raf-R-C	1.056	1.041	1.144	0.957	0.815	0.962	0.774	1.007	1.027	0.605	0.994	0.880	1.033	1.128	1.061	0.933	0.974	1.064	0.920	1.005	0.834	0.871
c-Met_pY1234_Y1235-R-V	1.167	1.156	1.194	0.866	0.986	0.918	0.783	1.120	1.101	0.621	0.885	0.899	1.120	1.246	1.147	0.883	0.982	0.893	1.005	1.001	0.872	0.855
Jagged1-R-V	1.157	1.057	1.067	0.904	1.009	1.042	0.981	1.014	0.980	0.931	0.970	1.035	1.174	0.947	1.149	0.992	0.945	1.016	1.001	1.004	0.994	0.910
RSK-R-C	0.892	1.026	0.948	0.961	0.888	0.958	1.046	1.021	1.447	0.816	1.025	0.980	0.999	1.142	0.957	0.921	0.981	1.045	0.952	0.925	0.957	1.134
Elk1_pS383-R-C	1.153	1.103	1.294	0.988	0.928	0.998	0.961	1.044	1.018	0.903	0.923	0.896	1.156	1.148	1.233	1.029	0.993	0.947	0.982	1.004	0.917	0.855
EGFR-R-V	1.173	1.186	1.185	0.948	1.049	0.972	0.959	1.168	1.055	0.823	0.901	0.965	1.182	1.149	1.281	0.986	0.944	0.938	1.035	0.982	0.938	0.881
PAICS-R-C	0.903	0.886	0.898	0.859	0.957	0.978	0.804	0.907	0.847	0.644	1.208	1.103	0.979	0.871	1.026	1.277	0.994	1.023	0.848	0.934	0.897	0.889
COG3-R-V	0.908	0.956	0.954	0.955	1.086	0.960	0.844	0.932	0.916	0.940	1.022	1.088	0.839	0.931	0.872	0.926	0.969	0.989	1.041	1.066	1.058	1.019
ERRalpha-R-V	0.886	0.805	0.930	0.953	1.101	0.962	0.949	0.947	1.137	1.073	1.138	1.108	0.946	1.124	0.897	0.811	1.068	0.968	1.060	0.884	1.166	1.023
JNK_pT183_Y185-R-V	1.157	1.138	1.094	1.023	1.172	1.084	0.993	1.149	1.052	0.801	1.072	1.005	1.123	1.102	1.129	1.171	1.051	1.087	0.991	1.010	0.870	0.952

FAK_pY397-R-V	0.866	0.912	1.188	1.301	0.885	1.107	0.978	1.379	1.050	1.144	1.082	1.072	0.959	1.162	0.724	0.901	1.056	1.130	1.067	1.043	1.068	1.198
Gab2-R-V	0.854	0.881	0.967	1.383	1.731	1.215	0.456	0.909	0.948	0.372	1.462	1.415	0.777	0.763	0.713	1.304	1.307	1.304	1.105	1.199	0.830	1.027
Atg7-R-V	1.176	1.208	0.952	1.047	0.875	1.149	1.217	1.072	1.068	1.029	1.086	1.094	1.141	0.977	1.092	0.968	1.015	1.014	1.025	1.040	1.071	1.046
BRD4-R-V	1.001	1.045	1.060	0.945	1.082	1.137	0.946	0.941	0.853	0.942	1.000	1.067	1.037	0.978	1.037	1.030	0.942	0.953	1.082	1.018	1.028	0.898
DDR1_pY513-R-C	1.022	0.978	1.059	0.915	1.021	0.997	0.813	1.008	0.978	0.865	1.003	1.039	1.025	1.034	1.023	1.004	0.966	0.985	0.993	0.993	0.984	0.908
MMP14-R-V	1.504	2.198	1.363	0.825	1.196	1.011	0.856	1.553	1.774	0.554	0.895	0.880	1.212	1.557	1.296	0.591	0.733	0.775	1.445	1.246	1.109	1.029
LC3A-B-R-C	0.873	0.904	0.930	0.966	1.152	1.117	0.774	0.877	0.712	0.739	1.492	0.821	0.869	0.845	0.782	1.034	1.107	1.197	0.969	1.084	0.914	0.946
Jak2-R-V	1.331	1.449	1.116	0.899	0.714	0.921	1.096	1.186	1.634	0.753	0.897	0.952	1.525	1.328	1.505	0.889	0.881	0.865	0.962	0.894	0.988	0.899
p27-Kip1-R-V	1.091	1.075	1.155	0.915	1.192	1.017	0.817	0.999	1.000	0.644	0.972	1.037	1.040	1.078	1.067	0.964	0.960	0.999	1.069	1.043	0.907	0.884
Cyclin-E1-R-V	0.891	0.846	0.800	1.009	1.020	1.051	0.926	0.851	0.830	0.915	1.053	1.157	0.889	0.850	0.933	0.980	0.986	1.085	1.009	0.949	1.024	0.975
Akt2_pS474-R-C	2.708	0.749	1.245	1.842	0.789	0.895	1.476	1.192	0.932	1.464	1.187	0.953	0.650	0.683	1.006	0.962	0.974	1.059	1.178	2.010	1.203	1.539
DRP1-R-V	0.859	0.898	0.895	0.902	1.097	0.934	0.636	1.010	0.858	0.462	1.269	0.987	0.887	0.943	0.860	0.974	1.039	1.053	0.956	0.985	0.887	0.930
p27_pT157-R-C	1.216	1.170	1.210	0.987	1.167	1.025	0.978	1.213	1.086	0.859	1.000	0.996	1.287	1.103	1.214	1.084	1.064	1.014	1.005	0.998	0.903	0.909
AMPKa-R-C	1.210	1.144	1.386	0.922	0.962	1.030	0.834	1.114	1.139	0.662	0.888	0.850	1.381	1.131	1.249	0.946	0.998	0.971	0.999	0.995	0.823	0.821
Pyk2_pY402-R-C	0.904	0.855	0.893	1.262	0.926	1.048	1.070	1.016	0.866	1.230	1.207	1.071	0.816	1.082	0.841	0.880	1.014	1.045	1.101	0.968	1.194	1.077
SGK1-R-V	0.883	0.792	0.866	2.070	1.852	2.255	0.918	0.778	0.828	0.941	1.093	1.170	0.843	0.863	0.833	1.671	1.796	2.809	0.984	1.000	0.511	0.994
Bad_pS112-R-V	0.985	1.030	1.077	1.013	1.027	0.998	0.838	1.033	1.030	0.815	1.111	1.028	1.017	1.077	1.044	0.986	0.957	1.003	1.032	0.985	1.003	0.924
PI3K-p110-a-R-C	1.361	1.432	1.144	0.957	0.912	1.011	0.938	1.264	1.142	0.665	0.946	1.024	1.193	1.148	1.199	0.901	0.956	1.047	0.992	1.112	0.907	0.945
Gys_pS641-R-V	1.444	1.018	1.736	1.307	0.792	1.014	1.017	1.220	1.069	1.365	0.943	0.807	1.385	1.163	1.310	1.256	0.929	0.947	0.994	1.088	0.994	0.857
AMPK-a2_pS345-R-V	1.042	1.033	1.058	0.911	1.113	1.006	0.913	1.131	1.105	0.770	1.084	1.054	1.023	1.153	1.029	0.952	1.024	0.995	1.020	0.978	0.979	0.983
Cox2-R-C	1.126	1.107	1.256	0.953	1.046	0.972	0.959	1.166	1.084	0.840	1.002	1.004	1.279	1.141	1.205	1.197	1.011	1.001	0.926	0.963	0.887	0.885
ACVRL1-R-C	1.290	1.233	1.273	0.987	1.014	1.020	1.008	1.188	1.032	0.894	0.832	0.841	1.372	1.052	1.301	1.240	1.013	0.994	0.931	1.019	0.791	0.867
HMHA1-R-V	1.225	1.115	1.357	0.806	0.706	0.856	1.323	0.947	0.937	1.346	0.649	0.766	1.514	0.869	1.308	0.941	0.919	0.678	0.933	1.002	1.088	0.869
SFRP1-R-C	1.043	1.030	1.093	1.016	0.980	1.028	1.022	1.002	0.938	0.987	0.844	1.009	0.909	0.975	1.062	0.990	0.992	0.943	1.034	1.075	0.971	1.006
VEGFR-2-R-V	1.176	1.226	1.439	0.836	0.935	0.966	0.843	1.307	1.172	0.674	0.933	0.869	1.281	1.157	1.237	0.825	1.048	0.856	1.003	1.045	0.907	0.904
LRP6_pS1490-R-V	1.002	1.010	1.000	1.014	1.031	0.963	0.944	1.029	0.931	0.865	1.023	1.009	0.949	0.939	0.963	0.950	0.952	1.018	1.030	1.056	0.992	1.018
CD86-R-C	1.097	1.026	1.073	0.939	0.938	0.949	1.073	0.957	1.039	1.034	0.942	1.031	1.145	1.087	1.168	0.979	1.004	0.940	0.966	0.940	1.028	0.903
Raptor-R-V	1.238	1.171	1.136	1.001	0.825	1.116	0.735	0.953	0.932	0.590	0.924	0.944	0.931	1.035	1.252	1.084	1.034	1.134	0.905	1.101	0.756	0.814
eIF4E-R-V	1.012	1.267	1.136	0.947	1.346	0.962	0.731	1.189	1.087	0.447	1.073	0.938	1.011	1.157	1.142	0.988	0.941	1.039	1.097	1.032	0.828	0.908
MRAP-R-C	0.956	0.847	0.882	0.909	0.878	0.892	0.994	0.905	0.831	1.075	0.931	0.947	0.954	0.829	0.912	0.880	0.963	0.907	0.974	0.996	1.074	1.013
Chk1_pS296-R-V	1.066	1.078	0.919	0.918	0.904	0.940	1.118	1.024	1.073	1.048	0.978	1.084	1.108	1.003	1.047	0.892	0.928	0.951	0.997	0.970	1.122	1.018
D-a-Tubulin-R-V	1.247	1.245	1.252	0.933	0.987	0.989	1.098	1.269	1.227	0.805	1.147	0.980	1.101	1.135	1.168	0.853	0.951	0.958	1.053	1.100	1.061	1.056
Aurora-ABC_pT288_pT232_pT198-R-C	0.802	0.798	0.916	1.145	1.053	1.087	1.122	0.842	0.886	1.421	1.104	1.138	0.773	0.899	0.794	1.014	1.094	1.113	1.020	1.021	1.137	1.156
NAPSIN-A-R-C	1.019	0.960	0.979	0.912	0.992	0.955	0.960	1.027	0.976	0.904	1.011	1.032	0.971	1.000	0.985	0.918	0.978	0.974	0.996	1.000	1.027	1.002
Tuberin-R-V	0.901	0.985	0.989	1.178	1.020	1.254	1.044	0.773	0.768	1.307	1.038	1.061	0.837	0.741	0.823	1.060	1.104	1.027	1.082	1.198	1.067	1.077
p70-S6K1-R-V	1.256	1.300	1.378	0.952	1.245	1.040	0.824	1.066	1.154	0.625	0.809	0.887	1.396	1.124	1.238	0.934	0.939	0.982	1.134	1.047	0.813	0.810
C-Raf_pS338-R-V	0.995	0.911	1.042	1.163	0.841	0.967	1.146	0.949	0.987	1.307	0.941	0.920	0.947	0.935	0.920	1.016	1.051	0.850	1.019	1.052	1.087	1.100
DM-Histone-H3-R-V	0.958	0.885	0.928	0.902	0.889	0.918	1.035	1.056	1.097	1.039	1.013	1.042	0.843	1.213	0.872	0.856	0.981	0.919	0.983	0.946	1.123	1.089
b-Catenin_pT41_S45-R-V	1.020	1.030	1.111	1.000	1.008	1.042	0.956	0.964	1.066	0.972	1.038	0.999	1.091	1.083	1.161	1.073	0.979	0.978	1.007	0.948	0.993	0.896
SHP-2_pY542-R-C	0.993	0.960	1.058	1.107	0.963	0.950	0.945	1.125	1.086	0.922	0.834	0.871	0.985	1.161	0.993	0.904	0.956	0.920	1.086	0.959	0.945	1.006
Mitofusin-2-R-V	1.165	1.190	1.316	1.028	0.951	0.961	1.126	1.372	1.147	0.984	1.082	1.002	1.444	1.139	1.127	1.030	1.171	0.969	0.927	0.989	0.968	0.982
ACC_pS79-R-V	0.885	0.847	1.288	1.251	0.715	1.013	2.059	0.771	1.159	3.779	0.865	0.978	0.963	1.054	1.168	1.021	0.807	1.175	0.992	0.949	1.872	1.253
Enolase-2-R-V	0.908	0.870	0.808	1.086	0.896	0.989	1.161	0.832	0.876	1.404	1.428	1.068	0.860	0.849	0.859	0.950	1.055	0.961	1.002	1.007	1.315	1.118
PKC-a-b-II_pT638_T641-R-V	1.346	1.029	0.861	1.113	0.854	1.064	1.107	0.983	0.895	1.367	1.035	1.066	1.166	0.853	1.099	0.938	1.030	1.021	1.014	1.038	1.160	0.957
Stat3_pY705-R-C	0.923	0.956	0.995	0.938	0.947	0.938	0.943	1.052	0.969	1.000	0.983	1.007	0.937	1.129	0.934	0.924	0.986	0.889	1.009	0.958	1.069	0.988
p44-42-MAPK-R-V	1.056	1.208	1.014	0.908	1.084	0.968	0.991	1.207	1.188	0.654	1.090	1.031	1.203	1.129	1.094	0.897	1.005	1.014	1.015	0.957	0.952	0.989
Atg4B-R-C	0.974	1.003	1.011	0.937	0.989	0.927	0.826	0.971	1.016	0.824	0.996	0.924	0.987	1.095	1.011	0.999	1.035	0.961	0.952	0.966	0.916	0.909
MEK2-R-V	0.914	0.897	0.901	0.954	0.971	0.940	1.033	0.984	0.938	1.011	1.095	1.078	0.868	0.984	0.881	0.944	1.004	0.963	0.984	0.993	1.093	1.081
p27_pT198-R-V	1.000	1.001	1.070	1.020	1.016	0.909	1.000	1.066	1.006	0.886	1.034	1.007	0.984	1.048	0.979	0.917	0.953	1.006	1.023	1.020	1.017	1.020
ATM-R-V	1.076	1.128	0.755	1.001	0.866	1.034	1.049	0.989	1.127	1.072	1.126	1.174	1.130	0.959	1.123	0.979	1.052	1.023	0.950	0.921	1.104	0.986
Cdc6-R-V	0.779	0.760	0.840	1.378	1.254	1.300	1.315	0.820	0.843	1.876	1.233	1.373	0.749	0.994	0.790	1.270	1.343	1.323	0.999	0.939	1.139	1.176
Bid-R-C	0.945	0.930	0.928	0.946	0.836	0.900	0.935	0.786	0.870	1.134	0.791	0.846	0.917	0.910	0.965	0.923	1.018	0.823	0.971	1.004	1.003	0.928
p90RSK_pT573-R-C	1.463	0.958	1.055	1.692	0.796	0.889	1.205	1.100	0.991	1.418	1.680	1.191	0.819	0.811	0.814	1.051	0.943	0.892	1.170	1.423	1.486	1.349
Rictor_pT1135-R-V	1.172	1.100	1.237	1.009	0.949	0.931	0.933	1.092	1.172	0.793	0.873	0.889	0.850	1.203	1.197	0.988	0.925	0.976	1.000	1.079	0.885	0.984
PD-L1-R-C	1.020	1.046	1.124	0.895	1.007	0.891	0.903	1.056	1.072	0.786	0.915	0.956	0.984	1.167	1.049	0.892	0.945	0.946	1.004	0.997	0.955	0.948
14-3-3-zeta-R-V	0.934	0.877	0.781	0.900	1.083	0.934	0.909	0.896	0.908	0.826	1.110	1.076	0.954	0.831	0.901	0.945	1.000	1.039	0.977	0.966	1.010	1.011
PAK1-R-V	0.998	1.120	0.850	0.941	1.017	0.959	1.100	1.055	1.231	1.063	1.596	0.859	1.078	1.049	1.087	0.741	1.053	0.879	1.091	0.923	1.316	1.053
ATRX-R-C	0.888	0.849	0.538	0.816	1.387	0.855	0.393	0.668	0.963	0.303	1.317	1.355	0.975	0.931	1.091	0.929	1.097	1.155	0.961	0.759	0.935	0.675
N-Cadherin-R-V	1.176	1.236	1.096	0.996	0.873	0.961	1.176	1.286	1.461	1.071	1.039	1.072	0.765	1.186	1.084	0.840	1.022	0.941	1.010	1.156	1.135	1.292
Wee1_pS642-R-C	1.060	1.046	1.087	0.986	0.938	0.926	1.004	1.119	1.001	1.012	0.980	0.930	1.052	1.097	1.089	0.974	1.002	0.941	0.977	0.986	1.002	0.964
IRS1-R-V	1.316	1.223	1.148	1.067	0.966	0.987	0.932	1.325	1.150	0.735	0.897	0.851	1.353	1.157	1.166	1.005	1.061	1.038	0.973	1.003	0.800	0.927
Hexokinase-I-R-C	0.917	0.894	0.921	0.902	0.891	0.952	0.917	0.914	0.836	1.091	1.057	1.073	0.917	0.826	0.868	0.873	0.987	0.955	0.975	1.046	1.144	1.021
MEK1_p_S217_S221-R-V	1.073	1.049	1.099	0.865	0.971	0.914	0.853	1.104	1.105	0.723	0.966	0.937	1.052	1.168	1.044	0.874	0.992	0.902	0.994	0.987	0.949	0.938
Atg3-R-V	0.913	0.908	0.882	0.882	0.978	0.947	0.944	0.871	0.876	1.068	1.008	1.048	0.888	0.914	0.872	0.898	0.945	0.945	1.007	1.011	1.121	1.006
Bcl2-R-V	0.896	0.853	0.937	0.906	0.885	0.958	1.140	0.887	0.820	1.249	0.888	0.993	0.890	0.909	0.913	0.910	0.968	0.874	0.999	0.990	1.137	1.050
KAP1-R-V	1.074	1.054	0.917	0.956	1.239	0.931	0.806	0.950	1.039	0.628	1.047	1.006	1.114	0.908	1.051	1.084	0.975	1.002	1.021	0.990	0.876	0.909
Glutamate-D1-2-R-V	1.105	1.178	1.004	0.930	1.160	0.976	0.963	1.064	1.072	0.721	1.282	1.066	1.157	0.963	1.143	0.979	0.943	0.932	1.074	1.007	1.075	0.950
FOXO3-R-V	1.290	1.366	0.884	1.126	1.127	0.925	0.675	1.240	1.118	0.434	0.983	0.953	1.249	0.966	1.176	1.015	1.027	1.104	1.010	1.044	0.753	0.895
ZEB1-R-V	1.398	1.187	1.121	1.057	1.169	0.998	0.935	1.392	1.056	0.747	1.014	0.986	1.196	1.170	1.224	0.955	0.987	1.027	1.086	1.032	0.925	0.942
YAP_pS127-R-V	1.575	1.567	1.377	1.273	1.054	0.954	1.333	1.475	1.268	0.807	0.959	0.928	1.295	1.084	1.477	0.992	0.812	1.044	1.152	1.172	0.946	1.057
DDR1-R-V	0.853	0.802	0.763	1.160	0.915	1.175	1.130	0.766	0.871	1.367	1.175	1.186	0.938	0.819	0.781	1.141	1.278	1.070	0.932	0.953	1.068	1.090
PI3K-p85-R-V	1.200	1.394	1.103	0.819	0.994	0.916	1.138	1.380	1.372	0.679	0.883	0.893	1.065	1.181	1.101	0.794	0.915	0.834	1.073	1.104	0.965	1.162
DJ1-R-V	1.059	1.064	0.933	0.922	1.049	0.938	0.963	1.150	1.004	0.635	1.011	1.056	1.085	0.959	1.021	0.975	0.992	1.006	0.979	0.997	0.909	1.017
B7-H4-R-C	1.134	1.328	1.488	0.892	0.819	0.842	1.086	1.324	1.300	1.054	0.893	0.877	1.096	1.281	1.095	0.886	1.026	0.870	0.917	1.138	1.015	1.069
SGK3-R-V	1.159	0.966	0.893	0.943	0.830	0.943	0.991	1.005	0.924	1.054	0.904	1.105	1.130	0.890	1.072	0.856	0.936	0.941	0.994	0.976	1.120	0.945
FoxO3a_pS318_S321-R-C	1.175	1.225	1.401	1.035	1.005	1.027	0.844	1.173	1.084	0.721	0.986	0.936	1.225	1.133	1.249	1.085	0.924	1.039	1.006	1.054	0.867	0.859
U-Histone-H2B-R-C	1.488	1.074	0.874	1.101	0.903	0.918	1.270	1.416	1.149	0.691	1.109	0.806	1.296	1.290	1.187	1.068	0.874	1.032	0.982	0.911	0.876	1.017
Rad50-R-V	0.952	0.937	0.816	1.035	1.091	1.027	0.904	0.780	0.745	1.163	1.074	1.078	1.010	0.714	0.881	1.086	1.035	0.988	1.014	1.038	1.066	0.933
RPA32-R-V	0.914	1.030	0.806	1.019	1.440	1.023	0.941	0.942	0.906	0.807	1.116	1.060	1.026	0.875	0.988	1.221	1.090	1.142	1.008	0.952	0.864	0.965
ATM_pS1981-R-V	0.999	0.969	1.048	0.987	0.947	0.987	1.063	1.064	0.998	1.003	1.027	1.035	0.983	1.058	0.997	0.930	0.969	1.003	1.007	0.993	1.056	1.029
PKC-delta_pS664-R-V	0.958	0.917	0.959	0.959	0.950	0.890	1.028	1.110	1.028	0.943	0.985	1.088	0.913	1.062	0.951	0.897	1.051	1.028	0.940	0.969	1.013	1.082
PRC1_pT481-R-C	1.176	0.968	0.965	1.036	0.988	0.933	1.032	1.096	0.983	1.016	1.104	1.015	1.108	0.983	0.984	1.017	0.969	0.958	1.005	1.011	1.065	1.012
MIF-R-C	0.947	0.905	0.895	0.968	0.952	0.916	0.959	0.969	0.873	1.031	0.982	0.996	0.895	0.930	0.902	0.946	1.014	0.982	0.964	1.007	1.023	1.027
Notch3-R-C	0.960	0.921	0.901	0.914	0.966	0.906	0.988	0.940	0.894	0.911	0.869	0.928	0.865	0.923	0.914	0.856	0.964	0.994	0.990	1.030	0.962	1.045
RRM1-R-C	0.952	0.890	0.969	0.935	0.880	0.920	1.017	0.876	0.940	1.067	0.931	0.933	0.905	0.986	0.980	0.968	1.016	0.953	0.931	0.979	0.998	0.987
STING-R-V	0.831	1.043	1.179	1.002	0.826	1.046	1.111	0.806	1.201	1.416	0.969	1.229	0.844	0.975	0.875	0.790	0.932	0.835	1.124	1.134	1.413	1.158
Beclin-R-C	0.999	1.029	1.007	0.921	0.982	0.916	0.989	1.044	1.042	0.973	1.026	1.050	0.984	1.065	0.951	0.930	0.958	1.002	0.975	1.011	1.055	1.025
MAPK_pT202_Y204-R-V	1.066	0.947	0.997	1.064	0.877	0.875	1.043	1.093	0.994	1.070	0.932	0.946	0.896	0.982	0.934	0.860	0.997	0.857	1.038	1.071	1.086	1.113
Patched-R-C	0.939	0.914	0.889	0.885	0.876	0.926	0.943	0.927	0.846	1.072	0.951	1.014	0.982	0.820	0.901	0.894	0.955	0.831	1.002	1.015	1.133	1.005
PAX8-R-C	0.887	1.038	0.943	0.851	0.663	0.888	0.990	1.083	1.470	0.808	0.836	0.825	1.137	1.286	1.001	0.781	0.989	0.842	0.920	0.837	0.946	1.034
Myt1-R-C	0.952	0.960	1.062	0.892	1.018	0.925	0.979	0.927	0.957	1.037	0.894	0.890	0.960	1.004	1.022	1.000	0.983	0.846	1.002	0.996	0.997	0.959
ATR_pS428-R-C	0.909	0.880	0.845	0.978	0.915	0.896	0.944	0.885	0.908	1.143	0.928	0.920	0.870	0.948	0.884	0.931	1.051	0.920	0.961	0.975	1.031	1.013
PMS2-R-V	0.801	0.808	0.715	1.339	1.136	1.248	1.326	0.849	0.841	2.008	1.247	1.328	0.803	0.803	0.809	1.208	1.363	1.128	1.006	0.963	1.239	1.249
Myosin-IIa_pS1943-R-V	0.903	0.758	0.442	1.356	1.000	0.810	1.622	0.746	0.922	2.090	1.117	1.170	0.886	0.928	0.901	1.280	1.449	1.074	0.832	0.774	1.151	1.212
p38-MAPK-_pT180_Y182-R-V	0.730	0.801	0.787	1.139	1.055	1.018	1.037	1.106	1.137	1.112	1.354	1.234	0.769	1.290	0.802	0.991	1.157	1.059	1.001	0.810	1.153	1.146
p70-S6K_pT389-R-V	1.106	1.024	1.079	1.001	0.857	0.948	1.031	1.058	0.996	1.093	0.907	0.887	1.035	0.984	1.078	1.044	1.086	1.002	0.896	1.036	0.921	0.996
DDB-1-R-V	1.047	0.989	0.854	1.051	0.881	0.936	0.877	0.955	1.047	0.923	1.090	1.025	0.973	1.071	1.000	0.945	1.049	1.085	0.932	0.950	0.987	0.946
PHGDH-R-C	0.987	0.831	0.769	1.098	1.051	1.059	1.106	0.818	0.855	1.190	1.124	1.042	0.848	0.782	0.914	1.020	0.990	1.043	1.051	1.017	1.099	1.093
p38-MAPK-R-V	0.991	1.102	0.961	0.777	1.088	0.857	0.938	1.152	1.232	0.631	0.928	1.192	1.065	1.122	1.057	0.849	0.907	0.915	1.019	0.941	1.030	1.024
c-Myc-R-C	0.927	0.997	0.980	0.999	0.968	0.998	0.950	1.013	0.917	0.936	1.026	0.993	0.940	0.922	0.946	0.936	0.978	0.998	1.018	1.034	1.015	1.025
IGFBP2-R-V	0.911	0.897	0.941	0.940	0.870	0.906	1.029	0.906	0.964	1.184	0.936	0.918	0.897	0.924	0.934	0.932	1.024	0.934	0.939	0.997	1.051	1.052
Atg5-R-C	0.969	1.086	0.944	0.900	1.226	0.956	0.885	1.084	1.116	0.652	1.018	1.079	0.975	1.069	0.947	0.889	1.064	0.918	1.074	1.003	0.957	1.032
Bak-R-C	0.988	1.017	1.075	0.903	0.918	0.908	0.989	0.999	1.081	0.982	0.931	0.965	0.977	1.119	0.989	0.877	1.023	0.934	0.963	0.998	1.015	0.995
GSK-3B-R-C	1.289	1.591	1.273	0.814	0.664	0.895	0.688	1.441	1.156	0.394	1.228	0.831	1.272	1.284	1.296	0.661	0.942	0.843	0.970	1.078	1.003	0.853
Rab11-R-C	1.057	1.081	1.068	0.933	1.034	0.944	0.925	1.008	1.032	0.845	0.929	0.958	1.065	1.040	1.099	0.997	0.990	1.013	0.970	1.000	0.910	0.925
Gli1-R-C	0.876	0.844	0.816	1.047	1.016	1.072	1.124	0.954	0.947	1.248	1.094	1.157	0.860	0.897	0.806	0.989	1.108	1.046	0.997	0.989	1.113	1.180
EphA2-R-V	0.777	0.740	0.738	1.305	1.123	1.385	1.323	0.755	0.857	1.906	1.271	1.560	0.738	0.871	0.750	1.099	1.320	1.061	1.096	0.956	1.362	1.244
Pdcd4-R-C	0.962	0.946	0.709	0.946	1.035	0.885	1.251	1.336	1.143	0.843	2.046	1.334	0.867	0.740	0.895	1.009	0.861	0.927	1.025	1.046	1.510	1.491
cdc2_pY15-R-C	0.938	0.975	0.872	0.963	0.929	0.910	1.049	0.955	1.008	1.047	0.913	0.928	0.870	1.017	0.935	0.904	1.008	0.902	0.996	0.987	1.026	1.067
CD38-R-C	0.927	0.926	0.844	0.987	1.015	0.930	0.985	0.916	0.991	1.070	1.014	0.934	0.945	0.956	0.928	1.002	1.056	0.968	0.969	0.953	0.998	1.022
DUSP6-R-C	1.027	0.953	1.017	1.055	1.056	0.954	1.119	1.230	1.300	1.063	1.140	1.046	0.995	1.161	0.917	0.763	0.989	0.892	1.159	0.975	1.229	1.187
Collagen-VI-R-V	0.990	0.977	0.881	1.005	0.849	0.879	1.363	1.417	1.174	1.044	1.017	1.079	0.891	1.083	0.925	0.773	0.944	0.967	1.018	0.983	1.169	1.364
MelanA-R-C	0.988	0.980	0.970	0.863	0.922	0.896	0.937	1.031	1.138	0.941	0.858	0.958	0.936	1.153	0.980	0.871	1.003	0.926	0.957	0.957	0.985	1.012
GATA6-R-V	0.859	0.827	0.763	1.145	1.023	0.987	1.158	0.886	0.817	1.361	1.162	1.159	0.846	0.804	0.814	0.977	1.078	1.080	1.006	0.994	1.175	1.162
IGF1R_pY1135_Y1136-R-V	0.989	1.028	1.007	0.995	0.957	0.950	1.066	1.135	0.992	0.965	1.007	1.021	0.962	1.073	0.944	0.904	0.975	0.983	1.014	1.016	1.046	1.072
14-3-3-beta-R-V	0.994	1.026	1.002	0.912	1.008	0.973	1.049	1.088	0.983	0.889	1.045	1.039	1.006	1.034	0.991	0.975	0.956	1.004	0.986	0.997	1.013	1.029
A-Raf_pS299-R-C	0.932	0.888	0.911	0.961	0.849	0.913	1.132	0.925	0.969	1.250	0.898	0.943	0.913	0.993	0.936	0.960	1.041	0.914	0.934	0.961	1.061	1.065
G6PD-R-V	0.826	0.861	0.868	1.003	0.891	0.978	1.180	0.792	1.004	1.393	0.905	0.968	0.914	0.941	0.894	1.010	1.227	0.950	0.901	0.929	1.025	1.082
PKM2-R-C	0.802	0.871	0.772	1.065	0.929	1.194	0.927	0.833	1.070	1.004	1.074	1.296	0.826	0.936	0.931	1.129	1.181	1.128	0.927	0.908	0.981	1.051
Glutaminase-R-C	1.346	1.227	1.160	0.972	0.886	0.933	1.119	1.369	1.052	1.043	0.929	0.979	1.204	1.161	1.165	0.870	1.005	0.975	0.979	1.058	1.036	1.003
DNA-Ligase-IV-R-C	1.095	0.968	0.982	0.934	0.869	0.971	1.113	0.890	0.912	1.280	0.776	0.859	1.028	0.720	0.892	0.902	1.031	0.815	1.009	1.153	1.061	1.104
PREX1-R-V	0.972	0.985	1.083	0.869	0.891	0.927	0.954	0.948	1.127	1.053	0.783	0.805	0.921	1.080	0.944	0.862	0.985	0.805	1.013	1.032	0.996	1.029
XBP-1-G-C	1.124	0.897	0.865	0.943	0.885	0.800	1.495	1.237	1.016	1.222	1.005	1.039	1.201	1.062	1.055	1.056	0.961	0.988	0.874	0.870	1.087	1.130
PKCa-R-V	1.937	1.580	1.350	0.977	0.547	0.978	0.866	1.464	1.099	0.649	0.812	0.871	1.756	1.234	1.686	0.765	0.894	0.978	0.949	1.041	0.884	0.733
PDHK1-R-C	0.938	0.973	0.890	0.922	0.988	0.928	1.005	1.001	0.956	0.970	1.035	1.065	0.909	0.937	0.891	0.890	0.947	0.968	1.012	1.023	1.094	1.083
p53-R-C	0.900	0.927	0.912	1.023	0.913	1.019	1.106	0.878	0.926	1.301	0.944	0.957	0.828	0.962	0.922	0.980	1.107	0.937	0.977	1.010	1.059	1.073
XRCC1-R-C	0.986	1.055	0.793	1.077	1.390	1.018	0.947	0.979	1.062	0.891	1.118	1.045	1.030	0.985	0.907	0.975	1.200	1.109	1.061	0.970	0.930	1.022
Erk5-R-V	1.106	1.115	0.959	0.956	0.971	0.850	1.168	1.223	1.121	1.044	0.996	0.901	1.062	1.141	1.068	0.984	1.057	0.918	0.939	0.972	0.994	1.074
Chk2_pT68-R-C	1.019	0.994	1.054	0.940	0.906	0.931	1.026	1.080	1.081	1.036	0.927	0.970	0.958	1.121	0.994	0.881	1.065	0.917	0.970	0.998	1.024	1.037
CD134-R-V	1.084	1.038	1.131	0.912	0.901	0.910	1.106	1.125	1.078	1.024	0.961	0.955	1.074	1.141	1.048	0.969	0.970	0.933	0.948	0.997	1.024	1.014
FOXM1-R-V	0.912	0.909	0.941	0.987	0.869	0.939	1.129	0.932	0.979	1.316	0.898	0.945	0.873	0.969	0.902	0.928	1.084	0.921	0.953	1.006	1.077	1.108
Mnk1-R-V	0.888	0.897	0.914	1.013	1.084	0.956	1.076	0.943	0.962	1.093	0.886	1.000	0.900	0.974	0.855	1.010	1.099	1.043	0.969	0.989	0.945	1.092
Cyclin-D1-R-C	0.969	0.970	0.893	0.963	0.960	0.916	0.998	1.002	0.893	1.039	0.940	0.982	0.928	0.891	0.900	0.925	0.949	0.917	1.017	1.042	1.061	1.064
PDK1_pS241-R-V	1.021	1.082	1.052	1.028	1.164	1.144	0.916	1.115	0.962	0.631	1.100	0.940	1.045	0.987	0.961	1.053	1.136	1.073	1.023	1.054	0.819	1.000
PD-1-R-V	0.895	0.833	0.798	1.144	0.997	1.012	1.294	0.933	0.904	1.471	1.117	1.151	0.833	0.927	0.847	1.048	1.155	1.070	0.963	0.969	1.142	1.201
B7-H3-R-C	0.893	0.885	0.785	1.309	0.976	1.057	1.347	0.909	1.044	1.937	1.061	1.086	0.856	1.004	0.854	1.220	1.452	1.028	0.903	0.944	1.104	1.216
Histone-H3_pS10-R-V	0.754	0.758	0.855	1.257	1.066	1.152	1.346	0.861	0.817	1.831	1.196	1.207	0.828	0.816	0.835	1.184	1.210	1.069	1.004	0.955	1.223	1.220
FN14-R-C	0.891	0.930	0.846	1.230	1.062	1.086	1.278	0.939	0.871	1.820	1.048	1.024	0.925	0.920	0.910	1.140	1.368	0.972	0.971	0.968	1.119	1.121
DAPK2-R-C	1.031	1.015	0.980	0.931	0.863	0.885	1.118	1.089	1.050	1.087	0.944	0.920	0.991	1.023	0.995	0.931	1.031	0.880	0.943	1.005	1.038	1.082

IGFRb-R-C	0.897	1.037	0.764	1.419	1.556	1.177	0.742	0.743	0.758	0.470	1.216	0.842	1.196	0.739	0.936	1.632	1.214	1.432	0.971	0.939	0.591	0.781
PDHA1-R-V	0.937	0.886	0.859	0.936	0.876	0.862	1.135	0.919	1.028	1.134	0.994	0.959	0.979	1.010	0.921	0.947	1.000	0.910	0.936	0.922	1.081	1.059
MITF-R-V	0.948	1.143	0.848	0.971	1.009	0.908	1.116	1.069	0.963	1.143	1.094	1.085	0.887	0.902	0.831	0.884	0.962	0.933	1.039	1.122	1.196	1.201
SETD2-R-C	0.936	0.854	0.826	1.085	0.999	1.019	1.164	0.962	0.843	1.392	1.087	1.066	0.899	0.835	0.856	1.056	1.120	1.001	0.977	1.010	1.116	1.146
AR-R-V	0.920	0.862	0.869	1.049	0.866	0.857	1.185	0.889	1.031	1.251	0.965	0.978	0.875	1.007	0.919	0.923	1.079	0.925	0.947	0.946	1.091	1.109
Gys-R-V	0.943	0.849	1.293	0.923	0.633	0.966	1.269	1.017	1.050	1.476	0.819	0.766	0.970	0.913	0.961	1.000	0.991	1.022	0.837	1.084	1.016	1.173
Bim-R-V	1.352	1.798	0.854	0.958	1.289	0.980	0.699	0.883	0.977	0.540	0.883	1.079	1.111	0.847	1.135	0.945	0.987	1.039	1.086	1.294	0.842	0.827
SOD2-R-V	0.866	0.903	0.803	1.090	1.065	1.017	1.037	0.974	0.931	1.049	1.124	1.124	0.841	0.913	0.839	1.081	1.014	1.094	0.995	0.992	1.034	1.135
IR-b-R-C	0.877	0.881	0.705	1.205	1.409	1.149	1.014	0.961	0.984	0.789	1.181	1.015	1.178	0.937	0.896	1.486	1.195	1.360	0.931	0.818	0.739	0.982
A-Raf-R-V	0.896	1.042	0.892	0.990	0.809	1.090	0.912	0.959	1.109	0.743	1.034	1.110	0.931	0.977	0.959	0.953	1.147	1.034	0.922	0.987	0.921	1.040
INPP4b-R-V	1.108	1.102	1.052	0.947	0.865	0.918	1.093	1.067	1.129	1.085	0.928	0.877	1.083	1.074	1.023	0.901	1.013	0.907	0.968	1.026	1.024	1.034
b-Actin-R-C	1.240	1.098	1.194	0.893	1.010	0.905	1.195	1.244	1.266	1.032	0.881	0.962	1.148	1.133	1.170	0.837	0.953	0.885	1.049	1.023	1.075	1.073
TFAM-R-V	0.848	0.759	0.737	1.294	1.151	1.207	1.299	0.820	0.768	1.746	1.251	1.354	0.819	0.784	0.808	1.197	1.335	1.049	1.020	0.972	1.215	1.197
PARP-R-V	1.010	1.005	0.832	0.965	1.443	1.113	0.884	0.903	1.008	0.869	1.217	1.055	1.242	1.004	0.966	1.150	1.238	1.002	1.039	0.886	0.927	0.870
MSI2-R-C	0.739	0.883	0.701	1.155	1.905	1.231	0.785	0.922	1.051	0.648	1.333	1.262	0.774	0.953	0.834	1.159	1.455	1.488	1.046	0.907	0.790	1.077
CIITA-R-C	0.938	0.856	0.836	1.049	0.928	0.962	1.217	0.934	0.924	1.463	0.965	0.969	0.918	0.904	0.910	1.078	1.152	0.939	0.928	0.963	1.072	1.126
AceCS1-R-V	0.657	0.778	0.749	1.038	0.689	1.061	1.462	0.674	0.797	2.044	0.833	1.206	0.952	0.632	0.787	1.564	1.117	1.216	0.715	0.921	1.048	1.237
c-Kit-R-V	0.906	0.873	0.905	0.921	0.876	0.848	1.042	0.910	0.985	1.138	0.861	0.884	0.852	0.970	0.886	0.886	0.956	0.862	0.978	0.992	1.066	1.085
S100A4-R-V	0.936	0.828	0.693	1.090	0.946	0.924	1.333	0.974	1.019	1.546	1.068	1.073	0.854	1.011	0.888	0.996	1.202	1.042	0.913	0.893	1.138	1.209
PEA-15_pS116-R-V	1.069	1.035	1.142	1.000	0.964	0.935	1.100	1.206	1.141	1.037	0.952	0.958	1.073	1.131	1.013	0.932	0.950	0.923	1.033	1.009	1.050	1.071
Enolase-1-R-V	0.831	0.810	0.767	1.036	1.080	1.044	1.173	0.928	0.913	1.406	1.112	1.210	0.890	0.876	0.800	1.029	1.187	1.071	0.962	0.938	1.134	1.175
PAR-R-C	0.687	0.659	0.462	4.208	4.957	2.551	0.641	0.523	0.656	0.883	2.754	1.624	0.971	0.858	0.392	3.126	4.139	4.480	0.997	0.814	0.448	0.820
Aurora-A-R-C	0.745	0.713	0.662	1.255	1.028	1.187	1.375	0.702	0.779	1.921	1.203	1.302	0.741	0.756	0.674	1.113	1.264	1.098	0.999	0.976	1.274	1.315
FRS2-a_pY196-R-C	0.905	0.940	0.913	1.065	0.876	0.962	1.103	1.021	0.987	1.309	0.928	0.908	0.841	1.007	0.927	0.915	1.136	0.901	0.984	0.994	1.066	1.121
HSP27_pS82-R-V	0.870	0.883	1.127	1.431	0.997	1.218	1.211	0.741	1.010	1.817	0.968	1.077	0.761	0.911	0.881	1.066	1.308	0.979	1.088	1.128	1.152	1.160
RIP3-R-C	0.881	0.847	0.803	1.101	1.013	1.088	1.191	0.952	0.886	1.458	1.096	1.175	0.877	0.912	0.830	1.018	1.152	1.027	1.002	0.966	1.166	1.157
HER3-R-V	0.959	0.994	1.012	1.028	0.898	1.042	1.113	1.062	1.130	1.177	1.067	1.034	0.901	1.032	0.864	0.961	1.033	0.990	0.994	1.060	1.098	1.182
VHL-R-C	0.889	0.842	0.872	1.285	1.173	1.287	1.190	0.696	0.842	1.683	1.084	1.096	0.802	0.819	0.841	1.162	1.281	1.033	1.077	1.057	1.111	1.108
Notch1-cleaved-R-V	0.937	0.907	0.896	0.936	0.870	0.850	1.105	1.004	1.021	1.172	0.907	0.956	0.864	1.016	0.926	0.865	1.030	0.913	0.946	0.977	1.081	1.116
HER2_pY1248-R-C	0.905	0.932	0.914	0.965	0.930	0.916	1.149	0.963	1.077	1.205	0.972	0.977	0.887	1.034	0.869	0.919	1.051	0.940	0.966	0.986	1.084	1.143
Melanoma-gp100-R-C	0.911	0.870	0.872	0.963	0.870	0.874	1.128	1.007	0.969	1.184	0.947	0.930	0.902	1.028	0.921	0.969	1.026	0.905	0.933	0.931	1.055	1.088
IL-6-R-C	0.943	0.892	0.903	0.961	0.867	0.900	1.133	0.993	1.000	1.280	0.971	0.952	0.847	1.012	0.918	0.915	1.071	0.907	0.943	0.986	1.107	1.125
HSP60-R-V	0.762	0.775	0.664	1.529	1.702	1.363	1.149	0.781	0.836	1.417	1.314	1.146	0.782	0.823	0.780	1.594	1.424	1.469	1.024	0.923	0.864	1.160
NRF2-R-C	0.913	0.905	0.841	1.029	0.990	0.998	1.130	1.040	0.953	1.274	1.074	1.123	0.832	0.941	0.877	0.975	1.133	1.018	0.965	1.004	1.110	1.179
YAP-R-C	0.853	0.852	0.793	1.138	1.018	1.112	1.277	0.867	0.935	1.549	1.173	1.203	0.821	0.856	0.808	1.086	1.206	1.067	0.973	1.005	1.169	1.239
CDT1-R-V	0.860	0.828	0.809	1.219	1.108	1.177	1.366	0.948	0.976	1.774	1.211	1.313	0.796	0.924	0.834	1.119	1.301	1.072	1.003	0.977	1.231	1.288
Paxillin-R-C	1.117	1.174	0.914	0.972	1.035	0.929	1.129	1.014	1.135	1.033	0.921	1.032	1.115	1.044	1.066	0.852	1.016	0.941	1.046	0.994	1.063	1.016
p21-R-C	0.866	0.846	0.680	0.733	1.021	1.088	1.316	0.800	0.769	1.694	1.210	1.292	0.852	0.724	0.813	1.011	1.181	1.077	0.869	1.002	1.284	1.208
Rab25-R-V	0.956	0.882	0.933	1.188	0.886	1.050	1.277	0.875	0.968	1.627	1.001	0.967	0.872	1.010	0.876	1.072	1.291	0.945	0.944	1.005	1.087	1.131
ZAP-70-R-C	1.702	1.456	0.922	0.808	0.704	0.834	1.335	1.455	2.288	1.005	0.807	1.044	1.401	1.206	1.194	0.731	0.932	0.757	0.970	1.073	1.180	1.336
cdc25C-R-V	0.880	0.878	0.844	1.289	0.992	1.116	1.370	0.833	0.956	1.935	1.040	1.050	0.879	0.917	0.861	1.133	1.433	0.993	0.954	0.980	1.131	1.189
GRB7-R-V	0.902	0.896	0.904	0.961	0.885	1.010	1.188	0.898	0.965	1.327	0.925	0.939	0.859	0.956	0.876	0.959	1.138	0.883	0.959	1.004	1.070	1.134
TFRC-R-V	0.852	0.786	0.803	1.305	0.966	0.977	1.373	0.805	0.718	1.852	1.205	1.164	0.779	0.846	0.639	1.009	1.060	1.069	1.035	1.079	1.345	1.279
MERIT40-R-C	0.898	0.979	0.867	1.004	1.109	1.011	1.128	0.880	0.977	1.209	0.989	1.005	0.880	0.914	0.875	0.982	1.081	0.964	1.032	1.028	1.058	1.119
UVRAG-R-C	0.873	0.848	0.797	1.174	0.937	1.009	1.377	0.866	1.005	1.776	1.080	1.056	0.825	0.907	0.867	1.052	1.308	1.040	0.917	0.968	1.150	1.249
c-Abl_pY412-R-C	0.908	0.872	0.895	1.103	0.873	0.989	1.200	0.974	1.018	1.520	1.024	1.037	0.863	0.993	0.927	0.984	1.227	0.965	0.934	0.961	1.127	1.147
Aurora-B-R-V	0.863	0.866	0.779	1.143	1.602	1.325	1.026	0.890	0.860	1.195	1.082	1.317	0.807	0.844	0.814	1.801	1.129	1.277	0.967	1.018	0.854	1.127
CD4-R-V	0.893	0.840	0.831	1.097	0.921	0.960	1.360	0.892	1.047	1.732	1.100	1.082	0.847	0.996	0.883	1.017	1.207	0.994	0.925	0.941	1.217	1.210
SHP2-R-V	1.086	1.173	1.118	0.809	0.927	0.907	1.536	1.220	1.148	0.917	0.852	0.905	1.011	0.935	1.114	0.779	0.838	0.856	1.069	1.104	1.081	1.276
Caspase-8-cleaved-R-C	0.813	0.707	0.755	1.168	0.955	1.031	1.388	0.915	0.887	1.956	1.023	1.129	0.866	0.823	0.859	1.067	1.265	0.969	0.955	0.893	1.245	1.252
Caspase-3-cleaved-R-C	0.883	0.858	0.831	1.130	0.994	1.043	1.314	0.885	1.016	1.637	0.963	1.020	0.892	0.913	0.891	1.067	1.215	0.987	0.969	0.954	1.107	1.193
PR-R-V	0.900	0.876	0.848	1.069	0.966	1.050	1.155	0.958	0.933	1.401	1.082	1.132	0.781	0.905	0.796	0.978	1.138	1.007	0.988	1.057	1.158	1.227
Cdc42-R-C	0.936	0.902	0.840	0.961	0.972	0.968	1.106	0.982	0.932	1.192	1.044	1.073	0.915	0.912	0.885	0.963	1.028	0.982	0.976	0.987	1.113	1.114
IRF-1-R-C	0.890	0.876	0.857	0.919	0.913	0.896	1.265	1.065	0.958	1.147	1.009	1.021	0.930	0.930	0.909	0.948	1.013	0.981	0.927	0.948	1.080	1.188
Coup-TFII-R-C	1.255	1.442	0.713	1.142	1.228	0.911	0.890	1.146	1.148	0.386	1.252	1.030	1.171	0.878	1.320	0.700	0.993	1.143	1.156	1.012	0.940	0.945
Wee1-R-C	0.843	0.895	0.869	1.181	0.982	1.091	1.238	0.801	0.935	1.658	0.964	1.030	0.838	0.877	0.878	1.106	1.356	0.997	0.940	1.006	1.056	1.147
DUSP4-R-V	0.764	0.745	0.655	1.360	1.101	1.200	1.415	0.692	0.731	2.060	1.167	1.223	0.723	0.679	0.673	1.176	1.324	1.096	1.018	1.044	1.237	1.368
BMK1-Erk5_pT218_Y220-R-V	0.896	0.933	0.898	0.980	0.999	0.968	1.030	1.046	0.999	1.040	1.109	1.116	0.852	1.009	0.864	0.937	0.986	1.033	0.997	1.001	1.105	1.129
Shc_pY317-R-V	0.914	0.980	0.946	0.993	0.874	0.906	1.033	1.000	1.021	1.203	0.911	0.887	0.884	1.061	0.888	0.889	1.057	0.869	0.985	1.002	1.066	1.078
4E-BP1-R-V	0.900	1.109	0.986	0.939	1.333	1.061	0.879	0.880	0.920	0.855	0.776	0.819	0.961	0.915	0.964	1.005	1.054	1.030	1.079	1.054	0.793	0.943
TSC1-R-C	1.027	1.166	1.022	0.957	0.889	0.980	0.985	1.189	1.161	0.733	0.980	0.939	0.935	1.045	0.922	0.798	1.065	0.952	1.004	1.108	0.942	1.149
Mitofusin-1-R-V	0.850	0.827	0.726	1.375	0.995	1.177	1.544	0.864	0.899	2.294	1.140	1.162	0.813	0.847	0.793	1.239	1.529	1.050	0.929	0.980	1.204	1.348
PTEN-R-V	0.480	0.580	0.517	1.002	1.677	1.209	0.368	0.476	0.393	0.394	1.263	0.534	1.489	0.624	0.944	1.587	1.000	1.269	1.008	0.516	0.568	0.405
Ambra1_pS52-R-C	0.945	0.949	0.897	0.902	0.963	0.895	1.030	1.072	1.003	1.013	1.078	1.042	0.908	0.988	0.896	0.894	0.960	1.010	0.963	0.999	1.094	1.112
H2AX_pS139-R-V	0.826	0.826	0.772	1.294	1.280	1.283	1.331	0.973	0.972	1.786	1.233	1.322	0.820	0.924	0.780	1.243	1.389	1.192	1.009	0.960	1.135	1.297
MEK1-R-V	0.895	0.901	0.838	1.227	1.070	1.256	1.343	0.815	0.996	1.934	1.018	1.101	0.801	0.949	0.878	1.121	1.363	1.000	1.020	1.002	1.163	1.200
PARG-R-C	0.750	0.706	0.671	1.581	1.819	1.490	1.357	0.780	0.838	1.823	1.322	1.083	0.729	0.795	0.708	1.607	1.869	1.466	0.990	0.953	0.856	1.333
CDKN2A-R-C	0.671	0.665	0.589	2.090	1.009	1.000	1.444	0.770	0.774	3.510	1.140	1.174	0.681	0.754	0.638	0.925	1.123	1.098	1.303	0.928	1.851	1.441
Calnexin-R-V	0.742	0.631	0.503	1.128	0.921	0.936	1.391	0.738	0.696	1.690	1.193	1.126	0.724	0.680	0.639	1.033	1.211	1.054	0.905	0.918	1.216	1.383
PLK1-R-C	0.768	0.766	0.806	1.457	1.154	1.253	1.430	0.789	0.822	1.950	1.060	1.156	0.723	0.705	0.667	1.199	1.317	1.101	1.068	1.117	1.152	1.452
IDO-R-C	0.884	0.852	0.760	1.266	1.057	1.196	1.495	1.004	0.970	2.149	1.072	1.162	0.821	0.903	0.847	1.127	1.379	0.980	1.009	0.971	1.257	1.350
XIAP-R-C	1.092	1.269	1.180	0.962	1.019	0.950	0.870	1.150	1.081	0.724	1.014	0.957	1.087	1.000	1.106	0.988	0.926	0.964	1.019	1.109	0.937	0.971
LDHA-R-C	0.896	0.841	0.781	1.187	0.958	1.049	1.423	1.024	1.044	1.806	1.076	1.128	0.831	0.986	0.849	1.108	1.341	1.013	0.923	0.945	1.158	1.310
CA9-R-C	0.884	0.871	0.795	1.284	0.970	1.114	1.463	1.024	1.020	1.949	1.103	1.063	0.843	0.964	0.848	1.173	1.442	1.017	0.927	0.960	1.133	1.321
CtIP-R-V	0.869	0.801	0.785	1.373	1.018	1.313	1.629	0.847	1.046	2.283	1.197	1.215	0.811	0.926	0.833	1.264	1.522	1.050	0.966	0.955	1.224	1.371
ACC1-R-C	0.808	0.792	0.965	1.222	0.594	1.204	3.596	0.773	1.071	5.493	0.834	0.880	0.734	0.886	0.928	0.860	1.056	0.995	1.038	1.007	2.476	2.135
TRIM25-R-C	0.891	0.945	1.010	1.265	0.932	1.168	1.359	0.880	0.939	1.778	1.041	1.082	0.830	0.944	0.844	1.055	1.238	0.955	1.036	1.088	1.201	1.214
RIP-R-C	0.823	0.778	0.749	1.296	1.117	1.271	1.352	0.818	0.807	1.944	1.223	1.377	0.773	0.747	0.746	1.149	1.310	1.189	1.010	1.037	1.245	1.314
MLKL-R-V	0.867	0.861	0.891	1.142	0.953	1.112	1.388	0.787	0.898	1.932	1.074	1.029	0.814	0.858	0.806	1.096	1.252	0.967	0.968	1.057	1.217	1.240
Rb_pS807_S811-R-V	0.817	0.856	0.964	1.297	1.127	1.141	0.897	0.846	0.771	1.057	1.561	0.984	0.759	0.860	0.827	1.277	0.977	1.252	1.017	1.078	1.027	1.028
IRS2-R-C	0.966	1.128	0.875	1.060	0.889	0.922	0.800	0.829	0.793	0.961	0.871	0.714	1.197	0.712	0.953	1.360	0.924	0.939	0.891	1.037	0.790	0.846
Cyclin-B1-R-V	0.812	0.875	0.982	1.215	1.005	1.139	1.416	0.837	0.931	1.946	1.006	1.048	0.750	0.886	0.793	1.106	1.201	0.920	1.041	1.098	1.240	1.310
FASN-R-V	0.868	0.795	0.814	1.350	0.566	1.270	3.021	0.736	1.234	5.034	0.865	1.007	0.764	0.945	0.905	0.975	1.188	0.860	1.054	0.947	2.284	1.909
Cox-IV-R-V	0.871	0.827	1.147	1.260	0.974	1.198	1.528	0.865	0.870	1.664	1.132	1.077	0.790	0.864	0.862	1.143	1.055	0.969	1.084	1.131	1.223	1.297
Granzyme-B-R-V	1.206	1.512	0.866	0.768	0.657	0.730	0.959	2.491	4.293	0.831	0.661	0.733	1.228	1.186	1.386	0.754	0.851	0.732	0.922	0.943	0.952	2.038
Caspase-7-cleaved--R-C	1.103	1.411	1.134	0.800	0.873	0.847	1.073	1.998	2.992	0.807	0.706	0.740	1.356	1.127	1.339	0.994	0.800	0.802	0.971	0.954	0.868	1.586
Rictor-R-C	1.473	1.393	1.603	0.796	0.543	0.688	0.806	1.643	1.327	0.482	0.619	0.561	1.297	1.370	1.191	0.642	0.878	0.863	0.850	1.158	0.697	0.979
ACSL1-R-V	0.780	0.534	0.565	1.755	0.676	1.661	4.085	0.901	2.051	6.256	0.985	0.755	0.549	0.676	0.651	0.867	1.203	1.226	1.242	1.001	2.426	3.750
MYH11-R-C	0.928	0.974	0.524	0.851	0.691	0.916	1.111	1.495	1.482	0.758	0.219	0.218	0.829	1.471	0.442	0.906	0.787	0.827	0.975	0.884	0.474	1.491

Supplementary Table 2. Differentially expressed between G12D mice (Amhr2-Cre Pten fl/fl KrasG12D/+) versus Control mice (Amhr2-Cre Ptenfl/fl)

Name	Chromosome	Region	Max group mean	Log_2 fold change	Fold change	P-value	FDR p-value
Tff1	17	complement(31161396..31165053)	96.77	12.65	6,446.65	3.75E-13	4.21E-09
Ces2f	8	104947356..104960047	1.26	10.27	1,234.83	3.12E-10	5.38E-07
Muc5b	7	141839070..141873084	30.8	9.49	720.55	1.03E-11	4.61E-08
Htr3a	9	complement(48899213..48911099)	0.61	9.2	587.03	4.07E-05	7.68E-03
Teddm3	16	complement(21152655..21154000)	0.78	8.97	500.49	3.79E-05	7.32E-03
Dsg1a	18	20310811..20343350	0.18	8.97	499.78	2.69E-05	5.59E-03
Spink5	18	43963235..44022501	8.28	8.78	438.81	2.53E-09	2.99E-06
Ern2	7	complement(122169893..122186207)	1.88	8.75	429.24	1.47E-08	1.22E-05
Hoxd13	2	74668310..74671599	0.37	8.73	424.14	7.45E-05	1.00E-02
Olfm4	14	79984081..80023139	109.87	8.68	410.81	9.76E-10	1.37E-06
Cyp4a12b	4	115411624..115439034	1.54	8.62	392.21	5.98E-08	3.73E-05
Krt6a	15	complement(101689932..101694307)	54.71	8.47	354.38	2.25E-10	5.04E-07
Pitx1	13	complement(55825051..55836192)	5.01	8.35	326.78	4.75E-10	7.60E-07
Krt33b	11	complement(100023634..100029868)	0.44	8.31	318.21	1.84E-04	3.00E-02
Krt5	15	complement(101707070..101712891)	258.34	8.25	305.28	2.31E-11	8.63E-08
Gm94	18	complement(43777196..43792878)	2.77	8.19	292.9	4.56E-07	1.94E-04
Krt14	11	complement(100203162..100207548)	203.51	8.13	279.65	5.05E-12	2.83E-08
Ces1h	8	complement(93351843..93379725)	0.29	7.98	253.29	2.28E-04	0.03
Gpr87	3	complement(59178923..59195104)	1.28	7.97	251.42	2.65E-08	1.86E-05
Wnt3a	11	complement(59248033..59290752)	0.17	7.79	220.56	2.70E-04	3.00E-02
Acsm1	7	119607026..119662515	0.2	7.67	203.29	3.97E-04	0.05
Kcnj16	11	110968033..111027968	2.09	7.61	195.8	2.25E-09	2.81E-06
Lypd3	7	24636550..24641118	3.95	7.58	190.99	1.77E-09	2.34E-06
Gm44985	7	complement(122167029..122168044)	1.91	7.43	172.18	5.01E-07	2.08E-04
Gp2	7	complement(119442537..119459285)	9.80E-01	7.36	164.81	2.39E-06	7.88E-04
Cbln1	8	complement(87468405..87472609)	1.56	7.35	163.49	3.66E-08	2.49E-05
Pglyrp4	3	90726906..90741517	0.51	7.31	158.8	2.67E-06	8.29E-04
Psca	15	74714839..74717069	9.49	7.26	152.83	8.85E-08	5.36E-05
Lypd8	11	58379043..58390728	12.42	7.23	150.29	2.45E-07	1.25E-04
Gm9573	17	complement(35617923..35626637)	0.74	7.19	146.09	6.21E-07	2.44E-04
Krt6b	15	complement(101676034..101680289)	32.72	7.04	131.79	4.59E-07	1.94E-04
Pkp1	1	complement(135871395..135919207)	12.29	6.96	124.32	6.93E-11	1.73E-07
Fam25c	14	complement(34351881..34355433)	1.44	6.94	123.17	5.30E-05	9.68E-03
Foxn1	11	complement(78357577..78386558)	0.45	6.92	121.15	2.08E-07	1.14E-04
Muc13	16	33794037..33819934	10.35	6.91	120.32	3.10E-10	5.38E-07
Krt16	11	complement(100246091..100248902)	5.49	6.85	115.01	3.13E-07	1.53E-04
Krt15	11	complement(100131758..100135928)	45.1	6.83	113.73	7.44E-10	1.11E-06
Cxcl3	5	90786103..90789600	2.8	6.78	109.81	3.56E-06	1.01E-03
Gm5478	15	complement(101643024..101647380)	1.51	6.73	106.07	4.26E-07	1.94E-04
Nccrp1	7	complement(28543596..28547254)	12.43	6.67	101.51	6.34E-11	1.73E-07
Snap25	2	136713453..136782428	0.3	6.53	92.16	2.64E-05	5.59E-03
Krt13	11	complement(100117327..100121566)	82.51	6.49	90.17	8.55E-09	7.99E-06
Rptn	3	93393699..93399442	0.32	6.45	87.3	3.41E-06	9.81E-04
Dsg1b	18	20376729..20410196	0.44	6.42	85.66	8.02E-07	2.90E-04
Foxa1	12	complement(57540628..57546916)	0.17	6.27	77.31	1.75E-04	3.00E-02
Aldh3a1	11	61207537..61218421	8.49	6.24	75.7	4.66E-09	4.75E-06
Krtdap	7	30787896..30791097	2.27	5.98	63.18	2.23E-06	7.46E-04
Ankk1	9	complement(49415194..49427021)	0.26	5.96	62.11	9.86E-06	2.46E-03
Gzmg	14	complement(56156582..56159579)	8.32	5.87	58.31	2.56E-06	8.29E-04
Tfap2a	13	complement(40715302..40738376)	1.21	5.83	56.92	1.01E-08	9.07E-06
Atp6v1b1	6	83742990..83758855	0.18	5.79	55.5	3.17E-05	6.34E-03
Fat2	11	complement(55250609..55336564)	1.57	5.75	53.83	2.10E-08	1.52E-05
Cnfn	7	complement(25367620..25369724)	0.65	5.72	52.85	1.84E-04	0.03
Foxa3	7	complement(19013284..19023538)	0.51	5.72	52.65	9.49E-06	2.42E-03
Ly6g6c	17	35065388..35070050	1.44	5.69	51.45	2.74E-06	8.29E-04
Upk3a	15	85017141..85022560	0.34	5.67	50.94	1.40E-04	0.02
Chrna3	9	complement(55010111..55026562)	0.07	5.62	49.11	2.32E-04	0.03
Trim29	9	43310848..43336115	7.87	5.57	47.61	4.54E-09	4.75E-06
Ly6d	15	complement(74762056..74763620)	62.51	5.51	45.42	1.98E-08	1.48E-05
Dsc3	18	complement(19960930..20002351)	1.93	5.47	44.18	7.03E-07	2.63E-04
Nefl	14	68083863..68089095	0.32	5.44	43.45	1.28E-04	2.00E-02
Grhl3	4	complement(135541888..135573630)	2.71	5.39	41.87	7.19E-09	7.01E-06
Slc5a7	17	complement(54273594..54299034)	0.12	5.38	41.54	8.88E-07	3.16E-04
Col17a1	19	complement(47646344..47692094)	16.33	5.36	41.17	3.34E-12	2.50E-08
Spdef	17	complement(27714352..27728951)	7.13	5.35	40.79	1.85E-08	1.43E-05
Btnl4	17	complement(34469042..34475937)	0.17	5.31	39.75	1.35E-04	0.02
Tph2	10	complement(115078641..115185022)	0.64	5.29	39.2	2.17E-06	7.37E-04
Lypd2	15	complement(74732247..74734329)	14.45	5.27	38.5	9.47E-08	5.59E-05

Tprg	16	25286817..25422344	1.64	5.26	38.38	6.41E-07	2.44E-04
Pirt	11	66911981..66929876	0.13	5.14	35.29	1.13E-04	0.02
Cyp4a12a	4	115299046..115332815	4.25	5.14	35.21	1.24E-06	4.28E-04
Gzme	14	complement(56117626..56120625)	3.73	5.12	34.73	2.73E-06	8.29E-04
Sptssb	3	complement(69819542..69859940)	1.53	4.95	30.99	6.39E-07	2.44E-04
Prom2	2	complement(127526473..127541467)	1.17	4.95	30.85	2.63E-07	1.31E-04
Chad	11	94565047..94569127	12.89	4.93	30.55	4.58E-07	1.94E-04
Gzmd	14	complement(56129556..56132608)	10.77	4.91	30.13	1.07E-06	3.77E-04
Isl1	13	complement(116298281..116309689)	0.21	4.89	29.7	2.36E-04	0.03
Lipm	19	34100943..34122687	0.48	4.83	28.47	1.01E-04	0.02
Krt78	15	complement(101946004..101954287)	0.61	4.82	28.32	5.37E-05	9.72E-03
Snx31	15	complement(36504062..36555573)	0.31	4.77	27.31	4.20E-05	7.85E-03
Aqp4	18	complement(15389394..15403684)	2.57	4.74	26.7	1.85E-05	4.18E-03
S100a14	3	90526856..90528837	16.05	4.72	26.42	3.54E-07	1.65E-04
Calml3	13	complement(3802896..3804316)	15.03	4.72	26.39	4.56E-07	1.94E-04
Dlx3	11	95120119..95125296	0.12	4.71	26.09	3.79E-04	5.00E-02
Sdr9c7	10	127898518..127911761	0.32	4.67	25.4	2.47E-04	0.03
Dsg3	18	20510304..20541310	6.05	4.66	25.33	3.62E-05	7.12E-03
Tgm5	2	complement(121046111..121085841)	1.99	4.53	23.14	3.91E-05	7.49E-03
Lgals7	7	28863853..28866284	11.86	4.49	22.43	5.11E-07	2.08E-04
Serpina3j	12	104314553..104320725	0.12	4.48	22.38	2.38E-04	3.00E-02
St6galnac1	11	complement(116765025..116775507)	3.61	4.46	22.08	2.20E-07	1.17E-04
Dapl1	2	59484653..59505020	11.01	4.44	21.77	1.29E-05	3.15E-03
Serpinb2	1	107511423..107535478	0.79	4.3	19.71	3.31E-05	6.58E-03
Rhov	2	complement(119269201..119271272)	4.2	4.29	19.57	2.43E-05	5.34E-03
Btnl6	17	complement(34507804..34517352)	0.39	4.26	19.15	1.93E-04	0.03
Slc9a4	1	40580081..40630725	0.17	4.24	18.91	2.73E-06	8.29E-04
Tmprss11b	5	complement(86657631..86676362)	7.46	4.18	18.1	3.02E-04	4.00E-02
C8a	4	complement(104815679..104876398)	0.25	4.17	17.98	6.96E-06	1.79E-03
Klhdc8a	1	132298626..132307357	1.19	4.15	17.8	4.09E-06	1.15E-03
Anxa8	14	34085981..34100571	28.01	4.09	16.98	1.27E-07	7.14E-05
BC016579	16	complement(45626848..45654118)	0.4	4.06	16.67	2.36E-04	0.03
Slc10a4	5	73006883..73012955	0.19	4.02	16.21	1.45E-04	2.00E-02
Trp63	16	25683763..25892102	2.48	4	15.96	7.17E-07	2.64E-04
Prr32	X	45090904..45092791	0.32	3.94	15.32	2.60E-04	3.00E-02
Aqp3	4	complement(41092722..41098183)	4.5	3.93	15.24	4.48E-06	1.24E-03
Dmkn	7	30763756..30781063	3.01E+00	3.86	14.5	4.02E-04	0.05
Timp4	6	complement(115241892..115252205)	1.21	3.75	13.41	1.65E-05	3.86E-03
Ugt1a1	1	88211959..88218997	4.02	3.72	13.21	1.05E-04	2.00E-02
Ces1f	8	complement(93256236..93279747)	2.47	3.68	12.79	2.63E-05	5.59E-03
Ptprn2	12	116485720..117276849	0.37	3.65	12.55	1.23E-04	0.02
Capn13	17	complement(73306464..73399296)	0.66	3.65	12.52	2.14E-04	0.03
Ccdc129	6	55836895..55978735	1.21	3.62	12.33	1.36E-05	3.28E-03
Kcnj15	16	95257558..95300260	1.57	3.6	12.15	2.96E-05	6.03E-03
Serpinb11	1	107361198..107380475	30.52	3.6	12.12	5.61E-06	1.52E-03
Caln1	5	130369455..130847412	0.03	3.54	11.65	1.53E-04	0.02
Mmp12	9	7344381..7369499	7.22	3.53	11.58	6.17E-06	1.63E-03
Muc5ac	7	141788972..141819231	0.37	3.52	11.45	1.15E-04	0.02
Kcnj5	9	complement(32314707..32344350)	0.04	3.5	11.29	1.45E-04	0.02
Aldh3b2	19	3972328..3981645	2.32	3.42	10.68	2.95E-06	8.83E-04
Slc24a2	4	complement(86983124..87230477)	0.03	3.37	10.37	3.30E-04	4.00E-02
Duox1	2	122315672..122347972	0.81	3.36	10.27	4.16E-04	0.05
Serpinb10	1	107529003..107549271	2.06	3.34	10.15	1.87E-04	0.03
Cyp2f2	7	27119909..27133660	38.69	3.31	9.93	3.13E-06	9.23E-04
Fabp4	3	complement(10204088..10208576)	216.77	3.3	9.85	2.13E-05	4.77E-03
Dlk2	17	46297421..46303271	0.71	3.25	9.5	7.90E-05	1.00E-02
Pla2g2c	4	138724792..138746132	0.17	3.12	8.69	3.43E-04	0.04
Zfp750	11	complement(121510978..121519333)	5.71	3.07	8.42	1.88E-04	0.03
Lhfpl1	X	complement(145290359..145349089)	0.43	3	8.02	1.31E-04	2.00E-02
Upk1a	7	complement(30603092..30612847)	6.77	2.97	7.85	4.06E-05	7.68E-03
Gja8	3	complement(96913566..96926020)	0.13	2.96	7.79	1.69E-04	3.00E-02
Kcnh1	1	192190774..192510159	0.72	2.88	7.34	4.92E-05	9.12E-03
Tmc5	7	118597297..118675086	0.64	2.85	7.2	1.92E-04	3.00E-02
Foxq1	13	31556134..31560976	3.29	2.69	6.46	2.11E-04	3.00E-02
Esrrg	1	187608791..188214885	0.45	2.69	6.44	3.45E-04	0.04
Galnt6	15	complement(100691813..100729376)	9.9	2.68	6.42	1.82E-04	0.03
Six1	12	complement(73040015..73053887)	6.33	2.61	6.09	4.22E-04	0.05
A2m	6	121635376..121679227	76.89	2.57	5.95	2.13E-04	3.00E-02
Crybg2	4	134060815..134092504	0.54	2.53	5.77	3.93E-04	0.05
Sez6l	5	complement(112419151..112577185)	1.99	2.49	5.61	9.73E-06	2.45E-03
Il33	19	29925114..29960718	14.84	2.41	5.31	6.66E-05	0.01

Tmem182		1	40805601..40856887	2.02	2.41	5.3	3.03E-05	6.13E-03
Ephx3		17	complement(32183770..32189549)	1.3	2.4	5.28	1.84E-04	0.03
Ttc29		8	78213297..78394326	0.31	2.37	5.17	2.55E-04	3.00E-02
Lgals12		19	complement(7596660..7607193)	3.67	2.36	5.14	6.46E-05	1.00E-02
Lrmp		6	145115653..145174934	15.86	2.05	4.15	2.41E-04	0.03
Col28a1		6	complement(7997808..8192617)	1.04	1.8	3.49	2.90E-04	0.04
Bmp5		9	75775364..75900310	1.49	1.7	3.24	2.23E-04	0.03
Fyb2		4	104913456..105016863	14.28	1.66	3.17	4.03E-04	5.00E-02
Meox2		12	37108540..37179534	2.68	1.55	2.94	3.24E-04	0.04
Lgi2		5	complement(52533517..52566462)	9.05	1.5	2.84	1.71E-04	0.03
Plxdc1		11	complement(97923238..97986444)	5.62	1.45	2.73	6.25E-06	1.63E-03
Chrdl1	X		complement(143285674..143394262)	5.49	1.36	2.57	1.43E-05	3.40E-03
Ccnd2		6	complement(127125162..127152193)	17.36	1.04	2.06	3.74E-05	7.30E-03
Slc39a8		3	135825279..135888572	13.78	-1.1	-2.14	1.49E-04	0.02
Slc5a3		16	92058322..92087473	4.53	-1.26	-2.4	1.80E-04	0.03
Napsa		7	44572380..44586862	2.39E+01	-1.85	-3.61	3.77E-04	0.05
Nmb		7	complement(80902227..80905076)	18.5	-2.34	-5.07	9.81E-05	0.02
Fcer2a		8	complement(3681737..3694175)	0.38	-2.5	-5.66	2.96E-04	4.00E-02
Tll1		8	complement(64014931..64206271)	3.64	-2.52	-5.74	1.70E-05	3.93E-03
Frmpd1		4	45184875..45285936	0.53	-2.7	-6.5	2.60E-05	5.59E-03
Nog		11	complement(89300638..89302332)	7.8	-2.74	-6.7	6.09E-07	2.44E-04
Ighv1-53		12	complement(115158403..115158835)	39.69	-2.94	-7.65	2.66E-05	5.59E-03
Fcrl5		3	87435773..87500678	0.12	-2.98	-7.91	3.69E-04	0.04
Serpina1b		12	complement(103728156..103830373)	1.43	-3.14	-8.79	1.94E-04	3.00E-02
Aqp8		7	123462291..123468004	13.7	-3.21	-9.24	1.63E-04	0.02
Hal		10	93488768..93519304	0.85	-3.3	-9.85	5.31E-05	9.68E-03
Trpm1		7	64153835..64269775	0.19	-3.42	-10.67	5.53E-06	1.51E-03
Gm37465		3	complement(52000736..52004025)	0.06	-3.47	-11.1	9.51E-05	0.02
Plppr1		4	49059273..49340259	0.43	-3.55	-11.75	3.01E-04	0.04
Klk1		7	44225360..44229618	5.65	-3.56	-11.77	7.34E-05	0.01
Cyp26a1		19	37697808..37701528	7.85	-3.66	-12.65	1.03E-05	2.55E-03
Tpm3-rs7		14	113314608..113316754	6.09	-3.86	-14.54	4.39E-09	4.75E-06
Igkv3-5		6	70663275..70663895	12.56	-3.9	-14.97	2.72E-05	5.61E-03
Gdpd3		7	126766334..126775649	20.2	-4.09	-17.05	3.47E-07	1.65E-04
Calb2		8	complement(110137502..110168210)	1.38	-4.2	-18.39	6.24E-06	1.63E-03
Gm7298		6	121732932..121789084	2.27	-4.31	-19.87	6.85E-05	0.01
Pcna-ps2		19	9283238..9284494	0.85	-4.47	-22.22	1.84E-05	4.18E-03
Adarb2		13	8202866..8768747	0.58	-4.56	-23.58	5.16E-08	3.39E-05
Igkv3-2		6	70698449..70699067	71.64	-4.66	-25.26	2.25E-07	1.17E-04
Tm4sf4		3	57425314..57441677	0.88	-4.87	-29.34	2.67E-05	5.59E-03
Igkv3-10		6	70572633..70573230	16.97	-4.91	-30.09	3.37E-06	9.81E-04
Gm12666		4	complement(92190744..92191749)	0.89	-5.54	-46.4	1.12E-07	6.44E-05
Gm9008		6	complement(76495432..76497784)	0.44	-5.61	-48.79	2.66E-06	8.29E-04
Serpina1a		12	complement(103853589..103863562)	2.89	-5.65	-50.28	5.28E-08	3.39E-05
Slc17a4		13	complement(23895890..23915009)	0.12	-6.45	-87.58	1.47E-05	3.46E-03
Spaca7		8	12573029..12600744	0.21	-6.61	-97.93	3.18E-04	4.00E-02
Gm3500		14	complement(5365794..5389049)	0.15	-7.05	-132.77	1.01E-04	0.02
Gm20503		4	59003210..59041888	0.21	-7.06	-133.1	1.33E-04	0.02
Igkv3-9		6	70588189..70588777	0.51	-7.16	-143.25	2.35E-05	5.22E-03
Spink1		18	complement(43728069..43737237)	55.68	-7.42	-170.69	1.43E-08	1.22E-05
Serpina1c		12	complement(103894926..103904887)	4.61	-7.56	-188.59	2.70E-10	5.38E-07
Prss28		17	25308646..25311876	105.9	-8.31	-316.96	3.07E-11	9.85E-08
Ighv1-62-2		12	complement(115446409..115446869)	5.54	-8.45	-349.68	1.83E-08	1.43E-05
Prss29		17	25318654..25322681	334.33	-9.47	-708.62	1.16E-13	2.60E-09

Supplementary Table 3. Differentially expressed between G12V mice (Amhr2-Cre Pten fl/fl KrasG12V/+) versus Control mice (Amhr2-Cre Ptenfl/fl)

Name	Chromosome	Region	Max group mean	Log_2 fold change	Fold change	P-value	FDR p-value
Ptprn	1	complement(75247027..75264502)	6.68	8.58	383.05	7.29E-14	1.64E-09
Grem1	2	complement(113746164..113758646)	3.83	8.51	365.09	2.13E-11	1.59E-07
Ngp	9	110419747..110423012	22.93	8.03	261.73	1.62E-07	5.15E-04
Cuzd1	7	complement(131308554..131322292)	0.20	8.02	258.98	2.38E-04	0.05
Ereg	5	91074622..91093646	0.38	7.59	192.52	3.54E-07	6.85E-04
Mrgpra2a	7	complement(47426328..47452139)	0.53	6.87	117.23	1.36E-04	0.04
Syndig1l	12	complement(84677277..84698831)	2.10	6.81	112.27	2.71E-07	6.76E-04
Cxcl3	5	90786103..90789600	2.14	6.77	109.15	3.41E-05	0.02
Saa3	7	complement(46711998..46715700)	264.26	6.74	106.87	5.24E-07	7.83E-04
Myh2	11	67171027..67197517	0.30	6.66	101.36	1.08E-05	8.65E-03
Camp	9	complement(109847379..109849617)	3.41	6.6	97.09	1.91E-05	0.01
Mmp8	9	7558456..7568485	15.91	6.58	95.9	1.84E-07	5.15E-04
Ces2f	8	104947356..104960047	0.07	6.41	85.14	2.39E-04	0.05
Ifi44l	3	complement(151758737..151762892)	1.11	6.23	75.27	8.07E-07	1.13E-03
S100a8	3	90668978..90670035	365.60	6.18	72.26	1.48E-05	9.00E-03
Itgb2l	16	complement(96422288..96443619)	0.66	6.14	70.57	4.62E-06	4.50E-03
S100a9	3	complement(90692632..90695721)	582.94	6.12	69.36	2.18E-05	0.01
Thbs4	13	complement(92751590..92794818)	59.08	5.83	56.77	1.76E-11	1.59E-07
Rph3a	5	complement(120940499..121010092)	0.17	5.59	48.05	9.05E-06	7.52E-03
Igfbp2	1	72824503..72852474	144.38	5.28	38.96	1.04E-08	4.64E-05
Dspp	5	104170712..104180127	0.13	5.25	37.99	9.95E-05	0.03
Tarm1	7	complement(3486500..3502624)	1.88	5.18	36.32	1.22E-05	8.66E-03
Cd177	7	complement(24743983..24760311)	7.01	5.09	34.06	3.97E-07	6.85E-04
Wfdc21	11	83746940..83752642	18.32	5.07	33.48	1.77E-05	0.01
Ndst4	3	125404076..125728899	0.07	5.01	32.12	1.26E-05	8.66E-03
Prok2	6	complement(99711299..99726392)	8.20	5	31.9	3.56E-08	1.33E-04
Kng1	16	23057865..23082068	1.37	4.98	31.46	1.75E-06	1.96E-03
Bco1	8	117095854..117133720	3.76	4.95	30.92	3.68E-07	6.85E-04
Slc24a2	4	complement(86983124..87230477)	0.08	4.94	30.72	1.67E-06	1.96E-03
Pnoc	14	complement(65400673..65425472)	1.31	4.87	29.22	5.12E-05	0.02
Glycam1	15	complement(103562759..103565081)	2.56	4.81	28.1	1.15E-04	0.03
Mmp13	9	7272514..7283331	1.03	4.63	24.82	1.92E-05	0.01
Tph2	10	complement(115078641..115185022)	0.37	4.61	24.37	2.26E-04	0.05
Ankrd1	19	complement(36111965..36119844)	1.90	4.5	22.57	2.17E-05	0.01
Adgra1	7	139834174..139878088	2.17	4.47	22.09	1.36E-06	1.69E-03
Col10a1	10	34389981..34397085	0.27	4.45	21.86	2.31E-04	0.05
Timp1	X	20870166..20874735	116.72	4.42	21.36	3.54E-10	1.99E-06
Cxcl13	5	95956951..95961068	25.09	4.28	19.48	2.59E-04	5.00E-02
Il6	5	30013114..30019981	0.58	4.25	18.99	6.68E-05	2.00E-02
Clec4d	6	123262111..123275265	10.25	4.19	18.3	1.42E-04	0.04
Crlf1	8	70493158..70504081	23.02	4.19	18.21	7.50E-06	6.47E-03
Fpr1	17	complement(17876471..17883940)	10.27	4.18	18.15	7.11E-06	6.47E-03
Asprv1	6	86628164..86629710	14.44	4.13	17.49	7.77E-05	0.03
Fosl1	19	5447703..5455945	1.15	4.07	16.75	2.02E-05	0.01
F10	8	13037308..13056676	2.78	4	16.02	2.24E-04	0.05
Garem2	5	30105161..30118378	0.25	3.97	15.63	4.48E-06	4.50E-03
Tnn	1	complement(160085029..160153580)	0.12	3.9	14.95	6.52E-05	0.02
Olr1	6	complement(129485244..129507165)	0.81	3.9	14.93	7.82E-05	0.03
Ptx3	3	66219910..66225805	4.91	3.89	14.82	1.19E-06	1.57E-03
C8a	4	complement(104815679..104876398)	0.19	3.86	14.5	1.93E-04	0.04
Nxph4	10	complement(127525473..127534559)	1.13	3.84	14.31	3.72E-07	6.85E-04
Itgam_2	7	128062683..128128160	0.25	3.73	13.25	1.80E-04	0.04
Pcsk1	13	75089826..75134861	0.77	3.55	11.71	7.41E-06	6.47E-03
Retnlg	16	48872608..48874498	116.30	3.5	11.3	9.69E-05	0.03
Amn	12	111271095..111276426	0.83	3.47	11.06	9.57E-05	0.03
Stk32b	5	complement(37446825..37717171)	0.63	3.45	10.9	1.55E-04	0.04
Cntn2	1	complement(132509427..132543256)	0.94	3.44	10.86	5.86E-05	0.02
Ecel1	1	complement(87147655..87156521)	1.92	3.41	10.62	2.22E-04	5.00E-02
Dmp1	5	104202613..104214102	0.16	3.33	10.04	1.05E-04	3.00E-02
Pilra	5	complement(137821952..137836281)	4.75	3.3	9.84	1.36E-05	8.88E-03
Lrrc15	16	complement(30269302..30283256)	1.30	3.25	9.54	1.12E-04	0.03
Ptgs2	1	150100031..150108227	3.30	3.25	9.51	2.29E-04	0.05
Ifitm6	7	complement(141015699..141017924)	20.18	3.23	9.4	5.46E-05	0.02
Nos2	11	78920787..78960254	4.40	3.19	9.13	2.50E-05	0.01
Asic3	5	24413392..24417835	1.22	3.15	8.9	1.60E-04	0.04
AI593442	9	complement(52673042..52679780)	0.32	3.12	8.69	7.81E-05	0.03
Slc13a3	2	complement(165405028..165473230)	3.11	3	8.01	7.36E-05	0.03
Dio3	12	110279068..110281097	18.26	2.97	7.86	1.26E-05	8.66E-03
Kng2	16	complement(22985854..23029101)	7.83	2.93	7.6	2.62E-04	0.05
Mmp9	2	164940780..164955850	32.52	2.9	7.45	1.27E-05	8.66E-03
Sox11	12	complement(27334264..27342574)	0.07	2.84	7.18	2.37E-04	0.05
Wfdc17	11	83703991..83706268	58.82	2.71	6.54	1.83E-04	0.04
Cd300ld	11	complement(114982274..114989922)	3.88	2.59	6.04	6.33E-05	0.02

Mmp3	9	7445822..7455975	45.29	2.49	5.64	2.09E-04	5.00E-02
Spp1	5	104435118..104441050	54.11	2.49	5.63	2.13E-04	0.05
Tenm2	11	complement(36006656..37235964)	1.21	2.43	5.4	1.65E-04	0.04
Itgam_1	7	128062640..128118491	23.96	2.33	5.04	1.23E-04	0.03
P4ha3	7	100285520..100319699	20.04	2.27	4.83	4.52E-05	0.02
Pirb	7	complement(3711409..3720391)	19.87	2.13	4.39	7.72E-05	0.03
Sphkap	1	complement(83254139..83408200)	2.63	2.09	4.26	1.91E-04	4.00E-02
Serpine2	1	complement(79794197..79861180)	65.87	2.08	4.22	4.42E-06	4.50E-03
C5ar1	7	complement(16246743..16259540)	10.83	2.04	4.1	1.98E-04	0.04
Ntrk3	7	complement(78175959..78738012)	1.40	1.96	3.89	2.41E-04	0.05
Ndufa4l2	10	127514967..127517154	27.02	1.87	3.65	2.96E-05	0.01
Kcnma1	14	complement(23289431..24014491)	7.19	1.77	3.42	4.46E-07	7.15E-04
Rgs4	1	complement(169741477..169747642)	15.86	1.75	3.36	2.20E-04	0.05
Frzb	2	complement(80411970..80447625)	22.18	1.75	3.35	8.43E-05	0.03
Fn1	1	complement(71585520..71653200)	436.13	1.7	3.24	1.12E-05	8.65E-03
Bgn	X	73483602..73495933	471.95	1.68	3.2	4.24E-05	0.02
Nt5dc2	14	31131053..31139124	17.52	1.66	3.15	2.21E-04	0.05
Nav3	10	complement(109681259..110456204)	1.27	1.62	3.08	1.56E-04	4.00E-02
Basp1	15	complement(25363277..25413764)	25.18	1.62	3.07	2.31E-04	0.05
Col4a1	8	complement(11198423..11312826)	428.39	1.57	2.97	3.29E-05	0.02
Adamts12	15	11064790..11349231	12.13	1.56	2.96	2.37E-05	0.01
Susd2	10	complement(75636706..75644008)	28.89	1.51	2.84	1.42E-05	8.88E-03
Sulf1	1	12692277..12861192	28.14	1.48	2.79	1.77E-04	0.04
Medag	5	149411749..149431723	25.45	1.4	2.64	5.46E-05	0.02
Ttyh2	11	114675431..114720977	6.77	1.4	2.64	5.24E-05	2.00E-02
Col4a2	8	11312805..11449287	274.39	1.39	2.62	1.12E-04	0.03
Gpm6b	X	166238911..166388988	20.00	1.13	2.19	2.27E-04	0.05
Hoxb6	11	96292476..96301569	13.36	-1.23	-2.35	2.55E-04	5.00E-02
Igf2bp2	16	complement(22059009..22163299)	5.13	-1.31	-2.47	1.00E-04	0.03
Hoxb8	11	96281905..96285315	14.85	-1.37	-2.59	4.54E-05	0.02
Gbp3	3	142560026..142573209	16.90	-1.43	-2.69	1.47E-04	0.04
Dlx5	6	complement(6877805..6882085)	25.96	-1.56	-2.95	9.56E-05	0.03
Pax2	19	44756045..44837871	5.95	-1.64	-3.11	1.15E-04	0.03
Epcam	17	87635979..87651106	65.21	-1.74	-3.35	1.69E-04	0.04
Alox15	11	complement(70344152..70352031)	38.58	-1.77	-3.4	1.42E-04	0.04
Tgtp2	11	complement(49057194..49064206)	12.76	-1.87	-3.64	4.63E-05	0.02
Hmga2	10	complement(120361275..120476469)	3.53	-1.93	-3.82	2.36E-04	5.00E-02
Tgtp1	11	complement(48985327..48994172)	5.97	-1.93	-3.82	1.83E-04	0.04
Rec8	14	55618037..55625395	5.70	-2.23	-4.7	6.41E-05	0.02
Fzd10	5	128600844..128604093	6.57	-2.29	-4.89	1.72E-04	0.04
Wnt7a	6	complement(91363981..91411363)	10.23	-2.57	-5.93	1.40E-05	8.88E-03
Cxcl10	5	complement(92346638..92348889)	4.80	-2.67	-6.36	6.07E-05	0.02
Trpm1	7	64153835..64269775	0.19	-3.16	-8.95	2.04E-04	0.04
Tpm3-rs7	14	113314608..113316754	6.09	-3.18	-9.04	2.01E-05	1.00E-02
A2ml1	6	complement(128539821..128581608)	1.79	-3.18	-9.07	6.48E-05	0.02
Lgr5	10	complement(115450311..115587780)	1.84	-3.34	-10.12	1.82E-04	0.04
Ighv3-4	12	complement(114253617..114254051)	2.03	-4.71	-26.18	8.47E-05	0.03
Gdf1	8	70329817..70331587	0.52	-5.14	-35.38	2.11E-04	5.00E-02
Bhmt	13	complement(93616675..93637961)	0.22	-6.13	-70.23	2.03E-05	1.00E-02

blot Images for Fig 3a

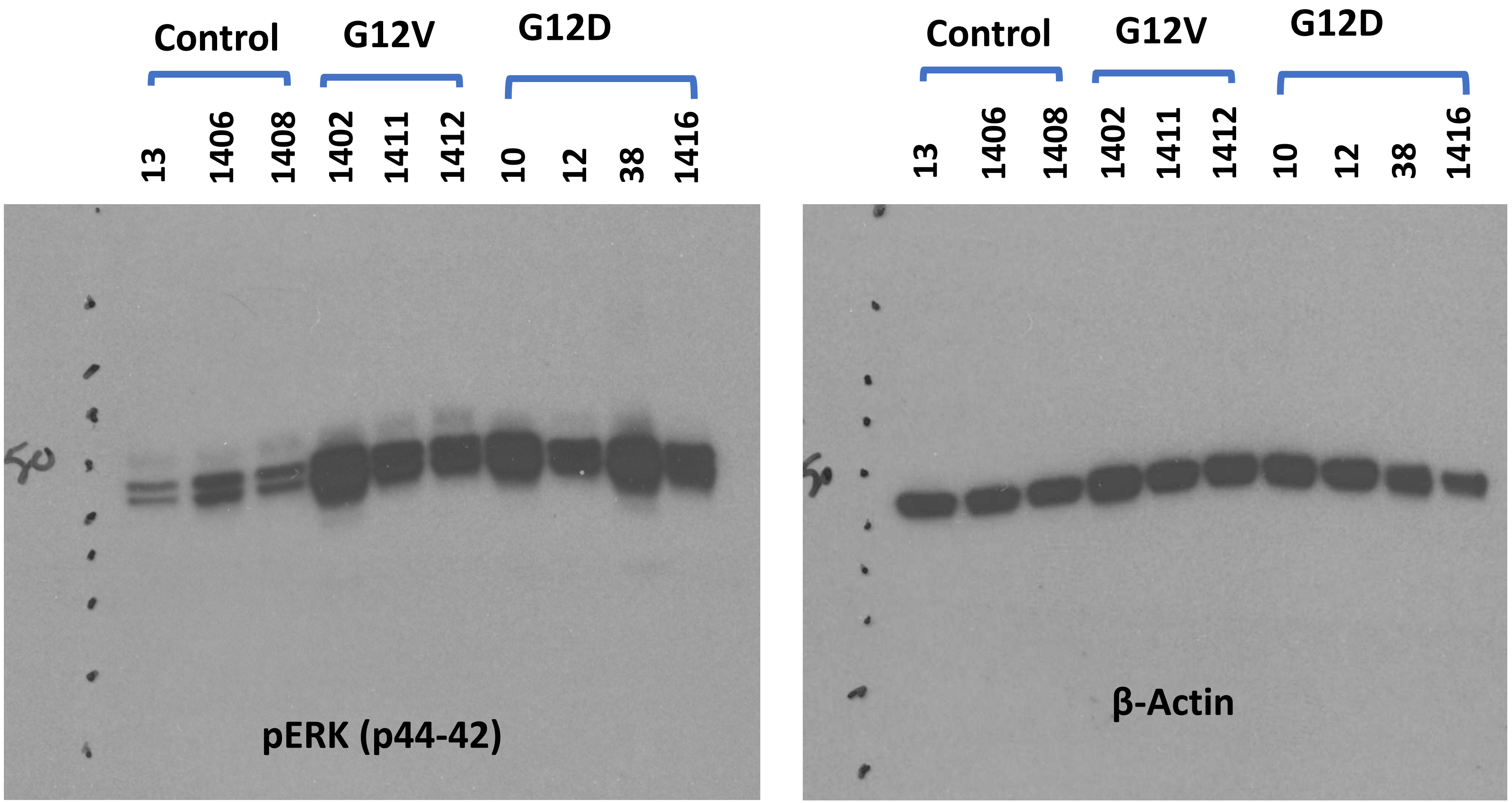

blot Images for Fig 3b

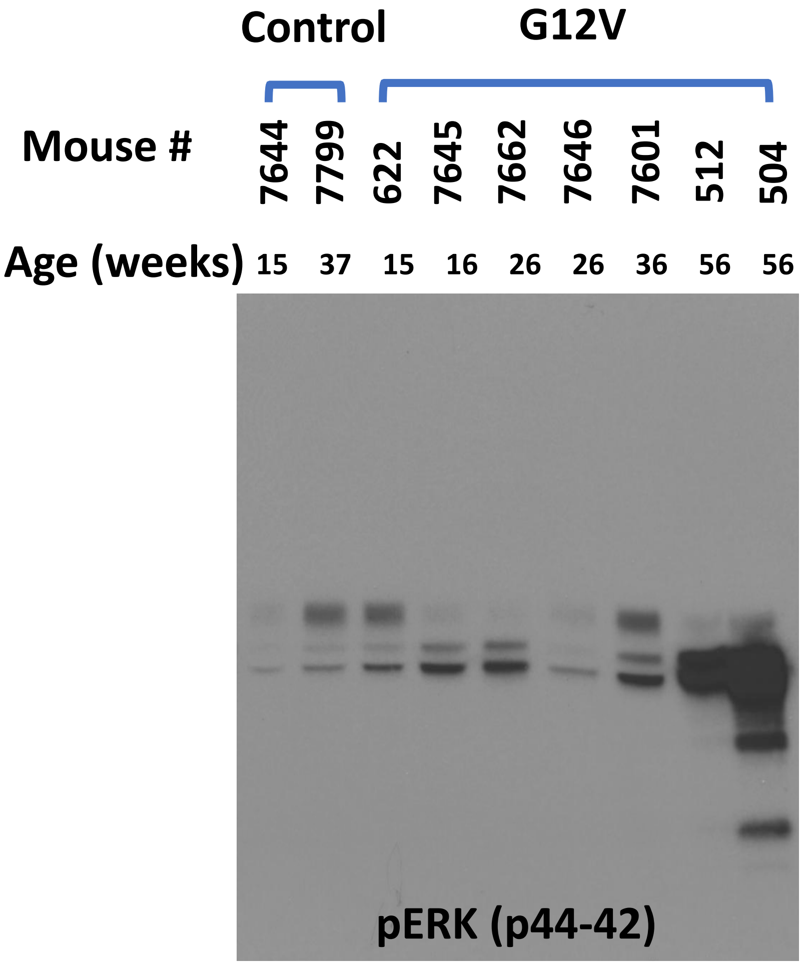

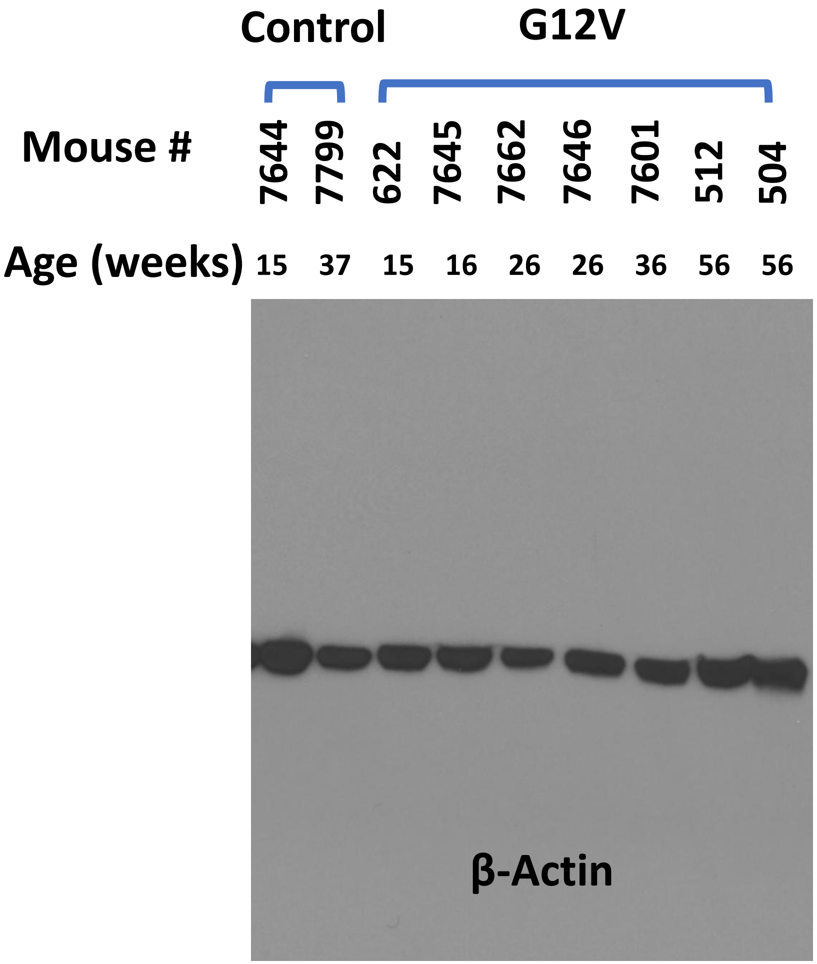

blot images for Figure S6

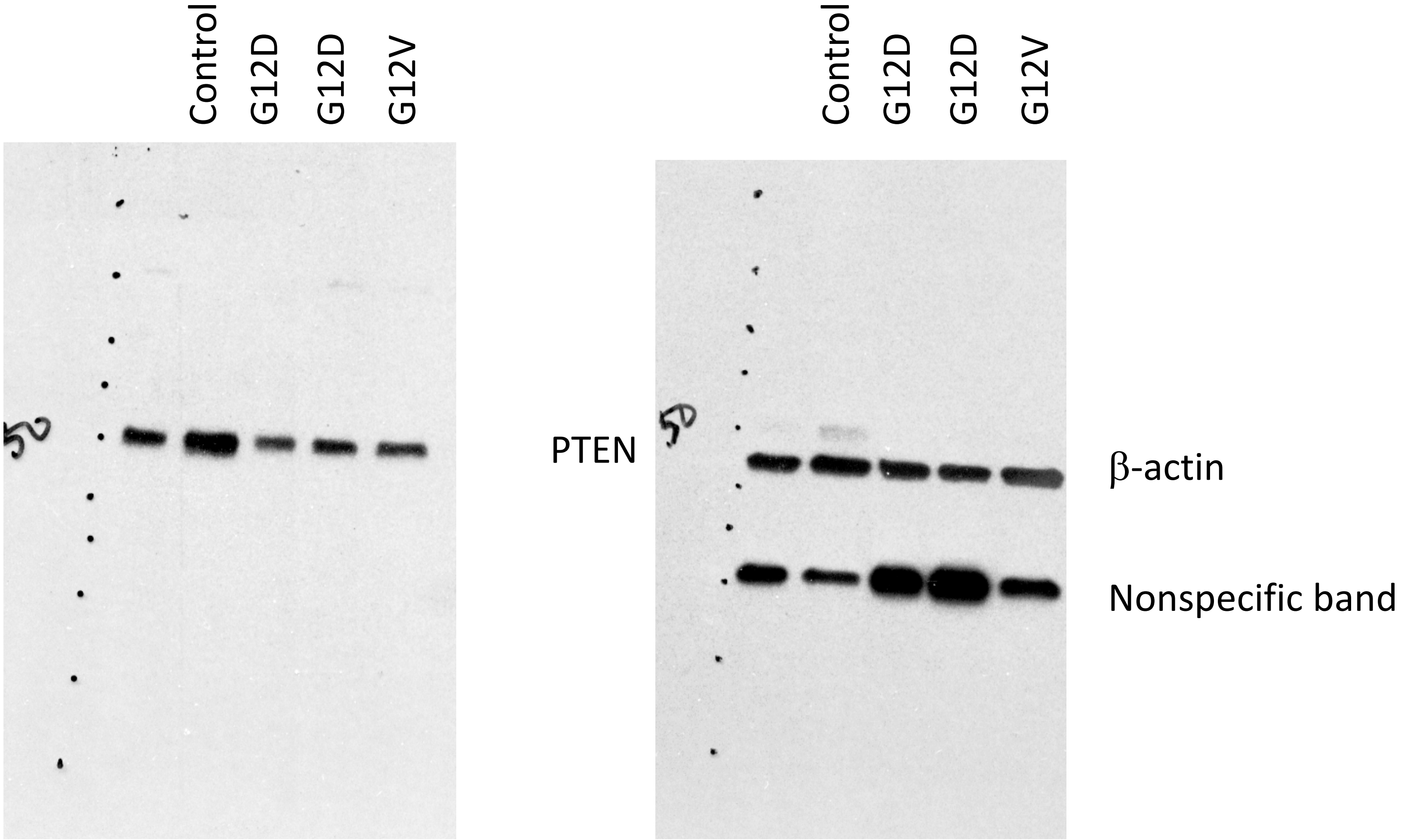

Supplement: Supplementary file 1 — Supplementary information. [file 41598_2020_77666_MOESM1_ESM.pdf]
