# Supplementary material for: Differences in gynecologic tumor development in Amhr2-Cre mice with KRASG12D or KRASG12V mutations
Source: Sci Rep. 2020 Nov 26;10:20678. doi: 10.1038/s41598-020-77666-y (PMC7693266; doi:10.1038/s41598-020-77666-y)
Supplement: Supplementary file 2 — Supplementary Legends. [file 41598_2020_77666_MOESM2_ESM.docx]

Figure S1. Images of the gross morphology of the gynecologic tumors that developed in G12D and G12V mice.

Figure S2. Immunohistostaining for cytokeratin 8 and ⍺-inhibin in ovarian tumors that developed in a *Pten^fl/fl^ LSL-Kras****^G12D/+^*** *Amhr2-Cre* mouse (27 weeks old) and a *Pten^fl/fl^ LSL-Kras^G12V/+^ Amhr2-Cre* mouse (36 weeks old).

Figure S3. Ki-67 staining of mouse leiomyoma sections. The high cellularity and Ki-67 staining suggest that the mouse leiomyomas are the atypical cellular leiomyoma and mitotically active leiomyoma subtypes.

Figure S4. Reverse transcription quantitative PCR analysis of ESR1 mRNA expression in uterine tissues from control, G12D, and G12V mice. The welch’s t test of significance and figure was generated with GraphPad Prism version 8.0.0 for macOS, GraphPad Software, San Diego, California USA, www.graphpad.com.

Figure S5. Beta-galactosidase immunostaining to confirm the expression of Cre in fallopian, ovarian and uterine tissues.

Figure S6. Confirmation of deletion of Pten in mice uterine tissues. **a)** Sequencing reads from the RNAseq data mapped to the *Pten* mRNA transcript. For the mouse with Amhr2-Cre Pten^fl/fl^ genotype, only 30% of the *Pten* transcripts contain the exon 5 region. On the other hand, the mouse with Amhr2-Cre Pten^fl/+^ genotype, 70% of the Pten mRNA transcipts contain the exon 5 region. **b)** Western blot of Pten in protein lysate extracted from the whole uterus isolated from control mice (Pten ^fl/fl^ KRAS^G12Dfl/+^), G12D mice (Amhr2-Cre Pten ^fl/fl^ KRAS^G12D/+^) and G12V mouse (Amhr2-Cre Pten ^fl/fl^ KRAS^G12V/+^). Mice with Amhr2-Cre had lower expression of Pten protein.

**Tables**

Supplementary Table S1. RPPA protein expression data for G12D, G12V, and control mice.

Supplementary Table S2. Differentially expressed between G12D mice (Amhr2-Cre Pten^fl/fl^ Kras^G12D/+)^ versus control mice (Amhr2-Cre Pten^fl/fl^). Differential gene expression analysis for two-group comparison was implemented in CLC Genome Workbench using the edgeR package, version 3.4.0.

Supplementary Table S3. Differentially expressed between G12V mice (Amhr2-Cre Pten^fl/fl^ Kras^G12V/+)^ versus control mice (Amhr2-Cre Pten^fl/fl^). Differential gene expression analysis for two-group comparison
